# Surrogate Assisted Semi-supervised Inference for High Dimensional Risk Prediction

**Published:** 2023

**Authors:** Jue Hou, Zijian Guo, Tianxi Cai

**Affiliations:** Division of Biostatistics, University of Minnesota School of Public Health, Minneapolis, MN 55455, USA; Department of Statistics, Rutgers University, Piscataway, NJ 08854-8019, USA; Department of Biostatistics, Harvard T.H. Chan School of Public Health, Boston, MA 02115, USA

**Keywords:** generalized linear models, high dimensional inference, model mis-specification, risk prediction, semi-supervised learning

## Abstract

Risk modeling with electronic health records (EHR) data is challenging due to no direct observations of the disease outcome and the high-dimensional predictors. In this paper, we develop a surrogate assisted semi-supervised learning approach, leveraging small labeled data with annotated outcomes and extensive unlabeled data of outcome surrogates and high-dimensional predictors. We propose to impute the unobserved outcomes by constructing a sparse imputation model with outcome surrogates and high-dimensional predictors. We further conduct a one-step bias correction to enable interval estimation for the risk prediction. Our inference procedure is valid even if both the imputation and risk prediction models are misspecified. Our novel way of ultilizing unlabelled data enables the high-dimensional statistical inference for the challenging setting with a dense risk prediction model. We present an extensive simulation study to demonstrate the superiority of our approach compared to existing supervised methods. We apply the method to genetic risk prediction of type-2 diabetes mellitus using an EHR biobank cohort.

## 1. Introduction

Precise risk prediction is vitally important for successful clinical care. High risk patients can be assigned to more intensive monitoring or intervention to improve outcome. Traditionally, risk prediction models are developed based on cohort studies or registry data. Population-based disease registries, while remain a critical source for epidemiological studies, collect information on a relatively small set of pre-specified variables and hence may limit researchers’ ability to develop comprehensive risk prediction models (Warren and Yabroff, 2015). Most clinical care is delivered in healthcare systems (Thompson et al., 2015), and electronic health records (EHR) embedded in healthcare systems accrue rich clinical data in broad patient populations. EHR systems centralize the data collected during routine patient care including structured elements such as codes for International Classification of Diseases, medication prescriptions, and medical procedures, as well as free-text narrative documents such as physician notes and pathology reports that can be processed through natural language processing for analysis. EHR data is also often linked with biobanks which provide additional rich molecular information to assist in developing comprehensive risk prediction models for a broad patient population.

Risk modeling with EHR data, however, is challenging due to several reasons. First, precise information on clinical outcome of interest, Y, is often embedded in free-text notes and requires manual efforts to extract accurately. Readily available outcome surrogates S, such as the diagnostic codes or mentions of the outcome, may be predictive of the true outcome Y, can deviate from the true label Y. Here we consider the general situation that a vector of surrogates, S, that are noisy error prone proxies of Y and may include non-informative surrogates. For example, using EHR data from Mass General Brigham, we found that the positive predictive value was only 0.48 and 0.19 for having at least 1 diagnosis code of Type II Diabetes Mellitus (T2DM) and for having at least 1 mention of T2DM in medical notes, respectively. Directly using these EHR proxies as true disease status to derive risk models may lead to substantial biases. On the other hand, extracting precise disease status requires manual chart review which is not feasible at a large scale. It is thus of great interest to develop risk prediction models under a semi-supervised learning (SSL) framework using both a large unlabeled dataset of size N containing information on predictors X along with surrogates S and a small labeled dataset of size n with additional observations on Y curated via chart review. Throughout the paper, we impose no stringent model assumptions on the triplet (Y,X,S) while using generalized linear working models to define and estimate the risk prediction model (see Section 2).

Additional challenges arise from the high dimensionality of the predictor vector X, and the potential model mis-specifications. Although much progress has been made in high dimensional regression in recent years, there is a paucity of literature on high dimensional inference under the SSL setting. Precise estimation of the high dimensional risk model is even more challenging if the risk model is not sparse. Allowing the risk model to be dense is particularly important when X includes genomic markers since a large number of genetic markers appear to contribute to the risk of complex traits (Frazer et al., 2009). For example, Vujkovic et al. (2020) recently identified 558 genetic variants as significantly associated with T2DM risk. An additional challenge arises when the fitted risk model is mis-specified, which occurs frequently in practice especially in the high dimensional setting. Model mis-specifications can also lead to the fitted model of Y∣X to be dense. There are limited methods currently available to make inference about high dimensional risk prediction models in the SSL setting especially under a possibly mis-specified dense model. In this paper, we fill in the gap by proposing an efficient surrogate assisted SSL (SAS) prediction procedure that leverages the fully observed surrogates S to make inference about a high dimensional risk model under such settings.

Our proposed estimation and inference procedures are as follows. For estimation, we first use the labelled data to fit a regularized imputation model with surrogates and high-dimensional covariates; then we impute the missing outcomes for the unlabeled data and fit the risk model using the imputed outcome and high-dimensional predictors. For inference, we devise a novel bias correction method, which corrects the bias due to the regularization for both imputation and estimation. Compared to existing literature, the key advantages of our proposed SAS procedure are

1. Applicable to dense risk model Y∣X: we allow the working risk model Y∣X to be dense as long as the working imputation model Y∣S,X is sparse;
2. Robustness to model mis-specification: the working models on both risk prediction Y∣X and imputation Y∣S,X can be mis-specified;
3. Requires no assumptions on the measurement error in S as proxies of Y and allows S itself to be of high dimension;
4. Our analysis on Lasso with estimated inputs in loss (see (6) and (20)) facilitates the consistency analysis for a dense model independent of the convergence rate of the consistently estimated inputs. The technique is an independent contribution to the high-dimensional statistics literatures.

The sparsity assumption on the imputation model is less stringent since we anticipate that most information on Y can be well captured by the low dimensional S while the fitted model of Y∣X might be dense under possible model mis-specifications. Our theory uncovers that suitable use of unlabeled data may greatly relax the sparsity of Y∣X. As most literatures in SSL emphasized in the efficiency gain, our work opens a new direction of estimiability expansion through SSL.

### 1.1 Related Literatures

Under the supervised setting where both Y and X are fully observed, much progress has been made in recent years in the area of high dimensional inference. High dimensional regression methods have been developed for commonly used generalized linear models under sparsity assumptions on the regression parameters (van de Geer and Bühlmann, 2009; Negahban et al., 2010; Huang and Zhang, 2012). Recently, Zhu and Bradic (2018b) studied the inference of linear combination of coefficients under dense linear model and sparse precision matrix. Inference procedures have also been developed for both sparse (Zhang and Zhang, 2014; Javanmard and Montanari, 2014; van de Geer et al., 2014) and dense combinations of the regression parameters (Cai et al., 2019; Zhu and Bradic, 2018a). High-dimensional inference under the logistic regression model has also been studied recently (van de Geer et al., 2014;Ma et al., 2020; Guo et al., 2020).

Under the SSL setting with n≪N, however, there is a paucity of literature on high dimensional inference. Although the SSL can be viewed as a missing data problem, it differs from the standard missing data setting in a critical way. Under the SSL setting, the missing probability tends to 1, which would violate a key assumption required in the missing data literature (Bang and Robins, 2005; Smucler et al., 2019; Chakrabortty et al., 2019, e.g.). Existing work on SSL with high-dimensional covariates largely focuses on the post-estimation inference on the global parameters under sparse linear models with examples including SSL estimation of population mean (Zhang et al., 2019; Zhang and Bradic, 2021), the explained variance (Cai and Guo, 2020), and the average treatment effect (Cheng et al., 2018; Kallus and Mao, 2020). Our SAS procedure is among the first attempts to conduct the semi-supervised inference of the high-dimensional coefficient and the individual prediction in the high-dimensional dense and possibly mis-specified risk prediction model. In a concurrent work, Deng et al. (2020) studied the efficient SSL estimation of high-dimensional linear models. Our work differs from them in at least three ways: 1) we consider the more flexible generalized linear models; 2) our setting involves the surrogates S, characterizing the imprecise data in EHR; 3) we study dense coefficients whose number of nonzero elements exceeds the number of labels. In high-dimensional regression with missing data, another line of work studied the estimation of linear models with missing or noisy covariates X (Loh and Wainwright, 2011; Belloni et al., 2017; Chandrasekher et al., 2020).

The surrogates S can be viewed conceptually as “mis-measured” proxies of the true outcome Y. Semi-supervised methods have been developed under the assumption that S depends on X only through Y, which essentially assumes an independent measurement error in S. For example, Gronsbell et al. (2019) studied the generalized linear risk prediction model using mis-measured S. With a single S, Zhang et al. (2022) considered high-dimensional generalized linear model for the prediction model allowing the independence assumption to be slightly violated. Our SAS approach differs from the measurement error approach in two fundamental aspects: 1) typical measurement error approaches require S to be the single proxy outcome of the same type as Y while our SAS approach allow a vector S of arbitrary types as long as some of them are predictive for Y; 2) measurement error approaches impose stringent independence and model assumptions on the triplet (S,X,Y) while our SAS approach has neither. Violation of the two requirements may obstruct the deployment of measurement error methods or compromise its performance.

### 1.2 Organization of the Paper

The remainder of the paper is organized as follows. We introduce our population parameters and model assumptions in Section 2. In Section 3, we propose the SAS estimation method along with its associated inference procedures. In Section 4, we state the theoretical guarantees of the SAS procedures, whose proofs are provided in the Supplementary Materials. We also remark on the sparsity relaxation and the efficiency gain of the SSL. In Section 5, we present simulation results highlighting finite sample performance of the SAS estimators and comparisons to existing methods. In Section 6, we apply the proposed method to derive individual risk prediction for T2DM using EHR data from Mass General Brigham.

## 2. Settings and Notations

For the i-th observation, $Y_{i}\in R$ denotes the outcome variable, $S_{i}\in R^{q}$ denotes the surrogates for $Y_{i}$ and $X_{i}\in R^{p+1}$ denotes the high-dimensional covariates with the first element being the intercept. Under the SSL setting, we observe n independent and identically distributed (i.i.d.) labeled observations, $ℒ=\left\{{\left(Y_{i},X_{i}^{⊤},S_{i}^{⊤}\right)}^{⊤},i=1,\ldots ,n\right\}$ and (N-n) i.i.d unlabeled observations, $𝒰=\left\{W_{i}={\left(X_{i}^{⊤},S_{i}^{⊤}\right)}^{⊤},i=n+1,\ldots ,N\right\}$. We assume that the labeled subjects are randomly sampled by design and the proportion of labelled sample is n/N=ρ∈(0, 1) with ρ→0 as n→∞. We focus on the high-dimensional setting where dimensions p and q grow with n and allow p+q to be larger than n. Motivated by our application, our main focus is on the setting N much larger than p, but our approach can be extended to N≤p under specific conditions.

To predict $Y_{i}$ with $X_{i}$, we consider a possibly mis-specified working regression model with a known monotone and smooth link function g,

(1) $$Y_{i}\sim g\left(\beta^{⊤}X_{i}\right).$$

We identify the target parameter as the most predictive working model measured by the pseudo log-likelihood ℓ(y, x)

(2) $$\beta_{0}=\underset{\beta }{argmin}-E\left\{\ell \left(Y_{i},\beta^{⊤}X_{i}\right)\right\},\ell (y,x)=yx-G(x),G^{\prime }(x)=g(x).$$

Here we do not assume any model for the true conditional expectation $E\left(Y_{i}|X_{i}\right)$. Our goal is to accurately estimate the high-dimensional parameter $\beta_{0}$, alternatively characterized by the first order condition for (2),

(3) $$E\left[X_{i}\left\{Y_{i}-g\left(\beta_{0}^{⊤}X_{i}\right)\right\}\right]=0.$$

Our procedure generally allows for a wide range of link functions and detailed requirements on g(⋅) and its anti-derivative G are given in Section 4. In our motivating example, Y is a binary indicator of T2DM status and $g(x)=1/\left(1+e^{-x}\right)$ with $G(x)=\text{log}\left(1+e^{x}\right)$. We shall further construct confidence intervals for $g\left(\beta_{0}^{⊤}x_{\text{new}}\right)$ with any $x_{\text{new}}\in R^{p+1}$. The predicted outcome $g\left(\beta_{0}^{⊤}x_{\text{new}}\right)$ can be interpreted as the maximum pseudo log-likelihood prediction under working model $g\left(\beta^{⊤}x_{\text{new}}\right)$. We make no assumption on the sparsity of $\beta_{0}$ relative to number of labels n, and hence it is not feasible to perform valid supervised learning for $\beta_{0}$ when $s_{\beta }={∥\beta_{0}∥}_{0}>n$.

We shall derive an efficient SSL estimate for $\beta_{0}$ by leveraging 𝒰. To this end, we fit a working imputation model

(4) $$Y_{i}\sim g\left(\gamma^{⊤}W_{i}\right),$$

whose limiting parameter is likewise defined as the most predictive working model

(5) $$\gamma_{0}=\underset{\gamma }{argmin}-E\left\{\ell \left(Y_{i},\gamma^{⊤}W_{i}\right)\right\}\Rightarrow E\left[W_{i}\left\{Y_{i}-g\left(\gamma_{0}^{⊤}W_{i}\right)\right\}\right]=0.$$

The definition of γ guarantees

(6) $$E\left[X_{i}\left\{Y_{i}-g\left(\gamma_{0}^{⊤}W_{i}\right)\right\}\right]=0.$$

and hence if we impute $Y_{i}$ as ${\overset{‾}{Y}}_{i}=g\left(\gamma_{0}^{⊤}W_{i}\right)$, we have $E\left[X_{i}\left\{{\overset{‾}{Y}}_{i}-g\left(\beta_{0}^{⊤}X_{i}\right)\right\}\right]=0$ regardless the adequacy of the imputation model (4) for the conditional mean $E\left(Y_{i}|W_{i}\right)$. It is thus feasible to carry out an SSL procedure by first deriving an estimate for ${\overset{‾}{Y}}_{i}$ using the labelled data ℒ and then regressing the estimated ${\overset{‾}{Y}}_{i}$ against $X_{i}$ using the whole data ℒ∪𝒰. Although we do not require $\beta_{0}$ to be sparse or any of the fitted models to hold, we do assume that $\gamma_{0}$ defined in (5) to be sparse. When the surrogates S are strongly predictive for the outcome, the sparsity assumption on $\gamma_{0}$ is reasonable since the majority of the information in Y can be captured in S.

**Notations.** We focus on the setting where min{n,p+q,N}→∞. For convenience, we shall use n→∞ in the asymptotic analysis. For two sequences of random variables $A_{n}$ and $B_{n}$, we use $A_{n}=O_{p}\left(B_{n}\right)$ and $A_{n}=o_{p}\left(B_{n}\right)$ to denote ${lim}_{c\to \infty }{lim}_{n\to \infty }P(|A|\geq c|B|)=0$ and ${lim}_{c\to 0}{lim}_{n\to \infty }P(|A|\geq c|B|)=0$, respectively. For two positive sequences $a_{n}$ and $b_{n},\;a_{n}=O\left(b_{n}\right)$ or $b_{n}≳a_{n}$ means that ∃C>0 such that $a_{n}\leq Cb_{n}$ for all $n;\;a_{n}≍b_{n}$ if $a_{n}=O\left(b_{n}\right)$ and $b_{n}=O\left(a_{n}\right)$, and $a_{n}\ll b_{n}$ or $a_{n}=o\left(b_{n}\right)$ if ${lim\;sup}_{n\to \infty }a_{n}/b_{n}=0$. We use $Z_{n}\overset{ℒ}{\to }N(0,\;1)$ to denote the sequence of random variables $Z_{n}$ converges in distribution to a standard normal random variable.

## 3. Methodology

### 3.1 SAS Estimation of $\beta_{0}$

The SAS estimation procedure for $\beta_{0}$ consists of two key steps: (i) fitting the imputation model to ℒ to obtain estimate $\hat{\gamma }$ for $\gamma_{0}$ defined in (5); and (ii) estimating $\beta_{0}$ in (3) by fitting imputed outcome ${\hat{Y}}_{i}=g\left({\hat{\gamma }}^{⊤}W_{i}\right)$ against $X_{i}$ to 𝒰. In both steps, we devise the Lasso type estimator to deal with the high-dimensionality of X. In principle, other types of variable selection methods, e.g. SCAD (Fan and Li, 2001) or square-root Lasso (Belloni et al., 2011), may also be used. We use the Lasso as the example for its simplicity. A further discussion on the choice of regularized estimators is given in Remark 6.

In Step (i), we estimate $\gamma_{0}$ by the $L_{1}$ regularized pseudo log-likelihood estimator $\hat{\gamma }$, defined as

(7) $$\hat{\gamma }=\underset{\gamma \in R^{p+q+1}}{argmin}\ell_{\text{imp}}\left(\gamma \right)+\lambda_{\gamma }{∥\gamma_{-1}∥}_{1}\;\text{ with }\;\lambda_{\gamma }≍\sqrt{\text{log}\left(p+q\right)/n},$$

where **a**_−1_ denotes the sub-vector of all the coefficients except for the intercept and

(8) $$\ell_{\text{imp}}\left(\gamma \right)=\frac{1}{n}\overset{n}{\underset{i=1}{\sum }}\ell \left(Y_{i},\gamma^{⊤}W_{i}\right)\text{ with }\ell \left(y,x\right)\text{ defined in }\left(2\right).$$

The imputation loss (8) corresponds to the negative log-likelihood when Y is binary and the imputation model holds with g being anti-logit. With $\hat{\gamma }$, we impute the unobserved outcomes for subjects in 𝒰 as ${\hat{Y}}_{i}=g\left({\hat{\gamma }}^{⊤}W_{i}\right)$, for n+1≤i≤N.

In Step (ii), we estimate $\beta_{0}$ by $\hat{\beta }=\hat{\beta }(\hat{\gamma })$, defined as,

(9) $$\hat{\beta }\left(\hat{\gamma }\right)=\underset{\beta \in R^{p+1}}{argmin}\ell^{†}\left(\beta ;\hat{\gamma }\right)+\lambda_{\beta }{∥\beta_{-1}∥}_{1}\;\text{ with }\;\lambda_{\beta }≍\sqrt{\text{log}\left(p\right)/N},$$

where $\ell^{†}(\beta ;\hat{\gamma })$ is the imputed pseudo log-likelihood:

(10) $$\ell^{†}\left(\beta ;\hat{\gamma }\right)=\frac{1}{N}\underset{i>n}{\sum }\ell \left({\hat{Y}}_{i},\beta^{⊤}X_{i}\right)+\frac{1}{N}\overset{n}{\underset{i=1}{\sum }}\ell \left(Y_{i},\beta^{⊤}X_{i}\right)\text{ with }\ell \left(y,x\right)\text{ defined in (2).}$$

We denote the complete data pseudo log-likelihood of the full data as

(11) $$\ell_{\text{PL}}\left(\beta \right)=\frac{1}{N}\overset{N}{\underset{i=1}{\sum }}\ell \left(Y_{i},\beta^{⊤}X_{i}\right).$$

and define the gradients of the various losses (8)–(11) as

(12) $${\dot{\ell }}_{\text{imp}}\left(\gamma \right)=\nabla \ell_{\text{imp}}\left(\gamma \right),{\dot{\ell }}_{PL}\left(\beta \right)=\nabla \ell_{PL}\left(\beta \right),{\dot{\ell }}^{†}\left(\beta ;\gamma \right)=\frac{\partial }{\partial \beta }\ell^{†}\left(\beta ;\gamma \right).$$

### 3.2 SAS Inference for Individual Prediction

Since g(⋅) is specified, the inference on $g\left(x_{\text{new}}^{⊤}\beta \right)$ immediately follows from the inference on $x_{\text{new}}^{⊤}\beta$. We shall consider the inference on standardized linear prediction $x_{\text{std}}^{⊤}\beta$ with the standardized covariates

$$x_{\text{std }}=x_{\text{new }}/{∥x_{\text{new }}∥}_{2}$$

and then scale the confidence interval back. This way, the scaling with ${∥x_{\text{new}}∥}_{2}$ is made explicit in the expression of the confidence interval.

The estimation error of $\hat{\beta }$ can be decomposed into two components corresponding to the respective errors associated with (7) and (9). Specifically, we write

(13) $$\hat{\beta }-\beta_{0}=\left\{\overset{-}{\beta }\left(\hat{\gamma }\right)-\beta_{0}\right\}+\left\{\hat{\beta }-\overset{-}{\beta }\left(\hat{\gamma }\right)\right\},$$

where $\overset{-}{\beta }(\hat{\gamma })$ is defined as the minimizer of the expected imputed loss conditionally on the labeled data, that is,

(14) $$\overset{-}{\beta }(\hat{\gamma })=\underset{\beta \in R^{p+1}}{argmin}E\left[\ell^{†}(\beta ;\hat{\gamma })|ℒ\right].$$

The term $\overset{-}{\beta }(\hat{\gamma })-\beta_{0}$ denotes the error from the imputation model in (7) while the term $\hat{\beta }-\overset{-}{\beta }(\hat{\gamma })$ denotes the error from the prediction model in (9) given the imputation model parameter $\hat{\gamma }$. As $\ell_{1}$ penalization is involved in both steps, we shall correct the regularization bias from the two sources. Following from the typical one-step debiasing LASSO (Zhang and Zhang, 2014), the bias $\hat{\beta }-\overset{-}{\beta }(\hat{\gamma })$ is estimated by $\hat{\Theta }{\dot{\ell }}^{†}(\hat{\beta };\hat{\gamma })$ where $\hat{\Theta }$ is an estimator of ${\left[E\left\{g^{\prime }\left(\beta_{0}^{⊤}X_{i}\right)X_{i}X_{i}^{⊤}\right\}\right]}^{-1}$, the inverse Hessian of $\ell^{†}(\cdot ;\hat{\gamma })$ at $\beta =\beta_{0}$.

The bias correction for $\overset{-}{\beta }(\hat{\gamma })-\beta_{0}$ requires some innovation since we need to conduct the bias correction for a nonlinear functional $\overset{-}{\beta }(\cdot )$ of LASSO estimator $\hat{\gamma }$, which has not been studied in the literature. We identify $\overset{-}{\beta }(\hat{\gamma })$ and $\beta_{0}$ by the first order moment conditions,

(15) $$\bar{\beta }\left(\hat{\gamma }\right):E_{i>n}\left[X_{i}\left\{g\left(\bar{\beta }{\left(\hat{\gamma }\right)}^{⊤}X_{i}\right)-g\left({\hat{\gamma }}^{⊤}W_{i}\right)\right\}|ℒ\right]\approx 0,\;\beta_{0}:E\left[X_{i}\left\{g\left(\beta_{0}^{⊤}X_{i}\right)-Y_{i}\right\}\right]=E\left[X_{i}\left\{g\left(\beta_{0}^{⊤}X_{i}\right)-g\left(\gamma_{0}^{⊤}W_{i}\right)\right\}\right]=0.$$

Here $E_{i>n}[\cdot |ℒ]$ denotes the conditional expectation of a single copy of the unlabeled data given the labelled data. By equating the two estimating equations in (15), we apply the first order approximation and approximate the difference $\overset{-}{\beta }(\hat{\gamma })-\beta_{0}$ by

(16) $$\overset{-}{\beta }(\hat{\gamma })-\beta_{0}\approx -{\left[E\left\{g^{\prime }\left(\beta_{0}^{⊤}X_{i}\right)X_{i}X_{i}^{⊤}\right\}\right]}^{-1}E_{i>n}\left[X_{i}\left\{g\left(\gamma_{0}^{⊤}W_{i}\right)-g\left({\hat{\gamma }}^{⊤}W_{i}\right)\right\}|ℒ\right]$$

Together with the bias correction for $\overset{-}{\beta }(\hat{\gamma })-\beta_{0}$, this motivates the debiasing procedure

$$\hat{\beta }-\frac{1-\rho }{n}\sum_{i=1}^{n}\hat{\Theta }X_{i}\left\{g\left({\hat{\gamma }}^{⊤}W_{i}\right)-Y_{i}\right\}-\hat{\Theta }{\dot{\ell }}^{†}\left(\hat{\beta };\hat{\gamma }\right).$$

The 1-ρ factor, which tends to one when n much smaller than N, comes from the proportion of unlabeled data whose missing outcome are imputed.

For theoretical considerations, we devise a cross-fitting scheme in our debiasing process. We split the labelled and unlabeled data into K folds of approximately equal size, respectively. The number of folds does not grow with dimension (e.g. K=10). We denote the indices sets for each fold of the labelled data ℒ as ${ℐ}_{1},\ldots ,{ℐ}_{K}$, and those of the unlabeled data 𝒰 as ${𝒥}_{1},\ldots ,{𝒥}_{K}$. We denote the respective sizes of each fold in the labelled data and full data as $n_{k}=\left|{ℐ}_{k}\right|$ and $N_{k}=n_{k}+\left|{𝒥}_{k}\right|$, where |ℐ| denotes the carnality of ℐ. Define ${ℐ}_{k}^{c}=\{1,\ldots ,n\}\setminus {ℐ}_{k}$ and ${𝒥}_{k}^{c}=\{n+1,\ldots ,N\}\setminus {𝒥}_{k}$. For each labelled fold k, we fit the imputation model with out-of-fold labelled samples:

(17) $${\hat{\gamma }}^{\left(k\right)}=\underset{\gamma \in {\mathbb{R}}^{p+q+1}}{\text{argmin}}\frac{1}{n-n_{k}}\underset{i\in {ℐ}_{k}^{c}}{\sum }\ell \left(Y_{i},\gamma^{⊤}W_{i}\right)+\lambda_{\gamma }{\left\|\gamma_{-1}\right\|}_{1}.$$

Using ${\hat{\gamma }}^{\left(k\right)}$, we fit the prediction model with the out-of-fold data ${ℐ}_{k}^{c}\cup {𝒥}_{k}^{c}$:

(18) $${\hat{\beta }}^{\left(k\right)}=\underset{\beta \in {\mathbb{R}}^{p+1}}{\text{argmin}}\frac{1}{N-N_{k}}\left[\underset{i\in {𝒥}_{k}^{c}}{\sum }\ell \left(g\left({\hat{\gamma }}^{\left(k\right)⊤}W_{i}\right),\beta^{⊤}X_{i}\right)+\underset{i\in {ℐ}_{k}^{c}}{\sum }\ell \left(Y_{i},\beta^{⊤}X_{i}\right)\right]+\lambda_{\beta }{\left\|\beta_{-1}\right\|}_{1}.$$

To estimate the projection

(19) $${\left.u_{0}=E\left\{g^{\prime }\left(\beta_{0}^{⊤}X_{i}\right)X_{i}X_{i}^{⊤}\right\}\right]}^{-1}X_{\text{std}},$$

we propose an $L_{1}$-penalized estimator

(20) $${\hat{u}}^{\left(k\right)}=\underset{u\in {\mathbb{R}}^{p}}{\text{argmin}}\frac{1}{N-N_{k}}\underset{k^{\prime }\neq k}{\sum }\underset{i\in {ℐ}_{k^{\prime }}\cup^{\text{​}}{𝒥}_{k^{\prime }}}{\sum }\left[\frac{1}{2}g^{\prime }\left({\hat{\beta }}^{\left(k,k^{\prime }\right)\text{T}}X_{i}\right){\left(X_{i}^{⊤}u\right)}^{2}-u^{⊤}x_{\text{std}}\right]+\lambda_{u}∥u{∥}_{1},$$

where ${\hat{\beta }}^{\left(k,k^{\prime }\right)}$ is trained with samples out of folds k and $k^{\prime }$,

(21) $${\hat{\beta }}^{\left(k,k^{\prime }\right)}=\underset{\beta \in {\mathbb{R}}^{p+1}}{\text{argmin}}\frac{\sum_{i\in {\left({𝒥}_{k}\cup^{\text{​}}{𝒥}_{k^{\prime }}\right)}^{c}}\ell \left(g\left({\hat{\gamma }}^{\left(k,k^{\prime }\right)⊤}W_{i}\right),\beta^{⊤}X_{i}\right)+\sum_{i\in {\left({ℐ}_{k}\cup^{\text{​}}{ℐ}_{k^{\prime }}\right)}^{c}}\ell \left(Y_{i},\beta^{⊤}X_{i}\right)}{N-N_{k}-N_{k^{\prime }}}+\lambda_{\beta }{\left\|\beta_{-1}\right\|}_{1},$$

with

$${\hat{\gamma }}^{\left(k,k^{\prime }\right)}=\underset{\gamma \in {\mathbb{R}}^{p+q+1}}{\text{argmin}}\frac{\sum_{i\in {ℐ}_{k}^{c}\cap^{\text{​}}{ℐ}_{k^{\prime }}^{c}}\ell \left(Y_{i},\gamma^{⊤}W_{i}\right)}{n-n_{k}-n_{k^{\prime }}}+\lambda_{\gamma }{\left\|\gamma_{-1}\right\|}_{1}.$$

The estimators in (21) take similar forms as those in (17) and (18) except that their training samples exclude two folds of data ${ℐ}_{k}\cup {𝒥}_{k}$ and ${ℐ}_{k^{\prime }}\cup {𝒥}_{k^{\prime }}$. In the summand of (20), the data $\left(Y_{i},X_{i},S_{i}\right)$ in fold $k^{\prime }{ℐ}_{k^{\prime }}\cup {𝒥}_{k^{\prime }}$ is independent of ${\hat{\beta }}^{\left(k,k^{\prime }\right)}$ trained without folds k and $k^{\prime }$. The estimation of u requires an estimator of β and both estimators are subsequently used for the debiasing step. Using the same set of data multiple times for $\hat{\beta },\;\hat{u}$, debiasing and variance estimation may induce over-fitting bias, so we implemented the cross-fitting scheme to reduce the over-fitting bias. As a remark, cross-fitting might not be necessary for theory with additional assumptions and/or empirical process techniques.

We obtain the cross-fitted debiased estimator for $x_{\text{std}}^{⊤}\beta$ as $\hat{x_{\text{std}}^{⊤}\beta }$, defined as

(22) $$\frac{1}{K}\overset{K}{\underset{k=1}{\sum }}x_{\text{std}}^{⊤}{\hat{\beta }}^{\left(k\right)}-\frac{1}{N}\overset{K}{\underset{k=1}{\sum }}\underset{i\in {𝒥}_{k}}{\sum }{\hat{u}}^{\left(k\right)⊤}X_{i}\left\{g\left({\hat{\beta }}^{\left(k\right)⊤}X_{i}\right)-g\left({\hat{\gamma }}^{\left(k\right)\text{T}}W_{i}\right)\right\}\;-\frac{1}{n}\overset{K}{\underset{k=1}{\sum }}\underset{i\in {ℐ}_{k}}{\sum }{\hat{u}}^{\left(k\right)⊤}X_{i}\left\{\left(1-\rho \right)\cdot g\left({\hat{\gamma }}^{\left(k\right)\text{T}}W_{i}\right)+\rho \cdot g\left({\hat{\beta }}^{\left(k\right)⊤}X_{i}\right)-Y_{i}\right\}.$$

The second term is used to correct the bias $\overset{-}{\beta }(\hat{\gamma })-\beta_{0}$ and the third term is used to correct the bias $\hat{\beta }-\overset{-}{\beta }(\hat{\gamma })$. The corresponding variance estimator is

(23) $${\hat{V}}_{\text{SAS}}=\frac{1}{n}\overset{K}{\underset{k=1}{\sum }}\underset{i\in {ℐ}_{k}}{\sum }{\left({\hat{u}}^{\left(k\right)\text{T}}X_{i}\right)}^{2}{\left\{\left(1-\rho \right)\cdot g\left({\hat{\gamma }}^{\left(k\right)\text{T}}W_{i}\right)+\rho \cdot g\left({\hat{\beta }}^{\left(k\right)\text{T}}X_{i}\right)-Y_{i}\right\}}^{2}\;+\frac{\rho^{2}}{n}\overset{K}{\underset{k=1}{\sum }}\underset{i\in {𝒥}_{k}}{\sum }{\left({\hat{u}}^{\left(k\right)\text{T}}X_{i}\right)}^{2}{\left\{g\left({\hat{\beta }}^{\left(k\right)\text{T}}X_{i}\right)-g\left({\hat{\gamma }}^{⊤}W_{i}\right)\right\}}^{2}$$

Through the link g and the scaling factor ${∥x_{\text{new}}∥}_{2}$, we estimate $g\left(x_{\text{new}}^{⊤}\beta_{0}\right)$ by $g\left({∥x_{\text{new}}∥}_{2}\hat{x_{\text{std}}^{⊤}\beta }\right)$ and construct the (1-α)×100% confidence interval for $g\left(x_{\text{new}}^{⊤}\beta_{0}\right)$ as

(24) $$\left[g\left\{{∥x_{\text{new}}∥}_{2}\left(\hat{x_{\text{std}}^{⊤}\beta }-{Ƶ}_{\alpha /2}\sqrt{{\hat{V}}_{\text{SAS}}/n}\right\}\right),g\left\{{∥x_{\text{new}}∥}_{2}\left(\hat{x_{\text{std}}^{⊤}\beta }+{Ƶ}_{\alpha /2}\sqrt{{\hat{V}}_{\text{SAS}}/n}\right)\right\}\right],$$

where $Z_{\alpha /2}$ is the 1-α/2 quantile of the standard normal distribution.

## 4. Theory

We introduce assumptions required for both estimation and inference in Section 4.1. We state our theories for estimation and inference, respectively in Sections 4.2 and 4.3.

### 4.1 Assumptions

We assume the complete data consist of i.i.d. copies of $\left(Y_{i},X_{i},S_{i}\right)$, for i=1,…,N. For our focused SSL settings, only the first n outcome labels $Y_{i},\ldots ,Y_{n}$ are observed. Under the i.i.d assumption, our SSL setting is equivalent to the missing completely at random (MCAR) assumption. The sparsities of $\gamma_{0},\;\beta_{0}$ and $u_{0}$ are denoted as

$$s_{\gamma }={∥\gamma_{0}∥}_{0},s_{\beta }={∥\beta_{0}∥}_{0},s_{u}={∥u_{0}∥}_{0}.$$

We focus on the setting with n, p+q, N→∞ with n being allowed to be smaller than p+q. We allow that $s_{\gamma },\;s_{\beta }$ and $s_{u}$ grow with n, p+q, N and satisfy $s_{\gamma }\ll n$ and $s_{\beta }+s_{u}\ll N$. While our method and theory adaptively applies to both SSL (N≫n) and missing data (N≍n) settings without prior knowledge on the limit of n/N, we emphasize on the SSL (N≫n) setting that matches our motivating EHR studies and is also less studied in the literature. To achieve the sharper dimension conditions, we consider the sub-Gaussian design as in Portnoy (1984, 1985); Negahban et al. (2010). We denote the sub-Gaussian norm for random variables and random vectors both as $∥\cdot {∥}_{\psi_{2}}$. The detailed definition is given in Appendix D.

**Assumption 1** *For constants* $\nu_{1},\;\nu_{2}$ *and* M *independent of* n, p *and* N,

- the residuals $Y_{i}-g\left(\gamma_{0}^{⊤}W_{i}\right)$ and $Y_{i}-g\left(\beta_{0}^{⊤}X_{i}\right)$ are sub-Gaussian random variables with sub-Gaussian norm bounded by ${∥Y_{i}-g\left(\gamma_{0}^{⊤}W_{i}\right)∥}_{\psi_{2}}\leq \nu_{1}$ and ${∥Y_{i}-g\left(\beta_{0}^{⊤}X_{i}\right)∥}_{\psi_{2}}\leq \nu_{2}$;
- The link function g satisfies the monotonicity and smoothness conditions: ${inf}_{x\in R}g^{\prime }(x)\geq 0,\;{sup}_{x\in R}g^{\prime }(x)<M$ and ${sup}_{x\in R}g^{\prime \prime }(x)<M$.

Under our motivating example with a binary $Y_{i}$ and $g(x)=e^{x}/\left(1+e^{x}\right)$, 1a and 1b are satisfied. The condition is also satisfied for the probit link function and the identity link function. Condition 1a is universal for high-dimensional regression. Admittedly, Lipschitz requirement in 1b rules out some generalized linear model links with unbounded derivatives like the exponential link, but we may substitute the condition by assuming a bounded ${∥X_{i}∥}_{\infty }$.

**Assumption 2** *For constants* $\sigma_{max}^{2}$ *and* $\sigma_{\text{min}}^{2}$ *independent of* n, p, N,

- $W_{i}$ is a sub-Gaussian vector with sub-Gaussian norm ${∥W_{i}∥}_{\psi_{2}}\leq \sigma_{max}/\sqrt{2}$;
- The weak overlapping condition at the population parameter $\beta_{0}$ and $\gamma_{0}$,
  - ${inf}_{∥v{∥}_{2}=1}v^{⊤}E\left(\left[g^{\prime }\left(\beta_{0}^{⊤}X_{i}\right)\wedge 1\right]X_{i}X_{i}^{⊤}\right)v\geq \sigma_{\text{min}}^{2}$,
  - ${inf}_{∥v{∥}_{2}=1}v^{⊤}E\left[\left\{g^{\prime }\left(\gamma_{0}^{⊤}W_{i}\right)\wedge 1\right\}W_{i}W_{i}^{⊤}\right]v\geq \sigma_{\text{min}}^{2}$;
- The non-degeneracy of average residual variance:

$$\underset{∥v{∥}_{2}=1}{inf}E\left[{\left\{Y_{i}-(1-\rho )\cdot g\left(\gamma_{0}^{⊤}W_{i}\right)-\rho \cdot g\left(\beta_{0}^{⊤}X_{i}\right)\right\}}^{2}{\left(X_{i}^{⊤}v\right)}^{2}\right]\geq \sigma_{\text{min}}^{2}.$$

Assumption 2a is typical for high-dimensional regression (Negahban et al., 2010), which also implies the bounded maximal eigenvalue of the second moment

$$\underset{∥v{∥}_{2}=1}{\text{sup}}v^{⊤}E\left[W_{i}W_{i}^{⊤}\right]v\leq \sigma_{\text{max}}^{2}.$$

Notably, we do not require two common conditions under high-dimensional generalized linear models (Huang and Zhang, 2012; van de Geer et al., 2014): 1) the upper bound on ${\text{sup}}_{i=1,\ldots ,N}{∥X_{i}∥}_{\infty }$; 2) the lower bound on ${inf}_{i=1,\ldots ,N}g^{\prime }\left(\beta_{0}^{⊤}X_{i}\right)$, often known as the overlapping condition for logistic regression model. Compared to the overlapping condition under logistic regression that $g\left(\beta_{0}^{⊤}X_{i}\right)$ and $g\left(\gamma_{0}^{⊤}W_{i}\right)$ are bounded away from zero, our Assumptions 2b and 2c are weaker because they are implied by the typical minimal eigenvalue condition

$$\underset{∥v{∥}_{2}=1}{inf}v^{⊤}E\left(W_{i}W_{i}^{⊤}\right)v\geq \sigma_{\text{min}}^{2}$$

plus the overlapping condition.

### 4.2 Consistency of the SAS Estimation

We now state the $L_{2}$ and $L_{1}$ convergence rates of our proposed SAS estimator.

**Theorem 1 (Consistency of SAS estimation)** *Under Assumptions 1, 2 and with*

(25) $$s_{\gamma }=o\left(n/\text{log}\left(p+q\right)\right),s_{\beta }=o\left(N/\text{log}\left(p\right)\right),\lambda_{\beta }≳\sqrt{\text{log}\left(p\right)/N},$$

*we have*

$${∥\hat{\beta }-\beta_{0}∥}_{2}\;=O_{p}\left(\sqrt{s_{\beta }}\lambda_{\beta }+(1-\rho )\sqrt{s_{\gamma }\text{log}(p+q)/n}\right),$$

$${∥\hat{\beta }-\beta_{0}∥}_{1}\;=O_{p}\left(s_{\beta }\lambda_{\beta }+(1-\rho {)}^{2}s_{\gamma }\text{log}(p+q)/\left(n\lambda_{\beta }\right)\right).$$

**Remark 2** *The dimension requirement for our SAS estimator achieving* $L_{2}$ *consistency significantly weakens the existing dimension requirement in the supervised setting* (Negahban et al., 2010; Huang and Zhang, 2012; Bühlmann and Van De Geer, 2011; Bickel et al., 2009) *With* $\lambda_{\beta }≍\sqrt{\text{log}(p)/N}$, *Theorem 1 implies the* $L_{2}$ *consistency of* $\overset{ˆ}{\beta }$ *under the dimension condition*,

(26) $$(1-\rho {)}^{2}s_{\gamma }\text{log}(p+q)/n+s_{\beta }\text{log}(p)/N=o(1)\text{.}$$

When N≫n, our requirement on the sparsity of $\beta ,\;s_{\beta }=o(N/\text{log}(p))$ is significantly weaker than $s_{\beta }=o(n/\text{log}(p))$, which is known as the fundamental sparsity limit to identify the high-dimensional regression vector in the supervised setting. Theorem 1 indicates that with assistance from observed S∈𝒰, the SAS procedure allows $s_{\beta }>n$ provided that N is sufficiently large and the imputation model is sparse. This distinguishes our result from most estimation results in high-dimensional supervised settings. Among SSL literatures, the utility of unlabeled data in relaxation of sparsity condition has never been conceived before.

**Remark 3** *In the context of Theorem 1, a sparse imputation, often induced by a small number of highly predictive surrogates, is essential for an optimal estimation rate. When* $s_{\beta }>s_{\gamma }$, *the* $L_{2}$ *rate in Theorem 1 has two components,* $\sqrt{s_{\beta }\text{log}(p)/N}$ *regarding the minimax rate to learn* β *from all data and* $\sqrt{s_{\gamma }\text{log}(p+q)/n}$ *regarding the minimax rate to learn* γ *in the labeled data* (Raskutti et al., 2011). *Thus, the rate cannot be further improved if the sparser imputation model is used to identify the denser* β *without additional conditions*.

**Remark 4** *If the* $L_{1}$ *consistency is of interest, the penalty levels are chosen as*

(27) $$\lambda_{\beta }≍max\left\{\sqrt{\text{log}\left(p\right)/N},\sqrt{s_{\gamma }/s_{\beta }}\lambda_{\gamma }\right\},$$

which produces the $L_{1}$ estimation rate from Theorem 1

$${∥\hat{\beta }-\beta_{0}∥}_{1}=O_{p}\left(s_{\beta }\sqrt{\text{log}(p)/N}+\sqrt{s_{\gamma }s_{\beta }\text{log}(p)/n}\right).$$

Compared to the condition for $L_{1}$ consistency under supervised learning, $s_{\beta }=o(\sqrt{n/\text{log}(p)})$, the condition from SAS estimation $s_{\beta }=o\left(\left(n/s_{\gamma }+N\right)/\text{log}(p)\right)$ allows a denser $\beta_{0}$ in the setting with a very sparse $\gamma_{0}$ and a large unlabeled data. On the other hand, the $L_{2}$ estimation rate in Theorem 1 remains the same if

$$\sqrt{\text{log}(p)/N}≲\lambda_{\beta }≲max\left\{\sqrt{\text{log}\left(p\right)/N},\sqrt{s_{\gamma }/s_{\beta }}\lambda_{\gamma }\right\}.$$

Our theory on the SAS inference procedure uses the $L_{2}$ instead of the $L_{1}$ consistency.

Theorem 1 implies the following prediction consistency result.

**Corollary 5 (Consistency of individual prediction)** *Suppose* $x_{\text{new}}$ *is sub-Gaussian random vector satisfying* ${\text{sup}}_{∥v{∥}_{2}=1}v^{⊤}E\left[x_{\text{new}}x_{\text{new}}^{⊤}\right]v\leq \sigma_{\text{max}}^{2}$. *Under the conditions of Theorem 1, we have*

$$g\left({\hat{\beta }}^{⊤}x_{\text{new}}\right)-g\left(\beta_{0}^{⊤}x_{\text{new}}\right)=O_{p}\left({∥\hat{\beta }-\beta_{0}∥}_{2}\right)=o_{p}(1).$$

The concentration result of Corollary 5 is established with respect to the joint distribution of the data and the new observation $x_{\text{new}}$. This is in a sharp contrast to the individual prediction conditioning on any new observation $x_{\text{new}}$. If the goal is to conduct inference for any given $x_{\text{new}}$, the theoretical justification is provided in the following Theorem 7 and Corollary 8.

**Remark 6** *Other types of penalties shown to provide consistent estimation in* $L_{2}$ *for the working imputation model can substitute the Lasso penalty in* (7)*, since the $L_{2}$ rate* ${∥\hat{\gamma }-\gamma_{0}∥}_{2}$ *is the only property invoked for* $\hat{\gamma }$ *in the proof of Theorem 1. For example, we may choose the square-root Lasso* (Belloni et al., 2011) *with pivotal recovery under linear models with identity link* g(x)=x. *Changing the Lasso penalty in* (9)*, however, might require a different proof to produce the stated estimation rate adaptive to arbitrary $s_{\beta }/N$ and* $s_{\gamma }/n$, *covering both* $s_{\beta }/N\ll s_{\gamma }/n$ *and* $s_{\beta }/N≳s_{\gamma }/n$ *settings (Case 1 and 2, respectively, in the Proof of Theorem 1). If the* $s_{\beta }/N\ll s_{\gamma }/n$ *setting guaranteed by a very large* N *alone is of interest, other penalties for $\hat{\beta }$ can work equally well (by adapting Case 1 in the Proof of Theorem 1)*.

### 4.3 $\sqrt{n}$-inference with Debiased SAS Estimator

We state the validity of our SSL inference in Theorem 7. We use to $A\overset{ℒ}{\to }B$ to denote that random variable A converges in distribution to a distribution B.

**Theorem 7 (SAS Inference)** *Let* $x_{\text{new}}$ *be the random vector representing the covariate of a new individual. Under Assumptions 1, 2 and the dimension condition*

(28) $$(1-\rho {)}^{4}\frac{s_{\gamma }^{2}\text{log}(p+q{)}^{2}}{n}+\frac{\rho \left(s_{\beta }^{2}+s_{\beta }s_{u}\right)\text{log}(p{)}^{2}}{N}+(1-\rho {)}^{2}\frac{s_{\gamma }s_{u}\text{log}\left(p+q\right)\text{log}\left(p\right)}{N}=o\left(1\right),$$

we draw inference on $x_{\text{new}}^{⊤}\beta_{0}$ conditionally on $x_{\text{new}}$ according to

$$\left.\sqrt{n}{\hat{V}}_{SAS}^{-1/2}\left(\hat{x_{\text{std}}^{⊤}\beta }-\frac{x_{\text{new}}^{⊤}\beta_{0}}{{∥x_{\text{new}}∥}_{2}}\right)\right|\;x_{\text{new}}\overset{ℒ}{\to }N(0,\;1),$$

where ${\hat{V}}_{\text{SAS}}^{2}$ defined in (23) is the estimator of the asymptotic variance

$$V_{\text{SAS}}=E\left[{\left(u_{0}^{⊤}X_{i}\right)}^{2}{\left\{Y-\left(1-\rho \right)\cdot g\left(\gamma_{0}^{⊤}W_{i}\right)-\rho \cdot g\left(\beta_{0}^{⊤}X_{i}\right)\right\}}^{2}\right]+\rho \left(1-\rho \right)E\left[{\left(u_{0}^{⊤}X_{i}\right)}^{2}{\left\{g\left(\gamma_{0}^{⊤}W_{i}\right)-g\left(\beta_{0}^{⊤}X_{i}\right)\right\}}^{2}\right],$$

with

(29) $$u_{0}=\Theta_{0}\frac{X_{\text{new}}}{{∥x_{\text{new}}∥}_{2}}={\left[E\left\{g^{\prime }\left(\beta_{0}^{⊤}X_{i}\right)X_{i}X_{i}^{⊤}\right\}\right]}^{-1}\frac{X_{\text{new}}}{{∥x_{\text{new}}∥}_{2}}.$$

By the Young’s inequality, the condition (28) is implied by

(30) $$(1-\rho {)}^{4}\frac{s_{\gamma }^{2}\text{log}(p+q{)}^{2}}{n}+\frac{\sqrt{\rho }\left(s_{\beta }+s_{u}\right)\text{log}(p)}{\sqrt{N}}=o(1),$$

When p is much smaller than the full sample size N, our condition (30) allows the sparsity levels of $\beta_{0}$ and $u_{0}$ to be as large as p. Even if p is larger than N, our SAS inference procedure is valid if $s_{\beta }+s_{u}≲\sqrt{N}/\text{log}(p)$. In the literature on confidence interval construction in high-dimensional supervised setting, the valid inference procedure for a single regression coefficient in the linear regression requires $s_{\beta }≲\sqrt{n}/\text{log}(p)$ (Zhang and Zhang, 2014; Javanmard and Montanari, 2014; van de Geer et al., 2014). Such a sparsity condition has been shown to be necessary to construct a confidence interval of a parametric rate (Cai and Guo, 2017). We have leveraged the unlabeled data to significantly relax the fundamental limit of statistical inference from $s_{\beta }≲\sqrt{n}/\text{log}(p)$ to $s_{\beta }≲N/\{\text{log}(p)\sqrt{n}\}$. The amount of labelled data validates the statistical inference for a dense model in high dimensions.

The sparsity of $u_{0}$ is determined by $x_{\text{new}}$ and the precision matrix $\Theta_{0}$. In the supervised learning setting, for confidence interval construction for a single regression coefficient, van de Geer et al. (2014) requires $s_{u}≲n/\text{log}(p)$ is required. According to (30), our SAS inference requires $s_{u}≲N/\{\text{log}(p)\sqrt{n}\}$, which can be weaker than $s_{u}≲n/\text{log}(p)$ if the amount of unlabeled data is larger than $n^{2}$. Theorem 7 implies that our proposed CI in (24) is valid in terms of coverage, which is summarized in the following corollary.

**Corollary 8** *Under Assumptions 1 and 2, as well as* (28), *the CI defined in* (24) *satisfies,*

$$P\{g(\left.{∥x_{\text{new }}∥}_{2}\left(\hat{x_{\text{std }}^{⊤}\beta }-{Ƶ}_{\alpha /2}\sqrt{{\hat{V}}_{\text{SAS }}/n}\right)\right)\leq g\left(x_{\text{new }}^{⊤}\beta_{0}\right)\left.\;\leq g\left(\left({∥x_{\text{new }}∥}_{2}\hat{x_{\text{std }}^{⊤}\beta }+{Ƶ}_{\alpha /2}\sqrt{{\hat{V}}_{\text{SAS }}/n}\right)\right)\right\}=1-\alpha +o(1).\;2g^{\prime }\left(\beta_{0}^{⊤}x_{\text{new }}\right){∥x_{\text{new }}∥}_{2}{Ƶ}_{\alpha /2}\sqrt{V_{\text{SAS }}/n}≲{∥x_{\text{new }}∥}_{2}/\sqrt{n},$$

where $V_{SAS}$ is the the asymptotic variance defined in (29).

Confidence interval construction for $g\left(x_{\text{new}}^{⊤}\beta_{0}\right)$ in high-dimensional supervised setting has been recently studied in Guo et al. (2020). Guo et al. (2020) assumes the prediction model to be correctly specified as a high-dimensional sparse logistic regression and the inference procedure is valid if $s_{\beta }≲\sqrt{n}/\text{log}\;p$. In contrast, we leverage the unlabeled data to allow for mis-specified prediction model and a dense regression vector, as long as the dimension requirement in (28) is satisfied.

### 4.4 Efficiency comparison of SAS Inference

Efficiency in high-dimensional setting or SSL setting in which the proportion of labelled data decays to zero is yet to be formalized. Here we use the efficiency bound in the classical low-dimensional with a fixed ρ as the benchmark. Apart from the relaxation of various sparsity conditions, we illustrate next that our SAS inference achieves a decent efficiency with properly specified imputation model compared to the supervised learning and the benchmark.

Similar to the phenomenon discovered by Chakrabortty and Cai (2018), if the imputation model is correct, we can guarantee the efficiency gain by SAS inference in comparison to the asymptotic variance of the supervised learning,

(31) $$V_{SL}=E\left[{\left(u_{0}^{⊤}X_{i}\right)}^{2}{\left\{Y_{i}-g\left(\beta_{0}^{⊤}X_{i}\right)\right\}}^{2}\right].$$

**Proposition 9** *If* $E\left(Y_{i}|S_{i},X_{i}\right)=g\left(\gamma_{0}^{⊤}W_{i}\right)$, *we have* $V_{SL}\geq V_{SAS}$.

Moreover, we can show that our SAS inference attains the benchmark efficiency derived from classical fixed ρ setting (Tsiatis, 2007). To simplify the derivation, we describe the missing-completely-at-random mechanism through the binary observation indicator $R_{i},i=1,\ldots ,N$, independent of $Y_{i},\;X_{i}$ and $S_{i}$. We still denote the proportion of labelled data as $\rho =E\left(R_{i}\right)$. The unsorted data take the form

$$𝒟=\left\{D_{i}={\left(X_{i}^{⊤},S_{i}^{⊤},R_{i},R_{i}Y_{i}\right)}^{⊤},i=1,\ldots ,N\right\}.$$

We consider the following class of complete data semi-parametric models

(32) $${ℳ}_{\text{comp}}=\left\{f_{X,Y,S,R}\left(x,y,s,r\right)=f_{X}\left(x\right)\rho^{r}{\left(1-\rho \right)}^{1-r}f_{Y|S,X}\left(y|s,x\right)f_{S|X}\left(s|x\right):\right.\left.f_{Y|S,X},f_{X},f_{S|X}\text{ are arbitrary density}\right\},$$

and establish the efficiency bounds for RAL estimators under ${ℳ}_{\text{comp}}$ by deriving the associated efficient influence function in the following proposition. We denote the nuisance parameters for $f_{Y|S,X},\;f_{X}$ and $f_{S|X}$ as η. We use $\eta_{0}$ to denote the true underlying nuisance parameter that generates the data. The parameter of interest $\beta_{0}$ is not part of the model ${ℳ}_{\text{comp}}$ but defined by the implicit function through the moment condition (3).

**Proposition 10** *The efficient influence function for* $\theta =x_{\text{std}}^{⊤}\beta$ *under* ${ℳ}_{\text{comp}}$ is

$$\phi_{\text{eff }}\left(D_{i};\theta_{0},\eta_{0}\right)=\frac{R_{i}}{\rho }u_{0}^{⊤}X_{i}\left\{Y_{i}-E\left(Y_{i}|S_{i},X_{i}\right)\right\}-u_{0}^{⊤}X_{i}\left\{E\left(Y_{i}|S_{i},X_{i}\right)-g\left(\beta_{0}^{⊤}X_{i}\right)\right\}.$$

Under the Assumptions of Theorem 7 and additionally $E\left(Y_{i}|S_{i},X_{i}\right)=g\left(\gamma_{0}^{⊤}W_{i}\right)$, our SAS debiased estimator admits the same influence function

$$\hat{x_{\text{std}}^{⊤}\beta }-\frac{x_{\text{new}}^{⊤}\beta_{0}}{{∥x_{\text{new}}∥}_{2}}=\frac{1}{N}\sum_{i=1}^{N}\phi_{\text{eff }}\left(D_{i};\theta_{0},\eta_{0}\right)+o_{p}\left((\rho N{)}^{-1/2}\right)$$

according to Appendix B3 Step 2 (A.31).

## 5. Simulation

We have conducted extensive simulation studies to evaluate the finite sample performance of the SAS estimation and inference procedures under various scenarios. Throughout, we let p=500, q=100, N=20000 and consider n=500. The signals in β are varied to be approximately sparse or fully dense with a mixture of strong and weak signals. The surrogates S are either moderately and strongly predictive of Y as specified below. For each configuration, we summarize the results based on 500 simulated datasets. We compare our SAS procedure with the supervised LASSO (SLASSO) that (1) estimates the $\beta_{0}$ by regressing Y to X over the labeled data with Lasso; (2) draw inference on $x_{\text{new}}^{⊤}\beta_{0}$ with the one-step debiased Lasso van de Geer et al. (2014).

To mimic the zero-inflated discrete distribution of EHR features, we first generate $Z_{i,1}^{x},\ldots ,Z_{i,p}^{x},Z_{i}^{u},Z_{i,1}^{s},\ldots ,Z_{i,q}^{s}$ independently from $N\left(0,\;25\right)$. Then we construct $X_{i}$ from $\left\{Z_{i}^{u},Z_{i}^{x}={\left(Z_{i,1}^{x},\ldots ,Z_{i,1}^{x}\right)}^{⊤}\right\}$ via the transformation $\varsigma \left(z\right)=\left\lfloor \text{log}\left\{1+\text{exp}\left(z\right)\right\}\right\rfloor :$

$$X_{i,1}=\left\{\varsigma \left(\sum_{j=2}^{p}2X_{i,j}/\sqrt{p-1}+Z_{i,1}^{x}/\sqrt{2}\right)-\mu_{x}\right\}/\sigma_{x},$$

$$X_{i,j}=\left[\varsigma \left(Z_{i,j}^{x}\sqrt{1-p^{-1}}+Z_{i}^{u}/\sqrt{p}\right)-\mu_{x}\right]/\sigma_{X},j=2,\ldots ,p.$$

We standardize $X_{i,j}$ to roughly mean zero and unit variance with $\mu_{X}=1.80$ and $\sigma_{X}=2.74$. The shared term $Z_{i}^{u}$ induces correlation among the covariates.

For S and Y, we consider two scenarios under which the imputation model is either correctly or incorrectly specified. We present the “Scenario I: neither the risk prediction model nor the imputation model is correctly specified” in the main text and the “Scenario II: The imputation model is correctly specified and exactly sparse” in Section A of the Supplementary materials.

**Scenario I: neither the risk prediction model nor the imputation model is correctly specified.** In this scenario, we first generate $Y_{i}$ from the probit model

$$P\left(Y_{i}=1|Z_{i}^{x}\right)=\Phi \left(\alpha^{⊤}Z_{i}^{x}\right)\;\text{ with }\;\Phi (x)=\int_{-\infty }^{x}(2\pi {)}^{-1/2}e^{-x^{2}/2}dx,$$

and then generate S from

$$S_{i,1}=\left\{\varsigma \left(Z_{i,1}^{s}/2+\theta Y_{i}\right)-\mu_{s}\right\}\sigma_{s}^{-1}+\xi^{⊤}X_{i},\;\text{ and }\;S_{i,j}=\left\{\varsigma \left(Z_{i,j}^{s}\right)-\mu_{x}\right\}\sigma_{x}^{-1},j=2,\ldots ,p.$$

We chose $\mu_{s}$ and $\sigma_{s}$ depending on α such that $S_{i,1}$ is roughly mean 0 and variance 1. Under this setting, a logistic imputation model would be misspecified but nevertheless approximately sparse with appropriately chosen ξ. The coefficients α control the optimal prediction accuracy of X for Y while θ controls the optimal prediction accuracy of S for Y. We consider two α of different sparsity patterns, which also determine the rest of parameters

$$\begin{array}{ll} \text{ Sparse }\left(s_{\alpha }=3\right): & \alpha ={\left(0.45,\;0.318,\;0.318,0_{497\times 1}^{⊤}\right)}^{⊤},\mu_{S}=1.82,\sigma_{S}=2.01,\\ \text{ Dense }\left(s_{\alpha }=500\right): & \alpha ={\left(0.316,\;0.059_{29\times 1}^{⊤},0.007_{470\times 1}^{⊤}\right)}^{⊤},\mu_{S}=2.71,\sigma_{S}=2.68, \end{array}$$

where $a_{k\times 1}=(a,\ldots ,a{)}_{k\times 1}^{⊤}$ for any a. The sparsity of α affects the approximate sparsity of β subsequently (Table 1), which we measured by the squared ratio between $\ell_{1}$ norm and $\ell_{2}$ norm

(33) $$𝒮(\beta )=∥\beta {∥}_{1}^{2}/∥\beta {∥}_{2}^{2},\underset{j:\beta_{j}\neq 0}{min}\left|\beta_{j}\right|\leq 𝒮(\beta )/∥\beta {∥}_{0}\leq 1.$$

We consider two θ: (a) θ=0.6 for S to be moderately predictive of Y; and (b) θ=1 for strong surrogates. The parameter ξ depends on both the choices of α and θ:

$$s_{\alpha }=3,\theta =0.6:\;\xi ={\left(0:407,\;0.330,\;0.330,\;0.005_{497\times 1}^{⊤}\right)}^{⊤},$$

$$s_{\alpha }=3,\theta =1:\;\xi ={\left(0.199,\;0.163,\;0.163,\;0.002_{497\times 1}^{⊤}\right)}^{⊤},$$

$$s_{\alpha }=500,\theta =0.6:\;\xi ={\left(0.350,0.0644_{29\times 1}^{⊤},0.011_{470\times 1}^{⊤}\right)}^{⊤},$$

$$s_{\alpha }=500,\theta =1:\;\xi ={\left(0.169,0.032_{29\times 1}^{⊤},0.005_{470\times 1}^{⊤}\right)}^{⊤}.$$

Due to the complexity of the data generating process and the noncollapsibility of the logistic regression models, we cannot analytically express the true $\beta_{0}$ in both scenarios. Instead, we numerically evaluate $\beta_{0}$ with a large simulated data using the oracle knowledge of the ex-changeability among covariates according to the model

$$\text{logit}\left\{P\left(Y_{i}=1|S_{i,1}\right)\right\}\sim \eta_{0}+\eta_{1}X_{i,1}+\eta_{2}\sum_{j=2}^{s_{\alpha }}X_{i,j}+\eta_{3}\sum_{j=s_{\alpha }+1}^{p}X_{i,j}.$$

We derive the true $\beta_{0}$ as

$$\beta_{0}={\left(\eta_{0},\eta_{1},{\left(\eta_{2}\right)}_{s_{\alpha }\times 1}^{⊤},{\left(\eta_{3}\right)}_{\left(p-s_{\alpha }\right)\times 1}^{⊤}\right)}^{⊤}\text{.}$$

We report the simulation settings under Scenario I in Table 1, where we present the predictive power of the oracle estimation and the lasso estimation. We also report the average area-under-curve (AUC) of the receiver operating characteristic (ROC) curve for oracle $\beta_{0}$, SLASSO and the proposed SAS estimation. Our SAS estimation achieves a better AUC compared to supervised LASSO across all scenarios, and is comparable to the AUC with the true coefficient $\beta_{0}$. Besides, we observe that the AUC of supervised LASSO is sensitive to the approximate sparsity $𝒮\left(\beta_{0}\right)$, while the AUC of SAS estimation does not seem to be affected by $𝒮\left(\beta_{0}\right)$.

To evaluate the SAS inference for the individualized prediction, we consider six different choices of $x_{\text{new}}$. We first select $\left\{x_{\text{new}}^{L},x_{\text{new}}^{M},x_{\text{new}}^{H}\right\}$ from a random sample of $x_{\text{new}}$ generated from the distribution of $X_{i}$ such that their predicted risks are around 0.2, 0.5, and 0.7, corresponding to low, moderate and high risk. We additionally consider three sets of $x_{\text{new}}$ with different levels of sparsity:

$$\begin{array}{ll} \text{ Sparse: } & x_{\text{new }}^{S}={\left(1,\;1,0_{499\times 1}^{⊤}\right)}^{⊤};\\ \text{ Intermediate: } & x_{\text{new }}^{I}={\left(1,0.183_{30\times 1}^{⊤},0_{470\times 1}^{⊤}\right)}^{⊤};\\ \text{ Dense: } & x_{\text{new }}^{D}={\left(1,0.045_{500\times 1}^{⊤}\right)}^{⊤}. \end{array}$$

In Table 2, we compare our SAS estimator of $x_{\text{new}}^{⊤}\beta_{0}$ with the corresponding SLASSO across all settings under Scenario I. The root mean-squared-error (rMSE) of the SAS estimation decays proportionally with the sample size, while the rMSE of the supervised LASSO provides evidence of inconsistency for moderate and dense deterministic $x_{\text{new}}$. The bias of the supervised LASSO is also significantly larger than that of the SAS estimation. The performance of the SAS estimation is insensitive to sparsity of $\beta_{0}$, while that of supervised LASSO severely deteriorate with dense $\beta_{0}$. The improvement from the supervised LASSO to the SAS estimation is regulated by the surrogate strength.

In Table 3, we compare our SAS inference with supervised debiased LASSO across the settings under Scenario I. Our SAS inference procedure attains approximately honest coverage of 95% confidence intervals for all types of $x_{\text{new}}$ under all scenarios. Unsurprisingly, the debiased SLASSO has under coverage for the deterministic $x_{\text{new}}$ as the consequence of violation to the sparsity assumption for $\beta_{0}$ and precision matrix. Under our design, the first covariate $X_{1}$ has the strongest dependence upon the other covariates, whose associated row in the precision matrix is thus densest. Consequently, the inference for $\beta^{⊤}x_{\text{new}}^{S}=\beta_{0}+\beta_{1}$ The debiased SLASSO also has an acceptable coverage for random $x_{\text{new}}^{L},x_{\text{new}}^{M},x_{\text{new}}^{H}$ sampled from the covariate distribution despite the presence of substantial bias, which we attribute to the even larger variance that dominates the bias. In contrast, our SAS inference has small bias across all scenarios and improved variance from the strong surrogate.

According to Tables A1, A2 and A3 in the Appendix A, the results under Scenario II are consistent with our findings under Scenario I. We also compares SAS to an unsupervised learning approach using proxy outcome derived from surrogates in the Appendix A. Under the Scenario III very similar to Scenario I, SAS performs well as in Scenario I while the unsupervised learning approach fails completely. This is expected since the unsupervised approach requires that the deviation of the surrogates from the true outcome S-Y is uncorrelated with the risk factors X. Otherwise, spurious association between outcome Y and risk factors X can be induced, creating bias in estimation of risk prediction model.

## 6. Application of SAS to EHR Study

We applied the proposed SAS method to the risk prediction of Type II Diabetes Mellitus (T2DM) using EHR and genomic data of participants of the Mass General Brigham Biobank study. Number of genetic risk factors among single nucleotide polymorphism for T2DM has grown exponentially following the expansion of genome-wide association studies. As an incomplete summary, Voight et al. (2010),Morris et al. (2012) and Scott et al. (2017) each discovered around a dozen new risk SNPs for T2DM, and the recent studies by Mahajan et al. (2018) and Vujkovic et al. (2020) discovered 135 and 558 new risk SNPs, respectively. Some new risk SNPs in Mahajan et al. (2018) even had large coefficients in the poly genetic risk score. The ever growing number of risk SNPs suggest that the genetic risk prediction model for T2DM may be dense. Compared to the large biobank data that generated the genome-wide association studies, EHR captures the temporal information of T2DM onset and other phenotypes predictive for T2DM and thus may provide a more accurate forecasting for T2DM. As we mentioned in the introduction, direct extraction of disease onset from EHR by diagnosis code or mention in medical notes may contain substantial false positives. From an expert annotation of the medical histories for 271 patients, we found 38 patients with T2DM diagnosis code and 161 patients with mention of T2DM in medical notes who actually had never developed T2DM. The annotation process requires intensive labor of highly skilled medical experts, leading to the limited number of labels.

To define the study cohort, we extracted from the EHR of each patient their date of first EHR encounter $\left(t_{ini}\right)$, follow up period (C), the counts and dates for the diagnosis codes and note mentions of clinical concepts related to T2DM as well as its risk factors. We only included patients who do not have any diagnosis code or note mention of T2DM up to baseline, where the baseline time is defined as 1990 if $t_{ini}$ is prior to 1990 and as their first year if $t_{ini}\geq 1990$. Although neither the diagnosis code nor note mention of T2DM is sufficiently specific, they are highly sensitive and can be used to accurately remove patients who have already developed T2DM at baseline. This exclusion criterion resulted in N=20216 patients who are free of T2DM at baseline and have both EHR and genomics features for risk modeling. Among those, we have a total of n=271 patients whose T2DM status during follow up, Y, has been obtained via manual chart review. The prevalence of T2DM was about 14% based on labeled data.

We aim to develop a risk prediction model for Y by fitting a working model $P(Y=1|X)=g\left(\beta_{0}^{⊤}X\right)$, where the baseline covariate vector X includes age, gender, indicator for occurrence of diagnosis code and note counts for obesity, hypertension, coronary artery disease (CAD), hyperlipidemia during the first year window, as well as a total of 49 single nucleotide polymorphism previously reported as associated with T2DM in Mahajan et al. (2018) with odds ratio greater than 1.1. We additionally adjust for follow up by including log⁡(C) and allow for non-linear effects by including two-way interactions between the SNPs and other baseline covariates. All variables with less than 10 nonzero values within the labelled set are removed, resulting the final covariates to be of dimension p=260. We standardize the covariates to have mean 0 and variance 1. To impute the outcome, we used the predicted probability of T2DM derived from the unsupervised phenotyping method MAP (Liao et al., 2019), which achieves an AUC of 0.98, indicating a strong surrogate. In addition to the proposed SAS procedure, we derive risk prediction models based on the supervised LASSO with both the same set of covariates. We let K=5 in cross-fitting and use 5-fold cross-validation for tuning parameter selection. To compare the performance of different risk prediction models, we use 10-fold cross-validation to estimate the out-of-sample AUC. We repeated the process 10 times and took average of predicted probabilities across the repeats for each labelled sample and method in comparison.

**Figure 2:**
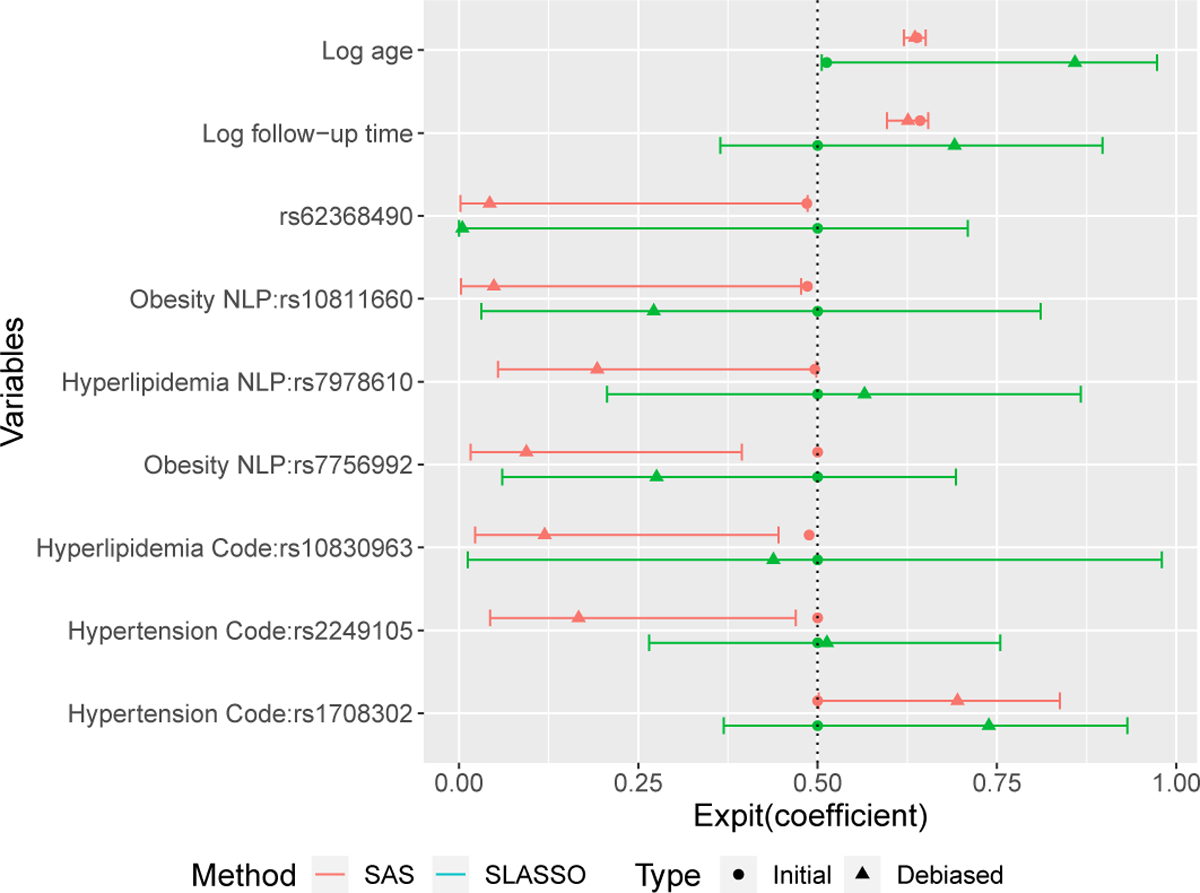
Point and 95% confidence interval estimates for the coefficients with nominal p-value < 0.05 from SAS inference. The horizontal bars indicate the estimated 95% confidence intervals. The solid points indicate the (initial) estimates, and the triangles indicate debiased estimates. Colors red and green indicate different methods, SAS and SLASSO, respectively.

In Figure 2, we present the estimated β coefficients for the covariates that received p-value less than 0.05 from the SAS inference. The confidence intervals are generally narrower from the SAS inference. For the coefficients of baseline age and follow-up time, the SAS inference produced much narrower confidence interval than debiased SLASSO, which are expected to have a positive effect on the T2DM onset status during the observation. In addition, the SAS inference identified one global genetic risk factor and 6 other subgroup genetic risk factors while SLASSO identified none of these.

In Table 4, we present the AUCs of the estimated risk prediction models using the high dimensional X. It is important to note that AUC is a measurement of prediction accuracy, so debiasing might lead to worse AUC by accepting larger variability for reduced bias. The AUC from SLASSO is very poor, probably due to the over-fitting bias with the small sample sizes of the labeled set. With the information from a large unlabeled data, SAS produced the significantly higher AUC than the SLASSO.

**Figure 3:**
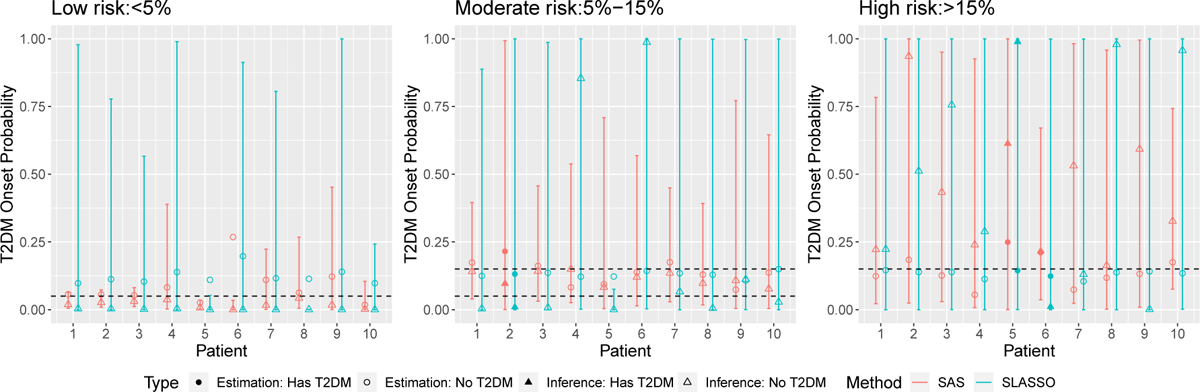
Point and 95% confidence interval estimates for the predicted risks of 30 randomly selected patients. The vertical bars indicate the estimated 95% confidence intervals. The circle and the triangle shapes correspond to (initial) estimation and debiased estimation, correspondingly. Solid points indicate the observed T2DM cases. Colors red and green indicate different methods, SAS and SLASSO.

For illustration, we present in Figure 3 the individual risk predictions with 95% confidence intervals for three sets of 10 patients with each set randomly selected from low (< 5%), medium (5% ~ 15%) or high risk (> 15%) subgroups. These risk groups are constructed for illustration purposes and a patient with $x_{\text{new}}$ classified to low, medium and high risk if $expit\left({\hat{\beta }}^{⊤}x_{\text{new}}\right)$ belongs to the low, medium and high tertiles of $\left\{expit\left({\hat{\beta }}^{⊤}X_{i}\right),i=1,\ldots ,N\right\}$. We observe that the confidence intervals for patients with predicted The debiased SLASSO inference is not very informative with most error bars stretching from zero to one. The contrast between SAS CIs and SLASSO CIs demonstrates the improved efficiency as the result of leveraging information from the unlabeled data through predictive surrogates.

## 7. Discussion

We proposed the SAS estimation and inference method for high-dimensional risk prediction model with diminishing proportion of observed outcomes. With a sparse imputation model based on predictive surrogates, the SAS can recover a dense risk prediction model impossible to learn from supervised method, as well as achieve better efficiency than supervised method when the latter is applicable. We show that the theoretical advantages lead to better prediction accuracy and shorter confidence intervals in simulations and real data example.

While the SAS procedure is a powerful tool with minimal requirements, caution should be given to the inclusion of highly informative surrogates so that the imputation model is sparse (or approximately sparse). If all surrogates poorly predicts Y with a dense imputation model, the SAS procedure can lead to a compromised convergence rate in estimation. While the current study is motivated by the existence of easy-to-learn imputation model with highly predictive surrogates, the SAS framework can be extended to settings where the imputation model is not easier to learn than the model for Y∣X. When the imputation model is estimable but more dense than the risk prediction model (i.e. $s_{\beta }<s_{\gamma }$), we can following similar strategies as in our SAS inference procedure to reduce the bias incurred during the imputation step from $\hat{\gamma }$. Specifically, we may consider a debiased estimator for $\hat{\beta }$

$${\hat{\beta }}_{\text{debias }}=\underset{\beta \in R^{p+1}}{argmin}\sum_{k=1}^{K}\left[\frac{1}{N}\sum_{i\in {𝒥}_{k}}\ell \left(g\left({\hat{\gamma }}^{(k)T}W_{i}\right),\beta^{⊤}X_{i}\right)+\frac{1}{n}\sum_{i\in {ℐ}_{k}}\beta^{⊤}X_{i}\left\{g\left({\hat{\gamma }}^{(k)T}W_{i}\right)-Y_{i}\right\}\right]+\lambda {∥\beta_{-1}∥}_{1}.$$

This debiased SAS estimation will attain the optimal rate $\sqrt{s_{\beta }\text{log}(p)/n}$ and we also expect an efficiency gain in the resulting variance compared to the supervised estimator, in analog to the efficiency gain observed in SAS inference. Adaptive approaches to infer whether a given dataset falls into the setting with $s_{\beta }>s_{\gamma }$ or $s_{\beta }<s_{\gamma }$ is straightforward in simpler settings when $s_{\beta }$ and $s_{\gamma }$ can be estimated but warrants future research in general. In the extremely dense imputation model setting when $s_{\gamma }>n$, information theoretical bound has indicated that the imputation model will be inestimable, invalidating any subsequent steps involving $\hat{\gamma }$. A possible solution is to redefine the imputation model as the sparser model between the risk prediction model and the original imputation model. A potential approach to identifying such a sparser imputation model is through the under-identified Dantzig Selector

$${\hat{\gamma }}_{ada}=\underset{\gamma \in R^{p+q}}{argmin}∥\gamma {∥}_{1},\;\text{Subject to }{∥\frac{1}{n}\sum_{i=1}^{n}X_{i}\left\{Y_{i}-g\left(\gamma^{⊤}W_{i}\right)\right\}∥}_{\infty }\leq \lambda .$$

Both $\gamma_{0}$ and ${\left(\beta_{0}^{⊤},0_{q}^{⊤}\right)}^{⊤}$ should fall in the feasible region with suitable λ, and the minimization over $L_{1}$ norm may pick the sparsest element from the feasible class. Using ${\hat{\gamma }}_{ada}$ in SAS estimation may attain uniform optimal rate for any $s_{\beta }$ and $s_{\gamma }$. Theoretical studies of the above proposals warrant future research.

## Supplementary Material

1

## Figures and Tables

**Figure 1:**
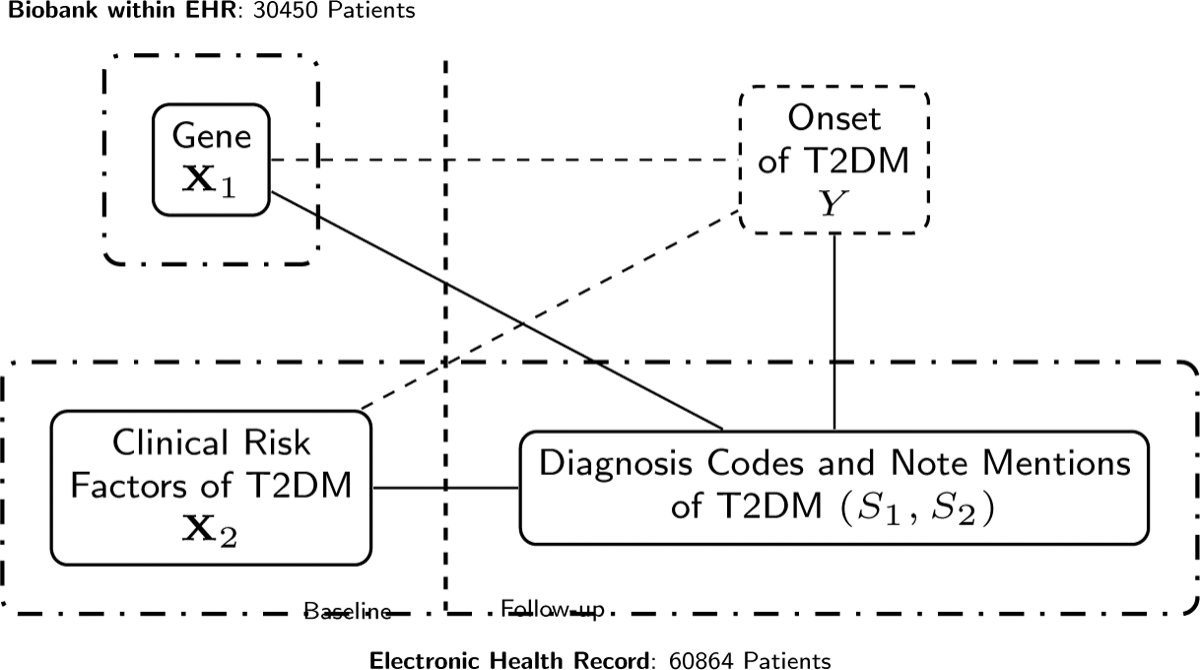
A dense prediction model (graph with dashed lines) can be compress to a sparse imputation model (through graph with solid lines) when the effect of most baseline covariates are reflected in a few variables in the EHR monitoring the development of the event of interest.

**Table 1:** AUC Table for simulations with 500 labels under Scenario I. The AUCs are evaluated on an independent testing set of size 100. We approximately measure the sparsity by $𝒮\left(v\right)=∥v{∥}_{1}^{2}/∥v{∥}_{2}^{2}$.

Scenario			Prediction Accuracy (AUC)		
Surrogate	$𝒮\left(\beta_{0}\right)$	$𝒮\left(\gamma_{0}\right)$	Oracle	SLASSO	SAS
Strong	174	1.32	0.724	0.660	0.711
Moderate	174	1.26	0.724	0.660	0.713
Strong	28.3	1.33	0.719	0.694	0.713
Moderate	28.3	1.24	0.719	0.694	0.711

**Table 2:** Comparison of SAS Estimation to the supervised LASSO (SLASSO) with Bias, Empirical standard error (ESE) and root mean-squared error (rMSE) of the linear predictions $x_{\text{new}}^{⊤}\beta_{0}$ under Scenario I 500 labels, moderate or large $𝒮\left(\beta_{0}\right)$ and strong or moderate surrogates.

	SLASSO			SAS: Moderate			SAS: Strong		
Type	Bias	ESE	rMSE	Bias	ESE	rMSE	Bias	ESE	rMSE
Moderate $𝒮\left(\beta_{0}\right)$									
$x_{\text{new }}^{⌞}$	0.605	0.387	0.719	0.165	0.249	0.298	0.118	0.196	0.229
$x_{\text{new }}^{M}$	−0.083	0.337	0.347	−0.008	0.246	0.246	−0.016	0.195	0.196
$x_{\text{new }}^{H}$	−0.718	0.521	0.887	−0.234	0.294	0.376	−0.176	0.225	0.286
$x_{\text{new }}^{\text{S}}$	−0.072	0.144	0.161	−0.080	0.094	0.123	−0.018	0.078	0.080
$x_{\text{new }}^{\text{I}}$	−0.460	0.096	0.470	−0.110	0.093	0.143	−0.055	0.071	0.090
$x_{\text{new }}^{\text{D}}$	−0.413	0.091	0.423	−0.110	0.089	0.141	−0.114	0.069	0.133
Large $𝒮\left(\beta_{0}\right)$									
$x_{\text{new }}^{L}$	0.389	0.275	0.477	0.161	0.215	0.269	0.133	0.264	0.296
$x_{\text{new }}^{M}$	−0.017	0.280	0.280	−0.014	0.213	0.213	−0.017	0.268	0.268
$x_{\text{new }}^{H}$	−0.600	0.481	0.769	−0.251	0.271	0.370	−0.164	0.296	0.339
$x_{\text{new }}^{S}$	−0.202	0.140	0.246	−0.074	0.097	0.122	−0.009	0.078	0.079
$x_{\text{new}}^{\text{I}}$	−0.178	0.098	0.203	−0.075	0.086	0.115	−0.071	0.075	0.103
$x_{\text{new }}^{\text{D}}$	−0.185	0.090	0.206	−0.109	0.084	0.138	−0.113	0.073	0.135

**Table 3:** Bias, Empirical standard error (ESE), average of the estimated standard error (ASE) along with empirical coverage of the 95% confidence intervals (CP) for the debiased supervised LASSO (SLASSO) and debiased SAS estimator of linear predictions $x_{\text{new}}^{⊤}\beta_{0}$ under Scenario I with 500 labels, moderate or large $𝒮\left(\beta_{0}\right)$ and strong or moderate surrogates.

					Debiased SAS							
	Debiased SLASSO				Moderate Surrogates				Strong Surrogates			
Type	Bias	ESE	ASE	CP	Bias	ESE	ASE	CP	Bias	ESE	ASE	CP
Risk prediction model approximatedly sparse												
$x_{\text{new}}^{⌞}$	−0.290	1.901	1.896	0.948	0.021	1.873	1.864	0.949	0.018	1.531	1.531	0.950
$x_{\text{new}}^{M}$	−0.091	1.994	1.981	0.947	−0.007	1.961	1.954	0.950	−0.015	1.560	1.570	0.953
$x_{\text{new}}^{H}$	0.348	2.106	2.074	0.942	−0.050	2.036	2.039	0.950	−0.011	1.632	1.623	0.950
$x_{\text{new}}^{\text{S}}$	0.171	0.157	0.128	0.694	−0.019	0.149	0.150	0.950	−0.001	0.132	0.125	0.924
$x_{\text{new}}^{\text{I}}$	−0.001	0.129	0.125	0.938	−0.013	0.123	0.116	0.932	0.010	0.101	0.094	0.920
$x_{\text{new}}^{\text{D}}$	0.141	0.137	0.138	0.812	−0.011	0.123	0.118	0.944	−0.001	0.096	0.095	0.940
Large $𝒮\left(\beta_{0}\right)$												
$x_{\text{new}}^{\text{L}}$	−0.134	1.918	1.914	0.951	0.018	1.875	1.878	0.951	0.018	1.529	1.524	0.948
$x_{\text{new}}^{M}$	−0.056	1.970	1.962	0.948	−0.020	1.911	1.927	0.952	0.005	1.603	1.597	0.950
$x_{\text{new}}^{H}$	0.109	2.051	2.029	0.945	−0.022	1.997	1.991	0.950	−0.040	1.671	1.668	0.951
$x_{\text{new}}^{\text{S}}$	0.029	0.155	0.127	0.892	−0.008	0.153	0.147	0.946	−0.013	0.133	0.131	0.938
$x_{\text{new}}^{\text{I}}$	0.002	0.131	0.125	0.930	0.001	0.122	0.114	0.936	0.002	0.101	0.098	0.936
$x_{\text{new}}^{\text{D}}$	0.113	0.135	0.139	0.874	−0.007	0.119	0.116	0.938	−0.003	0.099	0.097	0.960

**Table 4:** The cross-validated (CV) AUC the estimated risk prediction models with high dimensional EHR and genetic features based on SAS and supervised LASSO. Shown also are the AUC of the imputation model derived for the SAS procedure.

Method	Imputation	SAS	SLASSO
CV AUC	0.928	0.763	0.488
