# Supplementary material for: Surrogate Assisted Semi-supervised Inference for High Dimensional Risk Prediction
Source: J Mach Learn Res. Author manuscript; Available in PMC 2024 Mar 18. (PMC10947223)
Supplement: 1 [file NIHMS1971733-supplement-1.pdf]

Supplementary Material

We present the simulation Scenario II in which the imputation model is correctly specified and exactly sparse in Appendix A. We also compared the SAS estimation and inference with unsupervised regression that uses only $\mathbf{S}$ to derive a proxy outcome in the simulation Scenario III very similar to Scenario I. The proofs of Theorems 1, 7, Corollary 5 and Propositions 9 and 10 are given in Appendix B. The technical details are put in Appendix C. Definitions and existing results are stated in Appendix D.

Appendix A. Additional Simulation

Scenario II: The imputation model is correctly specified and exactly sparse. In the second scenario, we first generate $\mathbf{S}_i$ from

$$S_{i,1} = \left[\varsigma\{\nu Z^s_{i,1} + \boldsymbol{\alpha}^\mathsf{T}(\mathbf{Z}^x_i\sqrt{1-p^{-1}} + Z^u_i/\sqrt{p})\} - \mu_\mathbf{S}\right]/\sigma_\mathbf{S}.$$

and $S_{i,j} = \{\varsigma(Z^s_{i,j}) - \mu_\mathbf{X}\}/\sigma_\mathbf{X}$ for $j = 2, \ldots, p$, and then generate Y_i from a sparse model

$$\mathbb{P}(Y_i = 1|\mathbf{X}_i) = \text{expit}(\theta S_{i,1}).$$

We chose $\mu_\mathbf{S} \approx 0.66$ and $\sigma_\mathbf{S} \approx 1$ such that $S_{i,1}$ is roughly mean 0 and variance 1. Under this setting, the imputation model holds with $s_\gamma = 1$. The factor ν and the coefficients $\boldsymbol{\alpha}$ control the predictiveness of $\mathbf{X}$ for S_1 and Y while θ controls the predictiveness of $\mathbf{W}$ for Y. We consider two $\boldsymbol{\alpha}$ of different sparsity patterns,

$$\text{Sparse } (s_{\boldsymbol{\alpha}} = 3): \quad \boldsymbol{\alpha} = (0.3, 0.212, 0.212, \mathbf{0}^\mathsf{T}_{497\times1})^\mathsf{T}$$
$$\text{Dense } (s_{\boldsymbol{\alpha}} = 500): \quad \boldsymbol{\alpha} = (0.211, \mathbf{0.039}^\mathsf{T}_{29\times1}, \mathbf{0.004}^\mathsf{T}_{470\times1})^\mathsf{T},$$

where $\mathbf{a}_{k\times1} = (a, ..., a)^\mathsf{T}_{k\times1}$ for any a. Similar to Scenario I, the sparsity of $\boldsymbol{\alpha}$ regulates the approximate sparsity of $\boldsymbol{\beta}$ measured by (33) (See Table 1). We consider two sets of (ν, θ) to allow $\mathbf{W}$ to be either moderately or strongly predictive of Y:

$$\text{Moderate: } \nu = 0.4,\ \theta = 2; \qquad \text{and} \qquad \text{Strong: } \nu = 0.6,\ \theta = 3.7.$$

The layouts of Tables A2 and A3 are different from those of 2 and Table 3 because of the different data generating mechanism. The distribution of $Y_i \mid \mathbf{X}_i$ is not affected by the distribution of $\mathbf{S}_i$ in Scenario I, while the property does not hold in Scenario II.

Table A1: AUC Table for simulations with 500 labels under Scenario II. The AUCs are evaluated on an independent testing set of size 100. We approximately measure the sparsity by $\mathcal{S}(\mathbf{v}) = \|\mathbf{v}\|_1^2/\|\mathbf{v}\|_2^2$.

Scenario			Prediction Accuracy (AUC)		
Surrogate	$\mathcal{S}(\boldsymbol{\beta}_0)$	$\mathcal{S}(\boldsymbol{\gamma}_0)$	Oracle	SLASSO	SAS
Strong	159	1.10	0.715	0.660	0.702
Moderate	128	1.06	0.715	0.665	0.704
Strong	26.4	1.09	0.710	0.691	0.708
Moderate	18.4	1.03	0.709	0.693	0.707

Table A2: Comparison of SAS Estimation to the supervised LASSO (SLASSO) with Bias, Empirical standard error (ESE) and root mean-squared error (rMSE) of the linear predictions $\mathbf{x}_{\text{new}}^{\mathsf{T}}\boldsymbol{\beta}_0$ under Scenario II with 500 labels, moderate or large $\mathcal{S}(\boldsymbol{\beta}_0)$ and strong or moderate surrogates.

	Moderate Surrogates						Strong Surrogates					
	SLASSO			SAS			SLASSO			SAS		
Type	Bias	ESE	rMSE	Bias	ESE	rMSE	Bias	ESE	rMSE	Bias	ESE	rMSE
Risk prediction model approximately sparse												
$\mathbf{x}_{\text{new}}^{\mathsf{L}}$	0.505	0.378	0.631	0.163	0.283	0.327	0.349	0.278	0.446	0.085	0.222	0.238
$\mathbf{x}_{\text{new}}^{\mathsf{M}}$	-0.140	0.331	0.359	-0.047	0.272	0.276	-0.113	0.282	0.304	-0.058	0.217	0.225
$\mathbf{x}_{\text{new}}^{\mathsf{H}}$	-0.713	0.512	0.878	-0.262	0.313	0.408	-0.678	0.469	0.825	-0.210	0.241	0.320
$\mathbf{x}_{\text{new}}^{\mathsf{S}}$	-0.111	0.143	0.181	-0.058	0.081	0.100	-0.190	0.142	0.237	-0.037	0.063	0.072
$\mathbf{x}_{\text{new}}^{\mathsf{I}}$	-0.437	0.098	0.448	-0.119	0.076	0.141	-0.155	0.098	0.183	-0.065	0.061	0.089
$\mathbf{x}_{\text{new}}^{\mathsf{D}}$	-0.349	0.093	0.361	-0.138	0.078	0.158	-0.150	0.093	0.176	-0.112	0.063	0.129
Large $\mathcal{S}(\boldsymbol{\beta}_0)$												
$\mathbf{x}_{\text{new}}^{\mathsf{L}}$	0.366	0.266	0.453	0.142	0.224	0.265	0.482	0.398	0.625	0.117	0.300	0.322
$\mathbf{x}_{\text{new}}^{\mathsf{M}}$	-0.060	0.275	0.282	-0.035	0.213	0.216	-0.199	0.337	0.391	-0.082	0.299	0.310
$\mathbf{x}_{\text{new}}^{\mathsf{H}}$	-0.656	0.475	0.810	-0.272	0.257	0.374	-0.749	0.503	0.903	-0.214	0.325	0.389
$\mathbf{x}_{\text{new}}^{\mathsf{S}}$	-0.236	0.139	0.274	-0.087	0.079	0.117	-0.054	0.138	0.148	0.003	0.063	0.063
$\mathbf{x}_{\text{new}}^{\mathsf{I}}$	-0.173	0.096	0.197	-0.078	0.077	0.109	-0.409	0.097	0.420	-0.094	0.057	0.110
$\mathbf{x}_{\text{new}}^{\mathsf{D}}$	-0.144	0.092	0.171	-0.101	0.080	0.129	-0.359	0.093	0.371	-0.154	0.060	0.166

Scenario III: Similar to Scenario I. In the third scenario, we repeat the data generation process of Scenario I except for difference values for $\boldsymbol{\xi}$:

$$
\begin{aligned}
s_{\boldsymbol{\alpha}} = 3,\, \theta = 0.6: &\quad \boldsymbol{\xi} = (-0.593, 0.330, 0.330, \mathbf{0.005}_{497\times 1}^{\mathsf{T}})^{\mathsf{T}},\\
s_{\boldsymbol{\alpha}} = 3,\, \theta = 1: &\quad \boldsymbol{\xi} = (-0.801, 0.163, 0.163, \mathbf{0.002}_{497\times 1}^{\mathsf{T}})^{\mathsf{T}},\\
s_{\boldsymbol{\alpha}} = 500,\, \theta = 0.6: &\quad \boldsymbol{\xi} = (-0.650, \mathbf{0.064}_{29\times 1}^{\mathsf{T}}, \mathbf{0.011}_{470\times 1}^{\mathsf{T}})^{\mathsf{T}},\\
s_{\boldsymbol{\alpha}} = 500,\, \theta = 1: &\quad \boldsymbol{\xi} = (-0.831, \mathbf{0.032}_{29\times 1}^{\mathsf{T}}, \mathbf{0.005}_{470\times 1}^{\mathsf{T}})^{\mathsf{T}}.
\end{aligned}
$$

We focus on the comparison between SAS estimation and inference and an unsupervised learning (UL) approach. For the UL approach, a proxy outcome $\widetilde{Y}$ is derived directly from the dichotomized informative surrogate S_1

$$\widetilde{Y} = \mathbb{I}(S_1 \geq s_*),$$

where the threshold s_* is chose in order to match the prevalence $\mathbb{E}(\widetilde{Y}) \approx \mathbb{E}(Y)$. Then, the UL estimation of $\boldsymbol{\beta}$ is obtained by regression $\widetilde{Y}$ to $\mathbf{X}$ under the logistic regression model over all N observations. Classical inference is used for construction of UL confidence intervals.

The layouts of Tables A5 and A6 are different from those of 2 and Table 3 because of the different benchmark method. The supervised learning methods SLASSO and Debiased SLASSO are not affected by the distribution of $\mathbf{S}_i$ in Scenario I, while UL considered in Scenario III uses $\mathbf{S}_i$ to construct its proxy outcome. UL obviously failed with ineffective classification (AUC $<$ 0.50 in Table A4), large bias in Table A5 and severe under-covering confidence intervals in Table A6. The performance of SAS is solid as in Scenario I.

Table A3: Bias, Empirical standard error (ESE) along with empirical coverage of the 95% confidence intervals (CP) for the debiased supervised LASSO (SLASSO) and debiased SAS estimator of linear predictions $\mathbf{x}_{\text{new}}^{\mathsf{T}}\boldsymbol{\beta}_0$ under Scenario II with 500 labels, moderate or large $\mathcal{S}(\boldsymbol{\beta}_0)$ and strong or moderate surrogates.

	Debiased SLASSO				Debiased SAS			
Type	Bias	ESE	ASE	CP	Bias	ESE	ASE	CP
Risk prediction model approximatedly sparse, moderate surrogates								
$\mathbf{x}_{\text{new}}^{\mathsf{L}}$	-0.236	1.936	1.915	0.947	0.014	1.786	1.771	0.950
$\mathbf{x}_{\text{new}}^{\mathsf{M}}$	-0.044	2.031	1.997	0.944	-0.028	1.873	1.853	0.947
$\mathbf{x}_{\text{new}}^{\mathsf{H}}$	0.364	2.110	2.084	0.944	-0.045	1.943	1.924	0.947
$\mathbf{x}_{\text{new}}^{\mathsf{S}}$	0.133	0.156	0.127	0.784	-0.028	0.133	0.130	0.944
$\mathbf{x}_{\text{new}}^{\mathsf{I}}$	0.004	0.124	0.126	0.942	-0.014	0.102	0.100	0.936
$\mathbf{x}_{\text{new}}^{\mathsf{D}}$	0.149	0.121	0.139	0.848	-0.014	0.104	0.105	0.948
Risk prediction model approximatedly sparse, strong surrogates								
$\mathbf{x}_{\text{new}}^{\mathsf{L}}$	-0.070	1.953	1.935	0.947	0.021	1.371	1.366	0.949
$\mathbf{x}_{\text{new}}^{\mathsf{M}}$	-0.031	2.019	1.986	0.946	-0.026	1.408	1.401	0.949
$\mathbf{x}_{\text{new}}^{\mathsf{H}}$	0.148	2.073	2.055	0.948	-0.010	1.458	1.444	0.949
$\mathbf{x}_{\text{new}}^{\mathsf{S}}$	0.029	0.153	0.127	0.894	-0.016	0.103	0.096	0.928
$\mathbf{x}_{\text{new}}^{\mathsf{I}}$	0.018	0.134	0.126	0.938	-0.004	0.081	0.079	0.944
$\mathbf{x}_{\text{new}}^{\mathsf{D}}$	0.134	0.128	0.141	0.842	-0.007	0.081	0.083	0.956
Large $\mathcal{S}(\boldsymbol{\beta}_0)$, moderate surrogates								
$\mathbf{x}_{\text{new}}^{\mathsf{L}}$	-0.092	1.942	1.925	0.950	0.004	1.796	1.792	0.951
$\mathbf{x}_{\text{new}}^{\mathsf{M}}$	-0.034	1.995	1.969	0.947	-0.018	1.852	1.835	0.951
$\mathbf{x}_{\text{new}}^{\mathsf{H}}$	0.082	2.061	2.036	0.946	-0.027	1.912	1.890	0.948
$\mathbf{x}_{\text{new}}^{\mathsf{S}}$	-0.009	0.155	0.125	0.876	-0.027	0.131	0.125	0.922
$\mathbf{x}_{\text{new}}^{\mathsf{I}}$	0.000	0.126	0.125	0.952	-0.009	0.104	0.103	0.950
$\mathbf{x}_{\text{new}}^{\mathsf{D}}$	0.119	0.126	0.139	0.894	-0.012	0.108	0.108	0.940
Large $\mathcal{S}(\boldsymbol{\beta}_0)$, strong surrogates								
$\mathbf{x}_{\text{new}}^{\mathsf{L}}$	-0.221	1.929	1.926	0.949	0.022	1.353	1.349	0.951
$\mathbf{x}_{\text{new}}^{\mathsf{M}}$	0.032	2.047	2.017	0.947	-0.003	1.427	1.414	0.950
$\mathbf{x}_{\text{new}}^{\mathsf{H}}$	0.442	2.137	2.104	0.940	-0.039	1.479	1.469	0.951
$\mathbf{x}_{\text{new}}^{\mathsf{S}}$	0.176	0.150	0.128	0.698	-0.018	0.094	0.099	0.946
$\mathbf{x}_{\text{new}}^{\mathsf{I}}$	0.030	0.128	0.129	0.936	-0.002	0.079	0.077	0.952
$\mathbf{x}_{\text{new}}^{\mathsf{D}}$	0.167	0.125	0.142	0.804	-0.008	0.082	0.080	0.954

Table A4: AUC Table for simulations with 500 labels under Scenario III. The AUCs are evaluated on an independent testing set of size 100. We approximately measure the sparsity by $S(\mathbf{v}) = \|\mathbf{v}\|_1/\|\mathbf{v}\|_2$.

Scenario			Prediction Accuracy (AUC)		
Surrogate	$\mathcal{S}(\boldsymbol{\beta}_0)$	$\mathcal{S}(\boldsymbol{\gamma}_0)$	Oracle	UL	SAS
Strong	174.08	2.31	0.72	0.43	0.69
Moderate	174.08	2.25	0.72	0.46	0.69
Strong	28.27	2.32	0.72	0.45	0.69
Moderate	28.27	2.24	0.72	0.47	0.69

Table A5: Bias, Empirical standard error (ESE) and mean-squared error (MSE) for the unsupervised learning (UL) and SAS estimator of linear predictions $\mathbf{x}_{\text{new}}^{\mathsf{T}}\boldsymbol{\beta}_0$ with under Scenario III 500 labels, approximately sparse or dense $\boldsymbol{\beta}_0$ and strong or moderate surrogates.

	UL			SAS		
Type	Bias	ESE	MSE	Bias	ESE	MSE
Moderate $\mathcal{S}(\boldsymbol{\beta}_0)$, moderate surrogates						
$\mathbf{x}_{\text{new}}^{\mathsf{L}}$	0.42	0.92	1.01	0.02	0.42	0.42
$\mathbf{x}_{\text{new}}^{\mathsf{M}}$	0.20	2.31	2.32	-0.03	0.51	0.51
$\mathbf{x}_{\text{new}}^{\mathsf{H}}$	-3.39	3.90	5.16	0.09	0.59	0.60
$\mathbf{x}_{\text{new}}^{\mathsf{S}}$	-3.73	0.07	3.73	-0.09	0.14	0.17
$\mathbf{x}_{\text{new}}^{\mathsf{I}}$	-0.42	0.14	0.44	-0.19	0.10	0.22
$\mathbf{x}_{\text{new}}^{\mathsf{D}}$	-0.03	0.15	0.16	-0.18	0.09	0.20
Large $\mathcal{S}(\boldsymbol{\beta}_0)$,moderate surrogates						
$\mathbf{x}_{\text{new}}^{\mathsf{L}}$	0.61	0.70	0.93	0.04	0.37	0.37
$\mathbf{x}_{\text{new}}^{\mathsf{M}}$	-0.04	2.54	2.54	-0.03	0.49	0.49
$\mathbf{x}_{\text{new}}^{\mathsf{H}}$	-2.53	4.66	5.30	0.07	0.59	0.59
$\mathbf{x}_{\text{new}}^{\mathsf{S}}$	-3.56	0.11	3.56	-0.14	0.14	0.19
$\mathbf{x}_{\text{new}}^{\mathsf{I}}$	-0.70	0.14	0.72	-0.11	0.09	0.15
$\mathbf{x}_{\text{new}}^{\mathsf{D}}$	-0.30	0.13	0.33	-0.12	0.09	0.15
Moderate $\mathcal{S}(\boldsymbol{\beta}_0)$, strong surrogates						
$\mathbf{x}_{\text{new}}^{\mathsf{L}}$	0.89	0.67	1.11	-0.05	0.38	0.38
$\mathbf{x}_{\text{new}}^{\mathsf{M}}$	-0.15	2.51	2.51	-0.04	0.54	0.54
$\mathbf{x}_{\text{new}}^{\mathsf{H}}$	-3.28	4.57	5.62	0.22	0.60	0.64
$\mathbf{x}_{\text{new}}^{\mathsf{S}}$	-3.83	0.07	3.83	-0.09	0.13	0.15
$\mathbf{x}_{\text{new}}^{\mathsf{I}}$	-0.99	0.12	1.00	-0.08	0.08	0.11
$\mathbf{x}_{\text{new}}^{\mathsf{D}}$	-0.57	0.12	0.58	-0.10	0.08	0.13
Large $\mathcal{S}(\boldsymbol{\beta}_0)$, strong surrogates						
$\mathbf{x}_{\text{new}}^{\mathsf{L}}$	0.76	0.82	1.12	-0.09	0.45	0.46
$\mathbf{x}_{\text{new}}^{\mathsf{M}}$	0.09	2.35	2.35	-0.03	0.57	0.58
$\mathbf{x}_{\text{new}}^{\mathsf{H}}$	-4.10	3.97	5.71	0.23	0.63	0.67
$\mathbf{x}_{\text{new}}^{\mathsf{S}}$	-3.99	0.09	3.99	-0.05	0.13	0.14
$\mathbf{x}_{\text{new}}^{\mathsf{I}}$	-0.82	0.12	0.83	-0.12	0.08	0.15
$\mathbf{x}_{\text{new}}^{\mathsf{D}}$	-0.37	0.14	0.40	-0.13	0.08	0.15

Table A6: Bias, Empirical standard error (ESE), average of the estimated standard error (ASE) along with empirical coverage of the 95% confidence intervals (CP) for the unsupervised learning (UL) and debiased SAS estimator of linear predictions $\mathbf{x}_{\text{new}}^{\mathsf{T}}\boldsymbol{\beta}_0$ under Scenario III with 500 labels, approximately sparse or dense $\boldsymbol{\beta}_0$ and strong or moderate surrogates.

	UL				SAS			
Type	Bias	ESE	ASE	CP	Bias	ESE	ASE	CP
Moderate $\mathcal{S}(\boldsymbol{\beta}_0)$, moderate surrogates								
$\mathbf{x}_{\text{new}}^{\mathsf{L}}$	0.42	0.92	0.40	0.53	-0.27	1.95	1.92	0.94
$\mathbf{x}_{\text{new}}^{\mathsf{M}}$	0.20	2.31	0.42	0.26	-0.04	2.09	2.03	0.94
$\mathbf{x}_{\text{new}}^{\mathsf{H}}$	-3.39	3.90	0.45	0.12	0.43	2.18	2.14	0.94
$\mathbf{x}_{\text{new}}^{\mathsf{S}}$	-3.73	0.07	0.06	0.00	-0.04	0.16	0.15	0.93
$\mathbf{x}_{\text{new}}^{\mathsf{I}}$	-0.42	0.14	0.03	0.00	-0.03	0.13	0.12	0.92
$\mathbf{x}_{\text{new}}^{\mathsf{D}}$	-0.03	0.15	0.03	0.28	-0.02	0.12	0.12	0.95
Large $\mathcal{S}(\boldsymbol{\beta}_0)$, moderate surrogates								
$\mathbf{x}_{\text{new}}^{\mathsf{L}}$	0.61	0.70	0.41	0.56	-0.22	1.98	1.96	0.95
$\mathbf{x}_{\text{new}}^{\mathsf{M}}$	-0.04	2.54	0.43	0.29	-0.05	2.06	2.02	0.95
$\mathbf{x}_{\text{new}}^{\mathsf{H}}$	-2.53	4.66	0.45	0.11	0.47	2.16	2.10	0.94
$\mathbf{x}_{\text{new}}^{\mathsf{S}}$	-3.56	0.11	0.06	0.00	-0.03	0.16	0.15	0.92
$\mathbf{x}_{\text{new}}^{\mathsf{I}}$	-0.70	0.14	0.03	0.00	-0.02	0.13	0.12	0.91
$\mathbf{x}_{\text{new}}^{\mathsf{D}}$	-0.30	0.13	0.03	0.02	-0.02	0.13	0.12	0.93
Moderate $\mathcal{S}(\boldsymbol{\beta}_0)$, strong surrogates								
$\mathbf{x}_{\text{new}}^{\mathsf{L}}$	0.89	0.67	0.41	0.37	-0.22	1.69	1.67	0.95
$\mathbf{x}_{\text{new}}^{\mathsf{M}}$	-0.15	2.51	0.42	0.27	-0.04	1.78	1.71	0.94
$\mathbf{x}_{\text{new}}^{\mathsf{H}}$	-3.28	4.57	0.44	0.10	0.48	1.86	1.79	0.93
$\mathbf{x}_{\text{new}}^{\mathsf{S}}$	-3.83	0.07	0.07	0.00	-0.02	0.14	0.13	0.93
$\mathbf{x}_{\text{new}}^{\mathsf{I}}$	-0.99	0.12	0.03	0.00	-0.00	0.11	0.10	0.92
$\mathbf{x}_{\text{new}}^{\mathsf{D}}$	-0.57	0.12	0.03	0.00	-0.01	0.11	0.10	0.93
Large $\mathcal{S}(\boldsymbol{\beta}_0)$, strong surrogates								
$\mathbf{x}_{\text{new}}^{\mathsf{L}}$	0.76	0.82	0.40	0.41	-0.27	1.67	1.65	0.95
$\mathbf{x}_{\text{new}}^{\mathsf{M}}$	0.09	2.35	0.42	0.26	-0.03	1.81	1.73	0.94
$\mathbf{x}_{\text{new}}^{\mathsf{H}}$	-4.10	3.97	0.45	0.10	0.45	1.88	1.83	0.94
$\mathbf{x}_{\text{new}}^{\mathsf{S}}$	-3.99	0.09	0.07	0.00	-0.03	0.14	0.14	0.93
$\mathbf{x}_{\text{new}}^{\mathsf{I}}$	-0.82	0.12	0.03	0.00	-0.01	0.11	0.10	0.93
$\mathbf{x}_{\text{new}}^{\mathsf{D}}$	-0.37	0.14	0.03	0.01	-0.01	0.11	0.11	0.97

Appendix B. Proofs of Main Results

We first summarize below notations used Section 3 for the conditional expectations given different part of the data.

Definition 11 *The conditional expectation for samples with index in set $\mathcal{S}$ conditionally on subset of the data $\mathscr{D}$ is denoted as*

$$\mathbb{E}_{i\in\mathcal{S}}\{f(Y_i,\mathbf{X}_i,\mathbf{S}_i)\mid\mathscr{D}\},\ \mathcal{S}\subseteq\{1,\dots,n+N\},\mathscr{D}\subset\mathscr{L}\cup\mathscr{U}.$$

We denote the conditional expectation of unlabeled data given labelled data by $\mathbb{E}_{i>n}\{f(\mathbf{W}_i)\mid\mathscr{L}\}$ and the conditional probability of new copy of data given current data by $\mathbb{P}_{\text{new}}\{f(\mathbf{W}_i)\mid\mathscr{D}\}$. With $\mathscr{L}$ and $\mathscr{U}$ partitioned into K folds indexed respectively by $\{\mathcal{I}_k,k=1,...,K\}$ and $\{\mathcal{J}_k,k=1,...,K\}$, we denote the conditional expectation of fold-k labelled data and unlabeled data given the out-of-fold data respectively by

$$\mathbb{E}_{i\in\mathcal{I}_k}\{f(Y_i,\mathbf{X}_i,\mathbf{S}_i)\mid\mathscr{D}_k^c\}\quad\text{and}\quad\mathbb{E}_{i\in\mathcal{J}_k}\{f(\mathbf{W}_i)\mid\mathscr{D}_k^c\},$$
$$\text{where}\quad\mathscr{D}_k^c=\{\mathbf{S}_i,\mathbf{X}_i,i\in\mathcal{J}_k^c\}\cup\{Y_i,\mathbf{S}_i,\mathbf{X}_i,i\in\mathcal{I}_k^c\}.$$

B1 Proof of Theorem 1

Our proof shares the general steps with the the restricted strong convexity framework laid down in Negahban et al. (2010) while we have a delicate analysis of the symmetrized Bregman divergence to establish the improved rate of estimation under semi-supervised learning setting. To bound $\widehat{\boldsymbol\beta}$ through the symmetrized Bregman divergence $(\widehat{\boldsymbol\beta}-\boldsymbol\beta_0)^\intercal\dot{\boldsymbol\ell}^\dagger(\boldsymbol\beta_0;\widehat{\boldsymbol\gamma})$, instead of directly applying the Hölder's bound, we first split it into two parts,

$$(\widehat{\boldsymbol\beta}-\boldsymbol\beta_0)^\intercal\dot{\boldsymbol\ell}^\dagger(\boldsymbol\beta_0;\widehat{\boldsymbol\gamma})=\underbrace{(\widehat{\boldsymbol\beta}-\boldsymbol\beta_0)^\intercal\left[\dot{\boldsymbol\ell}^\dagger(\boldsymbol\beta_0;\widehat{\boldsymbol\gamma})-\mathbb{E}\{\dot{\boldsymbol\ell}^\dagger(\boldsymbol\beta_0;\widehat{\boldsymbol\gamma})\mid\mathscr{L}\}+\mathbb{E}\{\dot{\boldsymbol\ell}^\dagger(\boldsymbol\beta_0;\boldsymbol\gamma_0)\mid\mathscr{L}\}\right]}_{\text{variance from unlabeled data}}$$
$$+\underbrace{(\widehat{\boldsymbol\beta}-\boldsymbol\beta_0)^\intercal\mathbb{E}\left\{\dot{\boldsymbol\ell}^\dagger(\boldsymbol\beta_0;\widehat{\boldsymbol\gamma})-\dot{\boldsymbol\ell}^\dagger(\boldsymbol\beta_0;\boldsymbol\gamma_0)\mid\mathscr{L}\right\}}_{\text{bias from }\widehat{\boldsymbol\gamma}}\tag{A.1}$$

and discuss which part dominates the estimation error. When the first variance term in (A.1) is dominant, the bias from $\widehat{\boldsymbol\gamma}$ becomes eligible. Then, we should recover the usual error bound for LASSO as if $\boldsymbol\gamma_0$ is used. When the second bias term in (A.1) is dominant, the error bound of $\widehat{\boldsymbol\beta}$ can be controlled by the error bound of $\widehat{\boldsymbol\gamma}$. Combining the error bounds in the two cases, we obtain the oracle inequalities.

Lemma 12 *On event*

$$\Omega=\Big\{\ell_{\mathsf{PL}}(\boldsymbol\beta_0+\Delta)-\ell_{\mathsf{PL}}(\boldsymbol\beta_0)-\Delta^\intercal\dot{\boldsymbol\ell}_{\mathsf{PL}}(\boldsymbol\beta_0)$$
$$\geq\kappa_{\mathsf{rsc},1}\|\Delta\|_2\{\|\Delta\|_2-\kappa_{\mathsf{rsc},2}\sqrt{\log(p)/N}\|\Delta\|_1\},\forall\|\Delta\|_2\leq1\Big\},$$

setting $\lambda_\beta\gtrsim\sqrt{\log(p)/N}$ such that

$$\lambda_\beta\geq3\left\|\dot{\boldsymbol\ell}^\dagger(\boldsymbol\beta_0;\widehat{\boldsymbol\gamma})-\mathbb{E}\{\dot{\boldsymbol\ell}^\dagger(\boldsymbol\beta_0;\widehat{\boldsymbol\gamma})\mid\mathscr{L}\}+\mathbb{E}\{\dot{\boldsymbol\ell}^\dagger(\boldsymbol\beta_0;\boldsymbol\gamma_0)\mid\mathscr{L}\}\right\|_\infty+\kappa_{\mathsf{rsc},1}\kappa_{\mathsf{rsc},2}\sqrt{\frac{\log(p)}{N}},$$

we have the oracle inequalities for estimation error of $\widehat{\boldsymbol{\beta}}$,

$$\|\widehat{\boldsymbol{\beta}}-\boldsymbol{\beta}_0\|_2 \leq \max\left\{14\sqrt{s_{\beta}}\lambda_{\beta}/\kappa_{\mathsf{rsc},1}, (1-\rho)7M\sigma_{\mathsf{max}}^2\|\boldsymbol{\gamma}_0-\widehat{\boldsymbol{\gamma}}\|_2/\kappa_{\mathsf{rsc},1}\right\},$$
$$\|\widehat{\boldsymbol{\beta}}-\boldsymbol{\beta}_0\|_1 \leq \max\left\{84 s_{\beta}\lambda_{\beta}/\kappa_{\mathsf{rsc},1}, (1-\rho)^2 21 M^2\sigma_{\mathsf{max}}^4\|\boldsymbol{\gamma}_0-\widehat{\boldsymbol{\gamma}}\|_2^2/(\kappa_{\mathsf{rsc},1}\lambda_{\beta})\right\}.$$

The constants $\kappa_{\mathsf{rsc},1}, \kappa_{\mathsf{rsc},2}$ are the restrictive strong convexity parameters specified in Lemma 19.

We next prove the oracle inequalities. First, we note that by the definition of $\widehat{\boldsymbol{\beta}}$,

$$\ell^{\dagger}(\widehat{\boldsymbol{\beta}};\widehat{\boldsymbol{\gamma}}) + \lambda_{\beta}\|\widehat{\boldsymbol{\beta}}\|_1 \leq \ell^{\dagger}(\boldsymbol{\beta}_0;\widehat{\boldsymbol{\gamma}}) + \lambda_{\beta}\|\boldsymbol{\beta}_0\|_1. \tag{A.2}$$

Denote the standardized estimation error as $\boldsymbol{\delta} = (\widehat{\boldsymbol{\beta}}-\boldsymbol{\beta}_0)/\|\widehat{\boldsymbol{\beta}}-\boldsymbol{\beta}_0\|_2$. Due to convexity of the loss function, we have for $t = \|\widehat{\boldsymbol{\beta}}-\boldsymbol{\beta}_0\|_2 \wedge 1$

$$\ell^{\dagger}(\boldsymbol{\beta}_0+t\boldsymbol{\delta};\widehat{\boldsymbol{\gamma}}) + \lambda_{\beta}\|\boldsymbol{\beta}_0+t\boldsymbol{\delta}\|_1 \leq \ell^{\dagger}(\boldsymbol{\beta}_0;\widehat{\boldsymbol{\gamma}}) + \lambda_{\beta}\|\boldsymbol{\beta}_0\|_1. \tag{A.3}$$

By the triangle inequality $\|\boldsymbol{\beta}_0\|_1 - \|\boldsymbol{\beta}_0+t\boldsymbol{\delta}\|_1 \leq t\|\boldsymbol{\delta}\|_1$, we have from (A.3)

$$\ell^{\dagger}(\boldsymbol{\beta}_0+t\boldsymbol{\delta};\widehat{\boldsymbol{\gamma}}) - \ell^{\dagger}(\boldsymbol{\beta}_0;\widehat{\boldsymbol{\gamma}}) \leq t\lambda_{\beta}\|\boldsymbol{\delta}\|_1 \tag{A.4}$$

To apply the restricted strong convexity of the complete data loss (11) established in Lemma 19, we show that the second order approximation error of the imputed loss is equivalent to that of the complete data loss,

$$\ell^{\dagger}(\boldsymbol{\beta}_0+t\boldsymbol{\delta};\widehat{\boldsymbol{\gamma}}) - \ell^{\dagger}(\boldsymbol{\beta}_0;\widehat{\boldsymbol{\gamma}}) - t\boldsymbol{\delta}^{\mathsf{T}}\dot{\boldsymbol{\ell}}^{\dagger}(\boldsymbol{\beta}_0;\widehat{\boldsymbol{\gamma}}) = \ell_{\mathsf{PL}}(\boldsymbol{\beta}_0+t\boldsymbol{\delta}) - \ell_{\mathsf{PL}}(\boldsymbol{\beta}_0) - t\boldsymbol{\delta}^{\mathsf{T}}\dot{\boldsymbol{\ell}}_{\mathsf{PL}}(\boldsymbol{\beta}_0).$$

Then by applying the restricted strong convexity event Ω, we obtain

$$\ell^{\dagger}(\boldsymbol{\beta}_0+t\boldsymbol{\delta};\widehat{\boldsymbol{\gamma}}) - \ell^{\dagger}(\boldsymbol{\beta}_0;\widehat{\boldsymbol{\gamma}}) - t\boldsymbol{\delta}^{\mathsf{T}}\dot{\boldsymbol{\ell}}^{\dagger}(\boldsymbol{\beta}_0;\widehat{\boldsymbol{\gamma}}) \geq t^2\kappa_{\mathsf{rsc},1} - t\kappa_{\mathsf{rsc},1}\kappa_{\mathsf{rsc},2}\sqrt{\log(p)/N}\|\boldsymbol{\delta}\|_1. \tag{A.5}$$

Applying (A.5) to (A.4), we have with large probability

$$t\boldsymbol{\delta}^{\mathsf{T}}\dot{\boldsymbol{\ell}}^{\dagger}(\boldsymbol{\beta}_0;\widehat{\boldsymbol{\gamma}}) + t^2\kappa_{\mathsf{rsc},1} - t\kappa_{\mathsf{rsc},1}\kappa_{\mathsf{rsc},2}\sqrt{\log(p)/N}\|\boldsymbol{\delta}\|_1 \leq t\lambda_{\beta}\|\boldsymbol{\delta}\|_1$$

where $\|\boldsymbol{\delta}\|_2 = 1$ from definition. Thus, we have reach

$$t\kappa_{\mathsf{rsc},1} \leq \lambda_{\beta}\|\boldsymbol{\delta}\|_1 - \boldsymbol{\delta}^{\mathsf{T}}\dot{\boldsymbol{\ell}}^{\dagger}(\boldsymbol{\beta}_0;\widehat{\boldsymbol{\gamma}}) + \kappa_{\mathsf{rsc},1}\kappa_{\mathsf{rsc},2}\sqrt{\log(p)/N}\|\boldsymbol{\delta}\|_1. \tag{A.6}$$

Next, we analyze $\boldsymbol{\delta}^{\mathsf{T}}\dot{\boldsymbol{\ell}}^{\dagger}(\boldsymbol{\beta}_0;\widehat{\boldsymbol{\gamma}})$ by the decomposition

$$\begin{aligned}\left|\boldsymbol{\delta}^{\mathsf{T}}\dot{\boldsymbol{\ell}}^{\dagger}(\boldsymbol{\beta}_0;\widehat{\boldsymbol{\gamma}})\right| =& \boldsymbol{\delta}^{\mathsf{T}}\left[\dot{\boldsymbol{\ell}}^{\dagger}(\boldsymbol{\beta}_0;\widehat{\boldsymbol{\gamma}}) - \mathbb{E}\{\dot{\boldsymbol{\ell}}^{\dagger}(\boldsymbol{\beta}_0;\widehat{\boldsymbol{\gamma}}) \mid \mathscr{L}\} + \mathbb{E}\{\dot{\boldsymbol{\ell}}^{\dagger}(\boldsymbol{\beta}_0;\boldsymbol{\gamma}_0) \mid \mathscr{L}\}\right] \\ &+ \boldsymbol{\delta}^{\mathsf{T}}\mathbb{E}\left\{\dot{\boldsymbol{\ell}}^{\dagger}(\boldsymbol{\beta}_0;\widehat{\boldsymbol{\gamma}}) - \dot{\boldsymbol{\ell}}^{\dagger}(\boldsymbol{\beta}_0;\boldsymbol{\gamma}_0) \mid \mathscr{L}\right\} \\ \leq& \|\boldsymbol{\delta}\|_1\left\|\dot{\boldsymbol{\ell}}^{\dagger}(\boldsymbol{\beta}_0;\widehat{\boldsymbol{\gamma}}) - \mathbb{E}\{\dot{\boldsymbol{\ell}}^{\dagger}(\boldsymbol{\beta}_0;\widehat{\boldsymbol{\gamma}}) \mid \mathscr{L}\} + \mathbb{E}\{\dot{\boldsymbol{\ell}}^{\dagger}(\boldsymbol{\beta}_0;\boldsymbol{\gamma}_0) \mid \mathscr{L}\}\right\|_{\infty} \\ &+ (1-\rho)\left\|\mathbb{E}_{i>n}[\mathbf{X}_i\{g(\boldsymbol{\beta}_0^{\mathsf{T}}\mathbf{X}_i) - g(\widehat{\boldsymbol{\gamma}}^{\mathsf{T}}\mathbf{W}_i)\} \mid \mathscr{L}]\right\|_2.\end{aligned} \tag{A.7}$$

To establish the rate for L_2-norm of $\mathbb{E}\{\dot{\boldsymbol{\ell}}^\dagger(\boldsymbol{\beta}_0;\widehat{\boldsymbol{\gamma}}) \mid \mathscr{L}\}$, we note that

$$\mathbb{E}_{i>n}[\mathbf{X}_i\{g(\boldsymbol{\beta}_0^\intercal\mathbf{X}_i) - g(\widehat{\boldsymbol{\gamma}}^\intercal\mathbf{W}_i)\} \mid \mathscr{L}] = \mathbb{E}_{i>n}\left[\mathbf{X}_i\left\{Y_i - g\left(\widehat{\boldsymbol{\gamma}}^\intercal\mathbf{W}_i\right)\right\} \mid \mathscr{L}\right]. \tag{A.8}$$

By the characterization of $\boldsymbol{\gamma}_0$ as in (6), we may rewrite (A.8) as

$$\mathbb{E}_{i>n}\left[\mathbf{X}_i\left\{Y_i - g\left(\widehat{\boldsymbol{\gamma}}^\intercal\mathbf{W}_i\right)\right\} \mid \mathscr{L}\right] = \mathbb{E}_{i>n}\left[g'(\boldsymbol{\gamma}_u^\intercal\mathbf{W}_i)\mathbf{X}_i\mathbf{W}_i^\intercal \mid \mathscr{L}\right](\boldsymbol{\gamma}_0 - \widehat{\boldsymbol{\gamma}}), \tag{A.9}$$

where $\boldsymbol{\gamma}_u = u\widehat{\boldsymbol{\gamma}} + (1-u)\boldsymbol{\gamma}_0$ for some $u \in [0,1]$. Under Assumptions 1b and 2a, as well as the fact that $\mathbf{X}_i$ is a sub-vector of $\mathbf{W}_i$, we have

$$\begin{aligned}\left\|\mathbb{E}\{\dot{\boldsymbol{\ell}}^\dagger(\boldsymbol{\beta}_0;\widehat{\boldsymbol{\gamma}}) \mid \mathscr{L}\}\right\|_2 &\le \left\|\mathbb{E}_{i\in\mathcal{U}}\left[g'(\boldsymbol{\gamma}_u^\intercal\mathbf{W}_i)\mathbf{W}_i^{\otimes 2} \mid \mathscr{L}\right]\right\|_2 \|\boldsymbol{\gamma}_0 - \widehat{\boldsymbol{\gamma}}\|_2 \\ &\le M\left\|\mathbb{E}\left(\mathbf{W}_i^{\otimes 2}\right)\right\|_2 \|\boldsymbol{\gamma}_0 - \widehat{\boldsymbol{\gamma}}\|_2 \le M\sigma_{\mathsf{max}}^2\|\boldsymbol{\gamma}_0 - \widehat{\boldsymbol{\gamma}}\|_2,\end{aligned} \tag{A.10}$$

where for any vector $\mathbf{x}$, $\mathbf{x}^{\otimes 2} = \mathbf{x}\mathbf{x}^\intercal$. By the bound for (A.10) and the definition of $\lambda_{\boldsymbol{\beta}}$, we have the bound from (A.6)

$$t\kappa_{\mathsf{rsc},1} \le 2\lambda_{\boldsymbol{\beta}}\|\boldsymbol{\delta}\|_1 + (1-\rho)M\sigma_{\mathsf{max}}^2\|\boldsymbol{\gamma}_0 - \widehat{\boldsymbol{\gamma}}\|_2. \tag{A.11}$$

Hence, we can reach an immediate bound for estimation error from (A.11) without considering the sparsity of $\boldsymbol{\beta}_0$. We shall proceed to derive a sharper bound that involves the sparsity of $\boldsymbol{\beta}_0$. We separately analyze two cases.

$$\underline{\textbf{Case 1:}} \quad (1-\rho)M\sigma_{\mathsf{max}}^2\|\boldsymbol{\gamma}_0 - \widehat{\boldsymbol{\gamma}}\|_2 \ge \|\boldsymbol{\delta}\|_1\lambda_{\boldsymbol{\beta}}/3.$$

In this case, the estimation error is dominated by $\widehat{\boldsymbol{\gamma}} - \boldsymbol{\gamma}_0$. We simply have from (A.11)

$$\begin{aligned} t\kappa_{\mathsf{rsc},1} &\le 7(1-\rho)M\sigma_{\mathsf{max}}^2\|\boldsymbol{\gamma}_0 - \widehat{\boldsymbol{\gamma}}\|_2, \\ t\kappa_{\mathsf{rsc},1}\|\boldsymbol{\delta}\|_1\lambda_{\boldsymbol{\beta}}/3 &\le 7(1-\rho)^2M^2\sigma_{\mathsf{max}}^4\|\boldsymbol{\gamma}_0 - \widehat{\boldsymbol{\gamma}}\|_2^2. \end{aligned}$$

Thus, we have

$$\begin{aligned} \|\widehat{\boldsymbol{\beta}} - \boldsymbol{\beta}_0\|_2 &\le (1-\rho)7M\sigma_{\mathsf{max}}^2\|\boldsymbol{\gamma}_0 - \widehat{\boldsymbol{\gamma}}\|_2/\kappa_{\mathsf{rsc},1}, \\ \|\widehat{\boldsymbol{\beta}} - \boldsymbol{\beta}_0\|_1 &\le (1-\rho)^2 21M^2\sigma_{\mathsf{max}}^4\|\boldsymbol{\gamma}_0 - \widehat{\boldsymbol{\gamma}}\|_2^2/(\kappa_{\mathsf{rsc},1}\lambda_{\boldsymbol{\beta}}). \end{aligned} \tag{A.12}$$

If case 1 does not hold, then instead

$$\underline{\textbf{Case 2:}} \quad (1-\rho)M\sigma_{\mathsf{max}}^2\|\boldsymbol{\gamma}_0 - \widehat{\boldsymbol{\gamma}}\|_2 \le \|\boldsymbol{\delta}\|_1\lambda_{\boldsymbol{\beta}}/3. \tag{A.13}$$

In this case, the estimation error is comparable to that when we have the true $\boldsymbol{\gamma}_0$ for the imputation. Thus, the sparsity of $\boldsymbol{\beta}_0$ may affect the estimation error.

Following the typical approach to establish the cone condition for $\boldsymbol{\delta}$, we analyze the symmetrized Bregman's divergence,

$$(\widehat{\boldsymbol{\beta}} - \boldsymbol{\beta}_0)^\intercal\left\{\dot{\boldsymbol{\ell}}^\dagger(\widehat{\boldsymbol{\beta}};\widehat{\boldsymbol{\gamma}}) - \dot{\boldsymbol{\ell}}^\dagger(\boldsymbol{\beta}_0;\widehat{\boldsymbol{\gamma}})\right\} = \|\widehat{\boldsymbol{\beta}} - \boldsymbol{\beta}_0\|_2\boldsymbol{\delta}^\intercal\left\{\dot{\boldsymbol{\ell}}^\dagger(\widehat{\boldsymbol{\beta}};\widehat{\boldsymbol{\gamma}}) - \dot{\boldsymbol{\ell}}^\dagger(\boldsymbol{\beta}_0;\widehat{\boldsymbol{\gamma}})\right\}. \tag{A.14}$$

Due to the convexity of the loss $\dot{\boldsymbol{\ell}}^{\dagger}(\cdot;\widehat{\boldsymbol{\gamma}})$ under Assumption 1b, the symmetrized Bregman's divergence (A.14) is nonnegative through a mean-value theorem,

$$(\widehat{\boldsymbol{\beta}}-\boldsymbol{\beta}_0)^{\mathsf{T}}\left\{\dot{\boldsymbol{\ell}}^{\dagger}(\widehat{\boldsymbol{\beta}};\widehat{\boldsymbol{\gamma}})-\dot{\boldsymbol{\ell}}^{\dagger}(\boldsymbol{\beta}_0;\widehat{\boldsymbol{\gamma}})\right\}\geq\inf_{\boldsymbol{\beta}\in\mathbb{R}^{p+1}}\frac{1}{N}\sum_{i>n}g'(\boldsymbol{\beta}^{\mathsf{T}}\mathbf{X}_i)\{(\widehat{\boldsymbol{\beta}}-\boldsymbol{\beta}_0)^{\mathsf{T}}\mathbf{X}_i\}^2\geq 0.$$

Denote the indices set of nonzero coefficient in $\boldsymbol{\beta}_0$ as $\mathcal{O}_{\boldsymbol{\beta}}=\{j:\boldsymbol{\beta}_{0,j}\neq 0\}$. We denote the $\boldsymbol{\delta}_{\mathcal{O}_{\boldsymbol{\beta}}}$ and $\boldsymbol{\delta}_{\mathcal{O}_{\boldsymbol{\beta}}^{\mathsf{c}}}$ as the sub-vectors for $\boldsymbol{\delta}$ at positions in $\mathcal{O}_{\boldsymbol{\beta}}$ and at positions not in $\mathcal{O}_{\boldsymbol{\beta}}$, respectively. The solution $\widehat{\boldsymbol{\beta}}$ satisfies the KKT condition

$$\|\dot{\boldsymbol{\ell}}^{\dagger}(\widehat{\boldsymbol{\beta}};\widehat{\boldsymbol{\gamma}})\|_{\infty}\leq\lambda_{\boldsymbol{\beta}},\ \dot{\ell}^{\dagger}(\widehat{\boldsymbol{\beta}};\widehat{\boldsymbol{\gamma}})_j=-\lambda_{\boldsymbol{\beta}}\operatorname{sign}(\widehat{\boldsymbol{\beta}}_j),\ j:\widehat{\beta}_j\neq 0.$$

From the KKT condition and the definitions of $\boldsymbol{\delta}$ and $\mathcal{O}_{\boldsymbol{\beta}}$, we have

$$\delta_j\dot{\ell}^{\dagger}(\widehat{\boldsymbol{\beta}};\widehat{\boldsymbol{\gamma}})_j\leq|\delta_j|\lambda_{\boldsymbol{\beta}},\ j\in\mathcal{O}_{\boldsymbol{\beta}};\ \delta_j\dot{\ell}^{\dagger}(\widehat{\boldsymbol{\beta}};\widehat{\boldsymbol{\gamma}})_j=\frac{-\widehat{\beta}_j\lambda_{\boldsymbol{\beta}}\operatorname{sign}(\widehat{\beta}_j)}{\|\widehat{\boldsymbol{\beta}}-\boldsymbol{\beta}_0\|_2}=-\lambda_{\boldsymbol{\beta}}|\delta_j|,\ j\in\mathcal{O}_{\boldsymbol{\beta}}^{\mathsf{c}}. \tag{A.15}$$

Applying the (A.15) to (A.14), we have the upper bound,

$$\begin{aligned}\boldsymbol{\delta}^{\mathsf{T}}\left\{\dot{\boldsymbol{\ell}}^{\dagger}(\boldsymbol{\beta}_0;\widehat{\boldsymbol{\gamma}})-\dot{\boldsymbol{\ell}}^{\dagger}(\boldsymbol{\beta}_0;\widehat{\boldsymbol{\gamma}})\right\}&=\sum_{j\in\mathcal{O}_{\boldsymbol{\beta}}}\delta_j\dot{\ell}^{\dagger}(\widehat{\boldsymbol{\beta}};\widehat{\boldsymbol{\gamma}})_j+\sum_{j\in\mathcal{O}_{\boldsymbol{\beta}}^{\mathsf{c}}}\delta_j\dot{\ell}^{\dagger}(\widehat{\boldsymbol{\beta}};\widehat{\boldsymbol{\gamma}})_j+\boldsymbol{\delta}^{\mathsf{T}}\dot{\boldsymbol{\ell}}^{\dagger}(\boldsymbol{\beta}_0;\widehat{\boldsymbol{\gamma}})\\&\leq\lambda_{\boldsymbol{\beta}}\sum_{j\in\mathcal{O}_{\boldsymbol{\beta}}}|\delta_j|-\lambda_{\boldsymbol{\beta}}\sum_{j\in\mathcal{O}_{\boldsymbol{\beta}}^{\mathsf{c}}}|\delta_j|+\boldsymbol{\delta}^{\mathsf{T}}\dot{\boldsymbol{\ell}}^{\dagger}(\boldsymbol{\beta}_0;\widehat{\boldsymbol{\gamma}})\\&\leq\lambda_{\boldsymbol{\beta}}\|\boldsymbol{\delta}_{\mathcal{O}_{\boldsymbol{\beta}}}\|_1-\lambda_{\boldsymbol{\beta}}\|\boldsymbol{\delta}_{\mathcal{O}_{\boldsymbol{\beta}}^{\mathsf{c}}}\|_1+\left|\boldsymbol{\delta}^{\mathsf{T}}\dot{\boldsymbol{\ell}}^{\dagger}(\boldsymbol{\beta}_0;\widehat{\boldsymbol{\gamma}})\right|.\end{aligned}$$

Then, we apply (A.10), the definition of $\lambda_{\boldsymbol{\beta}}$ and (A.13),

$$0\leq\lambda_{\boldsymbol{\beta}}\|\boldsymbol{\delta}_{\mathcal{O}_{\boldsymbol{\beta}}}\|_1-\lambda_{\boldsymbol{\beta}}\|\boldsymbol{\delta}_{\mathcal{O}_{\boldsymbol{\beta}}^{\mathsf{c}}}\|_1+\frac{2}{3}\lambda_{\boldsymbol{\beta}}\|\boldsymbol{\delta}\|_1\quad\text{and}\quad\lambda_{\boldsymbol{\beta}}\|\boldsymbol{\delta}_{\mathcal{O}_{\boldsymbol{\beta}}^{\mathsf{c}}}\|_1\leq 5\lambda_{\boldsymbol{\beta}}\|\boldsymbol{\delta}_{\mathcal{O}_{\boldsymbol{\beta}}}\|_1.$$

Therefore, we can bound the L_1 norm of $\boldsymbol{\delta}$ by the cone property,

$$\|\boldsymbol{\delta}\|_1\leq 6\lambda_{\boldsymbol{\beta}}\|\boldsymbol{\delta}_{\mathcal{O}_{\boldsymbol{\beta}}}\|_1\leq 6\sqrt{s_{\boldsymbol{\beta}}}\|\boldsymbol{\delta}\|_2=6\sqrt{s_{\boldsymbol{\beta}}}. \tag{A.16}$$

We then apply the cone condition (A.16) and the case condition (A.13) to the bound (A.11),

$$t\kappa_{\mathsf{rsc},1}\leq 14\sqrt{s_{\boldsymbol{\beta}}}\lambda_{\boldsymbol{\beta}},\qquad\text{and}\quad t\kappa_{\mathsf{rsc},1}\|\boldsymbol{\delta}\|_1\leq 84s_{\boldsymbol{\beta}}\lambda_{\boldsymbol{\beta}}$$

Thus, we obtain the rate for estimation error

$$\|\widehat{\boldsymbol{\beta}}-\boldsymbol{\beta}_0\|_2\leq 14\sqrt{s_{\boldsymbol{\beta}}}\lambda_{\boldsymbol{\beta}}/\kappa_{\mathsf{rsc},1},\qquad\text{and}\quad\|\widehat{\boldsymbol{\beta}}-\boldsymbol{\beta}_0\|_1\leq 84s_{\boldsymbol{\beta}}\lambda_{\boldsymbol{\beta}}/\kappa_{\mathsf{rsc},1}. \tag{A.17}$$

Since Case 1 and Case 2 are the complement of each other, one of them must occur. Thus, the bound of estimation error is controlled by the larger bound in the two cases,

$$\begin{aligned}&\|\widehat{\boldsymbol{\beta}}-\boldsymbol{\beta}_0\|_2\leq\max\left\{14\sqrt{s_{\boldsymbol{\beta}}}\lambda_{\boldsymbol{\beta}}/\kappa_{\mathsf{rsc},1},(1-\rho)7M\sigma_{\mathsf{max}}^2\|\boldsymbol{\gamma}_0-\widehat{\boldsymbol{\gamma}}\|_2/\kappa_{\mathsf{rsc},1}\right\},\\&\|\widehat{\boldsymbol{\beta}}-\boldsymbol{\beta}_0\|_1\leq\max\left\{84s_{\boldsymbol{\beta}}\lambda_{\boldsymbol{\beta}}/\kappa_{\mathsf{rsc},1},(1-\rho)^2 21M^2\sigma_{\mathsf{max}}^4\|\boldsymbol{\gamma}_0-\widehat{\boldsymbol{\gamma}}\|_2^2/(\kappa_{\mathsf{rsc},1}\lambda_{\boldsymbol{\beta}})\right\},\end{aligned}$$

which is our oracle inequality in Lemma 12.

Consistency We next show that the oracle inequality leads to the consistency under dimension condition (26). To show

$$\begin{aligned}&\left\|\dot{\boldsymbol{\ell}}^{\dagger}(\boldsymbol{\beta}_0;\widehat{\boldsymbol{\gamma}}) - \mathbb{E}\{\dot{\boldsymbol{\ell}}^{\dagger}(\boldsymbol{\beta}_0;\widehat{\boldsymbol{\gamma}}) \mid \mathscr{L}\} + \mathbb{E}\{\dot{\boldsymbol{\ell}}^{\dagger}(\boldsymbol{\beta}_0;\boldsymbol{\gamma}_0) \mid \mathscr{L}\}\right\|_{\infty}\\ \le &\left\|\dot{\boldsymbol{\ell}}^{\dagger}(\boldsymbol{\beta}_0;\widehat{\boldsymbol{\gamma}}) - \mathbb{E}\{\dot{\boldsymbol{\ell}}^{\dagger}(\boldsymbol{\beta}_0;\widehat{\boldsymbol{\gamma}}) \mid \mathscr{L}\}\right\|_{\infty} + \left\|\mathbb{E}\{\dot{\boldsymbol{\ell}}^{\dagger}(\boldsymbol{\beta}_0;\boldsymbol{\gamma}_0) \mid \mathscr{L}\}\right\|_{\infty} = O_p\left(\sqrt{\log(p)/N}\right),\end{aligned}$$

we express the term of interest as the sum of the following empirical processes

$$\begin{aligned}\dot{\boldsymbol{\ell}}^{\dagger}(\boldsymbol{\beta}_0;\widehat{\boldsymbol{\gamma}}) - \mathbb{E}\{\dot{\boldsymbol{\ell}}^{\dagger}(\boldsymbol{\beta}_0;\widehat{\boldsymbol{\gamma}}) \mid \mathscr{L}\} =& \frac{1}{N}\sum_{i>n}\Big(\mathbf{X}_i\{g(\boldsymbol{\beta}_0^{\mathsf{T}}\mathbf{X}_i) - Y_i + Y_i - g(\widehat{\boldsymbol{\gamma}}^{\mathsf{T}}\mathbf{W}_i)\}\\ &- \mathbb{E}_{i>n}\left[\mathbf{X}_i\{g(\boldsymbol{\beta}_0^{\mathsf{T}}\mathbf{X}_i) - Y_i + Y_i - g(\widehat{\boldsymbol{\gamma}}^{\mathsf{T}}\mathbf{W}_i)\} \mid \mathscr{L}\right]\Big),\\ \mathbb{E}\{\dot{\boldsymbol{\ell}}^{\dagger}(\boldsymbol{\beta}_0;\boldsymbol{\gamma}_0) \mid \mathscr{L}\} =& \frac{1}{N}\sum_{i=1}^{n}\mathbf{X}_i\{g(\boldsymbol{\beta}_0^{\mathsf{T}}\mathbf{X}_i) - Y_i\}.\end{aligned}$$

Under Assumption 1a and 1a, $\mathbf{X}_i$ and $g(\boldsymbol{\beta}_0^{\mathsf{T}}\mathbf{X}_i) - Y_i$ are sub-Gaussian. According to Lemma 21, the event $\|\widehat{\boldsymbol{\gamma}} - \boldsymbol{\gamma}_0\|_2 \le 1$ occurs with large probability, on which we have a bound for the sub-Gaussian norm of $Y_i - g(\widehat{\boldsymbol{\gamma}}^{\mathsf{T}}\mathbf{W}_i)$ by Lemma 14.

$$\|Y_i - g(\widehat{\boldsymbol{\gamma}}^{\mathsf{T}}\mathbf{W}_i)\|_{\psi_2} \le \max\{2\nu_1, M\sqrt{2}\sigma_{\max}\},\ i > n \tag{A.18}$$

Thus, we obtain from (A.18) that $Y_i - g(\widehat{\boldsymbol{\gamma}}^{\mathsf{T}}\mathbf{W}_i)$ is sub-Gaussian with large probability. Thus by the properties of sub-Gaussian random variables in Lemma 17-d and 17-f, we have established that the elements in the summands of $\dot{\boldsymbol{\ell}}^{\dagger}(\boldsymbol{\beta}_0;\widehat{\boldsymbol{\gamma}})$ are all sub-exponential random variables conditionally on the labelled data. We apply the Bernstein's inequality (Lemma 17-h) conditionally on the labelled data to obtain

$$\begin{aligned}\left\|\dot{\boldsymbol{\ell}}^{\dagger}(\boldsymbol{\beta}_0;\widehat{\boldsymbol{\gamma}}) - \mathbb{E}\{\dot{\boldsymbol{\ell}}^{\dagger}(\boldsymbol{\beta}_0;\widehat{\boldsymbol{\gamma}}) \mid \mathscr{L}\}\right\|_{\infty} &= O_p\left(\sqrt{(1-\rho)\log(p)/N}\right),\\ \left\|\mathbb{E}\{\dot{\boldsymbol{\ell}}^{\dagger}(\boldsymbol{\beta}_0;\boldsymbol{\gamma}_0) \mid \mathscr{L}\}\right\|_{\infty} &= O_p\left(\sqrt{\rho\log(p)/N}\right).\end{aligned}$$

This establishes the order for $\lambda_{\boldsymbol{\beta}}$,

$$\lambda_{\boldsymbol{\beta}} \gtrsim \sqrt{(1-\rho)\log(p)/N} + \sqrt{\rho\log(p)/N} \asymp \sqrt{\log(p)/N}. \tag{A.19}$$

By Lemma 19 from Negahban et al. (2010), we have that the probability of restricted strong convexity event converges to one,

$$\mathbb{P}(\Omega) \ge 1 - \kappa_{\mathsf{rsc},3}e^{\kappa_{\mathsf{rsc},4}N} \to 1.$$

Setting $\lambda_{\boldsymbol{\beta}} \asymp \sqrt{\log(p)/N}$ for optimal L_2 estimation, we achieve the stated conclusion

$$\begin{aligned}\|\widehat{\boldsymbol{\beta}} - \boldsymbol{\beta}_0\|_2 &= O_p\left(\sqrt{s_{\boldsymbol{\beta}}\log(p)/N} + (1-\rho)\sqrt{s_{\gamma}\log(p+q)/n}\right),\\ \sqrt{\log(p)/N}\|\widehat{\boldsymbol{\beta}} - \boldsymbol{\beta}_0\|_1 &= O_p\left(s_{\boldsymbol{\beta}}\log(p)/N + (1-\rho)^2\frac{s_{\gamma}\log(p+q)}{n}\right),\end{aligned}$$

by applying the rates from Lemma 21 and (A.19). For optimal L_1 estimation, we set a larger penalty $\lambda'_{\boldsymbol{\beta}} \asymp \sqrt{\log(p)/N} \vee \sqrt{s_{\gamma}\log(p+q)/(s_{\boldsymbol{\beta}}n)} \gtrsim \lambda_{\boldsymbol{\beta}}$ to achieve

$$\|\widehat{\boldsymbol{\beta}} - \boldsymbol{\beta}_0\|_1 = O_p\left(s_{\boldsymbol{\beta}}\sqrt{\log(p)/N} + (1-\rho)^2\sqrt{\frac{s_{\gamma}s_{\boldsymbol{\beta}}\log(p+q)}{n}}\right).$$

B2 Proof of Corollary 5

Under Assumption 1b, we have

$$|g(\widehat{\boldsymbol{\beta}}^{\mathsf{T}}\mathbf{x}_{\text{new}}) - g(\boldsymbol{\beta}_0^{\mathsf{T}}\mathbf{x}_{\text{new}})| \le M|(\widehat{\boldsymbol{\beta}} - \boldsymbol{\beta}_0)^{\mathsf{T}}\mathbf{x}_{\text{new}}|. \tag{A.20}$$

Since $\mathbf{x}_{\text{new}}$ satisfies Assumption 2a, we have

$$\|(\widehat{\boldsymbol{\beta}} - \boldsymbol{\beta}_0)^{\mathsf{T}}\mathbf{x}_{\text{new}}\|_{\psi_2} \le \|\widehat{\boldsymbol{\beta}} - \boldsymbol{\beta}_0\|_2\sigma_{\mathsf{max}}/\sqrt{2}.$$

The tail distribution is regulated by the sub-Gaussian norm by Lemma 17-a,

$$\mathbb{P}_{\text{new}}\left(|(\widehat{\boldsymbol{\beta}} - \boldsymbol{\beta}_0)^{\mathsf{T}}\mathbf{x}_{\text{new}}| \ge t \mid \mathscr{D}\right) \le 2\exp\left(-t\sqrt{2}/\left\{\|\widehat{\boldsymbol{\beta}} - \boldsymbol{\beta}_0\|_2\sigma_{\mathsf{max}}\right\}\right). \tag{A.21}$$

Combining (A.20) and (A.21), we obtain

$$\mathbb{P}_{\text{new}}\left(|g(\widehat{\boldsymbol{\beta}}^{\mathsf{T}}\mathbf{x}_{\text{new}}) - g(\boldsymbol{\beta}_0^{\mathsf{T}}\mathbf{x}_{\text{new}})| \ge tM\|\widehat{\boldsymbol{\beta}} - \boldsymbol{\beta}_0\|_2\sigma_{\mathsf{max}}/\sqrt{2} \mid \mathscr{D}\right) \le 2e^{-t}.$$

Thus,

$$\left|g(\widehat{\boldsymbol{\beta}}^{\mathsf{T}}\mathbf{x}_{\text{new}}) - g(\boldsymbol{\beta}_0^{\mathsf{T}}\mathbf{x}_{\text{new}})\right| = O_p\left(\|\widehat{\boldsymbol{\beta}} - \boldsymbol{\beta}_0\|_2\right).$$

B3 Proof of Theorem 7

Our proof is organized in five parts. In Part 1, we establish the consistency of the cross-fitting estimator for precision matrix, namely $\|\widehat{\mathbf{u}}^{(k)} - \mathbf{u}_0\|_2 = o_p(1)$ with $\widehat{\mathbf{u}}^{(k)}$ and $\mathbf{u}_0$ defined in (20) and (19), respectively. In Part 2, we show that the debiased estimator can be approximated by the empirical process

$$\begin{aligned}\sqrt{n}\left(\widehat{\mathbf{x}_{\text{std}}^{\mathsf{T}}\boldsymbol{\beta}} - \mathbf{x}_{\text{std}}^{\mathsf{T}}\boldsymbol{\beta}_0\right) &= -(1-\rho)\sqrt{n}\mathbf{u}_0^{\mathsf{T}}\dot{\boldsymbol{\ell}}_{\mathsf{imp}}(\boldsymbol{\gamma}_0) - \sqrt{n}\mathbf{u}_0^{\mathsf{T}}\dot{\boldsymbol{\ell}}^{\dagger}(\boldsymbol{\beta}_0;\boldsymbol{\gamma}_0) + o_p(1)\\ &= -n^{-\frac{1}{2}}\left[\sum_{i=1}^{n}\mathbf{u}_0^{\mathsf{T}}\mathbf{X}_i\{(1-\rho)\cdot g(\boldsymbol{\gamma}_0^{\mathsf{T}}\mathbf{W}_i) + \rho\cdot g(\boldsymbol{\beta}_0^{\mathsf{T}}\mathbf{X}_i) - Y_i\}\right.\\ &\qquad\left.+\rho\sum_{i>n}\mathbf{X}_i\{g(\boldsymbol{\beta}_0^{\mathsf{T}}\mathbf{X}_i) - g(\boldsymbol{\gamma}_0^{\mathsf{T}}\mathbf{W}_i)\}\right] + o_p(1)\end{aligned}$$

As long as the asymptotic variance V_{SAS} defined in (29) is bounded and bounded away from zero, we have the asymptotic normality of the leading term from the Central Limit Theorem

$$\begin{aligned}-n^{-\frac{1}{2}}V_{\mathsf{SAS}}^{-1/2}&\left[\sum_{i=1}^{n}\mathbf{u}_0^{\mathsf{T}}\mathbf{X}_i\{(1-\rho)\cdot g(\boldsymbol{\gamma}_0^{\mathsf{T}}\mathbf{W}_i) + \rho\cdot g(\boldsymbol{\beta}_0^{\mathsf{T}}\mathbf{X}_i) - Y_i\}\right.\\ &\qquad\left.+\rho\sum_{i>n}\mathbf{X}_i\{g(\boldsymbol{\beta}_0^{\mathsf{T}}\mathbf{X}_i) - g(\boldsymbol{\gamma}_0^{\mathsf{T}}\mathbf{W}_i)\}\right] \rightsquigarrow N(0,1).\end{aligned}$$

In Part 3, we deal with the asymptotic variance V_{SAS} and the consistency of the variance estimator $\widehat{V}_{\mathsf{SAS}}$ defined in (23). In Part 4, we reach the conclusion of the theorem based on,

$$\begin{aligned}&(1-\rho)\|\widehat{\boldsymbol{\gamma}}^{(k)} - \boldsymbol{\gamma}_0\|_2 + \|\widehat{\mathbf{u}}^{(k)} - \mathbf{u}_0\|_2\\ &+\sqrt{n}\|\widehat{\boldsymbol{\beta}}^{(k)} - \boldsymbol{\beta}_0\|_2\left(\|\widehat{\boldsymbol{\beta}}^{(k)} - \boldsymbol{\beta}_0\|_2 + \|\widehat{\mathbf{u}}^{(k)} - \mathbf{u}_0\|_2\right) = o_p(1),\end{aligned} \tag{A.22}$$

for all $1 \le k \le K$. Following Part 4, we show in Part 5 that (28) implies (A.22).

Part 1: Consistency of estimated precision matrix

The definitions of $\mathbf{u}_0$ and $\widehat{\mathbf{u}}^{(k)}$ are given in (19) and (20). In this part, we show

$$\begin{aligned}\|\widehat{\mathbf{u}}^{(k)} - \mathbf{u}_0\|_2 =&O_p\left(\sqrt{(s_{\mathsf{u}}+s_{\boldsymbol{\beta}})\log(p)/(N-N_k)} + (1-\rho)\sqrt{s_\gamma \log(p+q)/(n-n_k)}\right)\\ =&O_p\left(\sqrt{(s_{\mathsf{u}}+s_{\boldsymbol{\beta}})\log(p)/N} + (1-\rho)\sqrt{s_\gamma \log(p+q)/n}\right).\end{aligned}$$

Since we set the number of folds $K \le 10$ to be finite, the estimation rate applies for $\widehat{\mathbf{u}}^{(k)}$ for all $k = 1, \ldots, K$.

We denote the components in the quadratic loss function of (20) and their derivatives as

$$\begin{aligned} m^{(k,k')}(\mathbf{u};\boldsymbol{\beta}) &= \frac{1}{N_{k'}} \sum_{i\in\mathcal{I}_{k'}\cup\mathcal{J}_{k'}} \frac{1}{2} g'(\boldsymbol{\beta}^\intercal \mathbf{X}_i)(\mathbf{X}_i^\intercal\mathbf{u})^2 - \mathbf{u}^\intercal \mathbf{x}_{\text{std}},\\ \dot{\mathbf{m}}^{(k,k')}(\mathbf{u};\boldsymbol{\beta}) &= \frac{\partial}{\partial \mathbf{u}} m^{(k,k')}(\mathbf{u};\boldsymbol{\beta}),\ \ddot{\mathbb{m}}^{(k,k')}(\boldsymbol{\beta}) = \frac{\partial}{\partial \mathbf{u}} \dot{\mathbf{m}}^{(k,k')}(\mathbf{u};\boldsymbol{\beta}) \end{aligned} \tag{A.23}$$

for $k' \in \{1,\ldots,K\} \setminus \{k\}$. We may express (20) as

$$\widehat{\mathbf{u}}^{(k)} = \underset{\mathbf{u}\in\mathbb{R}^p}{\operatorname{argmin}} \sum_{k'\neq k} \frac{N_{k'}}{N-N_k} m^{(k,k')}\left(\mathbf{u}; \widehat{\boldsymbol{\beta}}^{(k,k')}\right) + \lambda_{\mathsf{u}}\|\mathbf{u}\|_1,$$

Similar to the proof of Theorem 1, we establish the estimation rate for $\widehat{\mathbf{u}}$ through an oracle inequality,

Lemma 13 *Under Assumptions 1, 2, we establish On event*

$$\Omega^{(k)} = \bigcap_{k'\neq k} \left\{\Delta^\intercal \ddot{\mathbb{m}}^{(k,k')}\left(\widehat{\boldsymbol{\beta}}^{(k,k')}\right)\Delta \ge \kappa^*_{\mathsf{rsc},1}\|\Delta\|_2\{\|\Delta\|_2 - \kappa^*_{\mathsf{rsc},2}\sqrt{\log(p)/N_{k'}}\|\Delta\|_1\}, \forall \|\Delta\|_2 \le 1\right\},$$

setting $\lambda_{\mathsf{u}} \asymp \sqrt{\log(p)/N}$ *such that*

$$\begin{aligned}\lambda_{\mathsf{u}} \ge 3\sum_{k'\neq k}\frac{N_{k'}}{N-N_k}\Bigg\{&\left\|\dot{\mathbf{m}}^{(k,k')}\left(\mathbf{u}_0;\widehat{\boldsymbol{\beta}}^{(k,k')}\right) - \mathbb{E}\left\{\dot{\mathbf{m}}^{(k,k')}\left(\mathbf{u}_0;\widehat{\boldsymbol{\beta}}^{(k,k')}\right) \mid \mathscr{D}^c_{k'}\right\}\right\|_\infty \\ &+ \kappa^*_{\mathsf{rsc},1}\kappa^*_{\mathsf{rsc},2}\sqrt{\log(p)/N_{k'}}\Bigg\},\end{aligned}$$

we have the oracle inequality for estimation error of $\widehat{\boldsymbol{\beta}}$,

$$\begin{aligned}\|\widehat{\mathbf{u}}^{(k)} - \mathbf{u}_0\|_2 &\le \max\left\{14\sqrt{s_{\mathsf{u}}}\lambda_{\mathsf{u}}/\kappa^*_{\mathsf{rsc},1}, 7M\sigma^3_{\mathsf{max}}\|\mathbf{u}_0\|_2 \sup_{k'\neq k}\left\|\boldsymbol{\beta}_0 - \widehat{\boldsymbol{\beta}}^{(k,k')}\right\|_2/\kappa^*_{\mathsf{rsc},1}\right\},\\ \|\widehat{\mathbf{u}}^{(k)} - \mathbf{u}_0\|_1 &\le \max\left\{84 s_{\mathsf{u}}\lambda_{\mathsf{u}}/\kappa^*_{\mathsf{rsc},1}, 21M\sigma^6_{\mathsf{max}}\|\mathbf{u}_0\|_2^2 \sup_{k'\neq k}\left\|\boldsymbol{\beta}_0 - \widehat{\boldsymbol{\beta}}^{(k,k')}\right\|_2^2/(\kappa^*_{\mathsf{rsc},1}\lambda_{\mathsf{u}})\right\}.\end{aligned}$$

The constants $\kappa^*_{\mathsf{rsc},1}, \kappa^*_{\mathsf{rsc},2}$ *are the restrictive strong convexity parameters specified in Lemma 20.*

The proof of Lemma 13 repeats the proof of the oracle inequality for Theorem 1, so we put the detail to Section C.

To use Lemma 13 for the estimation rate of $\widehat{\mathbf{u}}$, we only need to verify two conditions. First, the event $\Omega^{(k)}$ occurs with probability tending to one. Second, the oracle choice of λ_{u} is of order $\sqrt{\log(p)/N}$.

Repeating Theorem 1 for each $\widehat{\boldsymbol{\beta}}^{(k,k')}$, we have under (28)

$$\left\|\widehat{\boldsymbol{\beta}}^{(k,k')} - \boldsymbol{\beta}_0\right\|_2 = o_p(1).$$

Then by Lemma 20, the sets whose intersection forms $\Omega^{(k)}$ each occurs with probability tending to one. Since we set the number of fold finite $K \leq 10$, we can take union bound to obtain that $\Omega^{(k)}$ occurs with probability tending to one.

We may write

$$\begin{aligned}
&\dot{\mathbf{m}}^{(k,k')}\left(\mathbf{u}_0; \widehat{\boldsymbol{\beta}}^{(k,k')}\right) - \mathbb{E}\left\{\dot{\mathbf{m}}^{(k)}\left(\mathbf{u}_0; \widehat{\boldsymbol{\beta}}^{(k,k')}\right) \mid \mathscr{D}_{k'}^c\right\} \\
=&\frac{1}{N_{k'}} \sum_{i \in \mathcal{I}_{k'} \cup \mathcal{J}_{k'}} g'(\widehat{\boldsymbol{\beta}}^{(k,k')\mathsf{T}} \mathbf{X}_i)\mathbf{X}_i \mathbf{X}_i^{\mathsf{T}} \mathbf{u}_0 - \mathbb{E}_{i \in \mathcal{I}_{k'} \cup \mathcal{J}_{k'}} \left\{g'(\widehat{\boldsymbol{\beta}}^{(k,k')\mathsf{T}} \mathbf{X}_i)\mathbf{X}_i \mathbf{X}_i^{\mathsf{T}} \mathbf{u}_0 \mid \mathscr{D}_{k'}^c\right\}. \qquad \text{(A.24)}
\end{aligned}$$

Each element in (A.24) is an empirical process. Under Assumptions 1b and 2a, we can show that each summand is a sub-exponential random variable by Lemma 17-e, 17-f,

$$\left\|g'(\widehat{\boldsymbol{\beta}}^{(k,k')\mathsf{T}} \mathbf{X}_i) X_{i,j} \mathbf{X}_i^{\mathsf{T}} \mathbf{u}_0\right\|_{\psi_1} \leq M \|X_{i,j}\|_{\psi_2} \|\mathbf{X}_i^{\mathsf{T}} \mathbf{u}_0\|_{\psi_2} \leq M \sigma_{\mathsf{max}}^2 \|\mathbf{u}_0\|_2 / 2.$$

Hence, we can apply the Bernstein's inequality to show that

$$\left\|\dot{\mathbf{m}}^{(k,k')}\left(\mathbf{u}_0; \widehat{\boldsymbol{\beta}}^{(k,k')}\right) - \mathbb{E}\left\{\dot{\mathbf{m}}^{(k,k')}\left(\mathbf{u}_0; \widehat{\boldsymbol{\beta}}^{(k,k')}\right) \mid \mathscr{D}_{k'}^c\right\}\right\|_\infty = O_p\left(\sqrt{\log(p)/N_{k'}}\right).$$

Using the fact that $N_{k'} \asymp N$, we obtain that the oracle λ_{u} is of order $O_p\left(\sqrt{\log(p)/N}\right)$.

Therefore, we can apply Lemma 13 to obtain

$$\begin{aligned}
\|\widehat{\mathbf{u}}^{(k)} - \mathbf{u}_0\|_2 =& O_p\left(\sqrt{s_{\mathsf{u}} \log(p)/N} + \sup_{k' \neq k} \left\|\widehat{\boldsymbol{\beta}}^{(k,k')} - \boldsymbol{\beta}_0\right\|_2\right) \\
=& O_p\left(\sqrt{(s_{\boldsymbol{\beta}} + s_{\mathsf{u}}) \log(p)/N} + (1-\rho)\sqrt{s_{\boldsymbol{\gamma}} \log(p+q)/n}\right).
\end{aligned}$$

Part 2: Asymptotic approximation

Under Assumption 2b-i, we also have the tightness of $\|\widehat{\mathbf{u}}^{(k)}\|_2$ from the bound of $\|\mathbf{u}_0\|_2$

$$\|\mathbf{u}_0\|_2 \leq \|\boldsymbol{\Sigma}_0^{-1}\|_2 \|\mathbf{x}_{\text{std}}\|_2 \leq \sigma_{\mathsf{min}}^{-2}, \ \|\widehat{\mathbf{u}}^{(k)}\|_2 \leq \|\mathbf{u}_0\|_2 + \|\widehat{\mathbf{u}}^{(k)} - \mathbf{u}_0\|_2 = O_p(1). \qquad \text{(A.25)}$$

Define the scores of in-fold data as

$$\dot{\boldsymbol{\ell}}^{\dagger(k)}(\boldsymbol{\beta}; \boldsymbol{\gamma}) = \frac{1}{N_k}\left[\sum_{i \in \mathcal{J}_k} \mathbf{X}_i \{g(\boldsymbol{\beta}^{\mathsf{T}} \mathbf{X}_i) - g(\boldsymbol{\gamma}^{\mathsf{T}} \mathbf{W}_i)\} + \sum_{i \in \mathcal{I}_k} \mathbf{X}_i \{g(\boldsymbol{\beta}^{\mathsf{T}} \mathbf{X}_i) - Y_i\}\right],$$

$$\dot{\boldsymbol{\ell}}^{(k)}_{\mathsf{imp}}(\boldsymbol{\gamma}) = \frac{1}{n_k}\sum_{i\in\mathcal{I}_k}\mathbf{X}_i\{g(\boldsymbol{\gamma}^\intercal\mathbf{W}_i) - Y_i\}. \tag{A.26}$$

Since $\widehat{\mathbf{x}_{\mathrm{std}}^\intercal\boldsymbol{\beta}}$ is the average over K (at most 10) cross-fitted estimators, it suffices to study one of the cross-fitted estimators,

$$\widehat{\mathbf{x}_{\mathrm{std}}^\intercal\boldsymbol{\beta}}^{(k)} = \mathbf{x}_{\mathrm{std}}^\intercal\widehat{\boldsymbol{\beta}}^{(k)} - \dot{\boldsymbol{\ell}}^{\dagger(k)}(\widehat{\boldsymbol{\beta}}^{(k)};\widehat{\boldsymbol{\gamma}}^{(k)}) - (1-\rho)\dot{\boldsymbol{\ell}}^{(k)}_{\mathsf{imp}}(\widehat{\boldsymbol{\gamma}}^{(k)}),\ \widehat{\mathbf{x}_{\mathrm{std}}^\intercal\boldsymbol{\beta}} = \frac{1}{K}\sum_{k=1}^{K}\widehat{\mathbf{x}_{\mathrm{std}}^\intercal\boldsymbol{\beta}}^{(k)}. \tag{A.27}$$

We denote the expected Hessian matrices of losses in (A.26) as

$$\begin{aligned}\mathbb{H}(\boldsymbol{\beta}) = \mathbb{E}\left\{g'(\boldsymbol{\beta}^\intercal\mathbf{X}_i)\mathbf{X}_i\mathbf{X}_i^\intercal\right\},\ \boldsymbol{\Sigma}_0 = \mathbb{H}(\boldsymbol{\beta}_0),\\ \mathbb{H}_{\mathsf{imp}}(\boldsymbol{\gamma}) = \mathbb{E}\left\{g'(\boldsymbol{\gamma}^\intercal\mathbf{W}_i)\mathbf{X}_i\mathbf{W}_i^\intercal\right\},\ \boldsymbol{\Sigma}_{\mathsf{imp}} = \mathbb{H}_{\mathsf{imp}}(\boldsymbol{\gamma}_0).\end{aligned} \tag{A.28}$$

Our analysis of the approximation error is based on the first order Mean Value Theorem identity,

$$\begin{aligned}&\mathbb{E}\{\dot{\boldsymbol{\ell}}^{\dagger(k)}(\widehat{\boldsymbol{\beta}}^{(k)};\widehat{\boldsymbol{\gamma}}^{(k)}) \mid \mathscr{D}_k^c\} + (1-\rho)\mathbb{E}\{\dot{\boldsymbol{\ell}}^{(k)}_{\mathsf{imp}}(\widehat{\boldsymbol{\gamma}}^{(k)}) \mid \mathscr{D}_k^c\}\\ =&\underbrace{\mathbb{E}\{\dot{\boldsymbol{\ell}}^{\dagger(k)}(\boldsymbol{\beta}_0;\boldsymbol{\gamma}_0)\}}_{=0} + \mathbb{H}(\widetilde{\boldsymbol{\beta}})\{\widehat{\boldsymbol{\beta}}^{(k)} - \boldsymbol{\beta}_0\} - (1-\rho)\mathbb{H}_{\mathsf{imp}}(\widetilde{\boldsymbol{\gamma}})\{\widehat{\boldsymbol{\gamma}}^{(k)} - \boldsymbol{\gamma}_0\}\\ &+ (1-\rho)\underbrace{\mathbb{E}\{\dot{\boldsymbol{\ell}}^{(k)}_{\mathsf{imp}}(\boldsymbol{\gamma}_0)\}}_{=0} + (1-\rho)\mathbb{H}_{\mathsf{imp}}(\widetilde{\boldsymbol{\gamma}})\{\widehat{\boldsymbol{\gamma}}^{(k)} - \boldsymbol{\gamma}_0\}\\ =&\mathbb{H}(\widetilde{\boldsymbol{\beta}})\{\widehat{\boldsymbol{\beta}}^{(k)} - \boldsymbol{\beta}_0\}\end{aligned} \tag{A.29}$$

for some $\widetilde{\boldsymbol{\beta}}$ on the path from $\bar{\boldsymbol{\beta}}(\widehat{\boldsymbol{\gamma}})$ to $\boldsymbol{\beta}_0$ and some $\widetilde{\boldsymbol{\gamma}}$ on the path from $\widehat{\boldsymbol{\gamma}}$ to $\boldsymbol{\gamma}_0$. The conditional expectation notation is declared at Definition 11. Based on (A.29), we analyze the approximation error for $\sqrt{n}\left(\widehat{\mathbf{x}_{\mathrm{std}}^\intercal\boldsymbol{\beta}}^{(k)} - \mathbf{x}_{\mathrm{std}}^\intercal\boldsymbol{\beta}_0\right)$ through the following decomposition,

$$\begin{aligned}&\sqrt{n}\left(\widehat{\mathbf{x}_{\mathrm{std}}^\intercal\boldsymbol{\beta}}^{(k)} - \sqrt{n}\mathbf{x}_{\mathrm{std}}^\intercal\boldsymbol{\beta}_0\right) + \sqrt{n}\mathbf{u}_0^\intercal\dot{\boldsymbol{\ell}}^{\dagger(k)}(\boldsymbol{\beta}_0;\boldsymbol{\gamma}_0) + \sqrt{n}(1-\rho)\mathbf{u}_0^\intercal\dot{\boldsymbol{\ell}}^{(k)}_{\mathsf{imp}}(\boldsymbol{\gamma}_0)\\ =&\sqrt{n}\mathbf{x}_{\mathrm{std}}^\intercal(\widehat{\boldsymbol{\beta}}^{(k)} - \boldsymbol{\beta}_0) - \sqrt{n}\widehat{\mathbf{u}}^{(k)\intercal}\dot{\boldsymbol{\ell}}^{\dagger(k)}(\widehat{\boldsymbol{\beta}}^{(k)};\widehat{\boldsymbol{\gamma}}^{(k)}) - \sqrt{n}(1-\rho)\widehat{\mathbf{u}}^{(k)\intercal}\dot{\boldsymbol{\ell}}^{(k)}_{\mathsf{imp}}(\widehat{\boldsymbol{\gamma}}^{(k)})\\ &+ \sqrt{n}\mathbf{u}_0^\intercal\dot{\boldsymbol{\ell}}^{\dagger(k)}(\boldsymbol{\beta}_0;\boldsymbol{\gamma}_0) + (1-\rho)\mathbf{u}_0^\intercal\dot{\boldsymbol{\ell}}^{(k)}_{\mathsf{imp}}(\boldsymbol{\gamma}_0) + \sqrt{n}\widehat{\mathbf{u}}^{(k)\intercal}\mathbb{E}\{\dot{\boldsymbol{\ell}}^{\dagger(k)}(\widehat{\boldsymbol{\beta}}^{(k)};\widehat{\boldsymbol{\gamma}}^{(k)}) \mid \mathscr{D}_k^c\}\\ &+ \sqrt{n}(1-\rho)\widehat{\mathbf{u}}^{(k)\intercal}\mathbb{E}\{\dot{\boldsymbol{\ell}}^{(k)}_{\mathsf{imp}}(\widehat{\boldsymbol{\gamma}}^{(k)}) \mid \mathscr{D}_k^c\} - \sqrt{n}\widehat{\mathbf{u}}^{(k)\intercal}\mathbb{H}(\widetilde{\boldsymbol{\beta}})\{\widehat{\boldsymbol{\beta}}^{(k)} - \boldsymbol{\beta}_0\}\\ =&\underbrace{\sqrt{n}\left\{\mathbf{x}_{\mathrm{std}} - \mathbb{H}(\widetilde{\boldsymbol{\beta}})\mathbf{u}_0\right\}^\intercal(\widehat{\boldsymbol{\beta}}^{(k)} - \boldsymbol{\beta}_0)}_{T_1} + \underbrace{\sqrt{n}\left(\mathbf{u}_0 - \widehat{\mathbf{u}}^{(k)}\right)^\intercal\mathbb{H}(\widetilde{\boldsymbol{\beta}})(\widehat{\boldsymbol{\beta}}^{(k)} - \boldsymbol{\beta}_0)}_{T_2}\\ &+ \underbrace{\sqrt{n}\widehat{\mathbf{u}}^{(k)\intercal}\left[\mathbb{E}\{\dot{\boldsymbol{\ell}}^{\dagger(k)}(\widehat{\boldsymbol{\beta}}^{(k)};\widehat{\boldsymbol{\gamma}}^{(k)}) \mid \mathscr{D}_k^c\} - \left\{\dot{\boldsymbol{\ell}}^{\dagger(k)}(\widehat{\boldsymbol{\beta}}^{(k)};\widehat{\boldsymbol{\gamma}}^{(k)}) - \dot{\boldsymbol{\ell}}^{\dagger(k)}(\boldsymbol{\beta}_0;\boldsymbol{\gamma}_0)\right\}\right]}_{T_3}\\ &+ \underbrace{\sqrt{n}(1-\rho)\widehat{\mathbf{u}}^{(k)\intercal}\left[\mathbb{E}\{\dot{\boldsymbol{\ell}}^{(k)}_{\mathsf{imp}}(\widehat{\boldsymbol{\gamma}}^{(k)}) \mid \mathscr{D}_k^c\} - \left\{\dot{\boldsymbol{\ell}}^{(k)}_{\mathsf{imp}}(\widehat{\boldsymbol{\gamma}}^{(k)}) - \dot{\boldsymbol{\ell}}^{(k)}_{\mathsf{imp}}(\boldsymbol{\gamma}_0)\right\}\right]}_{T_4}\end{aligned}$$

$$+ \underbrace{\sqrt{n}\left(\mathbf{u}_0 - \widehat{\mathbf{u}}^{(k)}\right)^{\mathsf{T}} \left\{\dot{\boldsymbol{\ell}}^{\dagger(k)}(\boldsymbol{\beta}_0; \boldsymbol{\gamma}_0) + (1-\rho)\dot{\boldsymbol{\ell}}^{(k)}_{\mathsf{imp}}(\boldsymbol{\gamma}_0)\right\}}_{T_5} \tag{A.30}$$

Here we state the rates for T_1-T_5,

$$T_1 = O_p\left(\sqrt{n}\|\widehat{\boldsymbol{\beta}}^{(k)} - \boldsymbol{\beta}_0\|_2^2\right),\ T_2 = O_p\left(\sqrt{n}\|\widehat{\mathbf{u}}^{(k)} - \mathbf{u}_0\|_2\|\widehat{\boldsymbol{\beta}}^{(k)} - \boldsymbol{\beta}_0\|_2\right),$$
$$T_3 = O_p\left(\rho\|\widehat{\boldsymbol{\beta}}^{(k)} - \boldsymbol{\beta}_0\|_2 + \sqrt{\rho(1-\rho)}\|\widehat{\boldsymbol{\gamma}}^{(k)} - \boldsymbol{\gamma}_0\|_2\right),$$
$$T_4 = O_p\left((1-\rho)\|\widehat{\boldsymbol{\gamma}}^{(k)} - \boldsymbol{\gamma}_0\|_2\right),\ T_5 = O_p\left(\|\widehat{\mathbf{u}}^{(k)} - \mathbf{u}_0\|_2\right).$$

With the assumed estimation rate in (A.22), we have

$$T_1 + T_2 + T_3 + T_4 + T_5 = o_p(1).$$

Thus, we have shown

$$\begin{aligned}\sqrt{n}\left(\widehat{\mathbf{x}_{\text{std}}^{\mathsf{T}}\boldsymbol{\beta}} - \mathbf{x}_{\text{std}}^{\mathsf{T}}\boldsymbol{\beta}_0\right) =& \frac{1}{K}\sum_{k=1}^{K}\sqrt{n}\left(\widehat{\mathbf{x}_{\text{std}}^{\mathsf{T}}\boldsymbol{\beta}} - \mathbf{x}_{\text{std}}^{\mathsf{T}}\boldsymbol{\beta}_0\right)\\ =& \frac{1}{K}\sum_{k=1}^{K} -\sqrt{n}\mathbf{u}_0^{\mathsf{T}}\dot{\boldsymbol{\ell}}^{\dagger(k)}(\boldsymbol{\beta}_0;\boldsymbol{\gamma}_0) - \sqrt{n}(1-\rho)\mathbf{u}_0^{\mathsf{T}}\dot{\boldsymbol{\ell}}^{(k)}_{\mathsf{imp}}(\boldsymbol{\gamma}_0) + o_p(1)\\ =& -\sqrt{n}\mathbf{u}_0^{\mathsf{T}}\dot{\boldsymbol{\ell}}^{\dagger}(\boldsymbol{\beta}_0;\boldsymbol{\gamma}_0) - \sqrt{n}(1-\rho)\mathbf{u}_0^{\mathsf{T}}\dot{\boldsymbol{\ell}}_{\mathsf{imp}}(\boldsymbol{\gamma}_0) + o_p(1).\end{aligned}$$

Using the indicator $R_i = I(i \le n)$, we can alternatively write

$$\widehat{\mathbf{x}_{\text{std}}^{\mathsf{T}}\boldsymbol{\beta}} - \mathbf{x}_{\text{std}}^{\mathsf{T}}\boldsymbol{\beta}_0 = \frac{1}{N}\sum_{i=1}^{N}\frac{R_i}{\rho}\mathbf{u}_0^{\mathsf{T}}\mathbf{X}_i\{Y_i - g(\boldsymbol{\gamma}_0^{\mathsf{T}}\mathbf{W}_i)\} - \mathbf{u}_0^{\mathsf{T}}\mathbf{X}_i\{g(\boldsymbol{\gamma}_0^{\mathsf{T}}\mathbf{W}_i) - g(\boldsymbol{\beta}_0^{\mathsf{T}}\mathbf{X}_i)\} + o_p\left((\rho n)^{-1/2}\right). \tag{A.31}$$

We provide the details of T_1-T_5 in Section C2.

Part 3: Variance estimation

Finally, we show that asymptotic variance V_{SAS} defined in (29) is bounded from infinity and zero with the consistent estimator $\widehat{V}_{\mathsf{SAS}}$ defined in (23).

By the Cauchy-Schwartz inequality, we have a bound for the variance

$$\begin{aligned}V_{\mathsf{SAS}} =& \mathbb{E}\left[(\mathbf{u}_0^{\mathsf{T}}\mathbf{X}_i)^2\{(1-\rho)\cdot g(\boldsymbol{\gamma}_0^{\mathsf{T}}\mathbf{W}_i) + \rho\cdot g(\boldsymbol{\beta}_0^{\mathsf{T}}\mathbf{X}_i) - Y_i\}^2\right]\\ &+ \rho(1-\rho)\mathbb{E}[(\mathbf{u}_0^{\mathsf{T}}\mathbf{X}_i)^2\{g(\boldsymbol{\gamma}_0^{\mathsf{T}}\mathbf{W}_i) - g(\boldsymbol{\beta}_0^{\mathsf{T}}\mathbf{X}_i)\}^2]\\ \le& \sqrt{\mathbb{E}\left[(\mathbf{u}_0^{\mathsf{T}}\mathbf{X}_i)^4\right]\mathbb{E}\left[\{(1-\rho)\cdot g(\boldsymbol{\gamma}_0^{\mathsf{T}}\mathbf{W}_i) + \rho\cdot g(\boldsymbol{\beta}_0^{\mathsf{T}}\mathbf{X}_i) - Y_i\}^4\right]}\\ &+ \rho(1-\rho)\sqrt{\mathbb{E}\left[(\mathbf{u}_0^{\mathsf{T}}\mathbf{X}_i)^4\right]\mathbb{E}\left[\{g(\boldsymbol{\gamma}_0^{\mathsf{T}}\mathbf{W}_i) - g(\boldsymbol{\beta}_0^{\mathsf{T}}\mathbf{X}_i)\}^4\right]}\end{aligned}$$

Under Assumptions 1a, 2a, we have the sub-Gaussian and sub-exponential variables

$$\|\mathbf{u}_0^{\mathsf{T}}\mathbf{X}_i\|_{\psi_2} \le \|\mathbf{u}_0\|_2\sigma_{\mathsf{max}}/\sqrt{2} \le \sigma_{\mathsf{min}}^{-2}\sigma_{\mathsf{max}}/\sqrt{2},$$

$$\|(1-\rho)\cdot g(\boldsymbol{\gamma}_0^{\intercal}\mathbf{W}_i)+\rho\cdot g(\boldsymbol{\beta}_0^{\intercal}\mathbf{X}_i)-Y_i\|_{\psi_2}\le 2(\nu_1\vee\nu_2),$$
$$\|g(\boldsymbol{\gamma}_0^{\intercal}\mathbf{W}_i)-g(\boldsymbol{\beta}_0^{\intercal}\mathbf{X}_i)\|_{\psi_2}\le 2\{\|g(\boldsymbol{\gamma}_0^{\intercal}\mathbf{W}_i)-Y_i\|_{\psi_2}\vee\|g(\boldsymbol{\beta}_0^{\intercal}\mathbf{X}_i)-Y_i\|_{\psi_2}\}\le 2(\nu_1\vee\nu_2).$$

By the bound for the moments of sub-Gaussian and sub-exponential random variables stated in Lemma 17-b, we have

$$V_{\mathsf{SAS}}\le 8\sqrt{2}\sigma_{\mathsf{min}}^{-4}\sigma_{\mathsf{max}}^{2}(\nu_1\vee\nu_2)^2.$$

Under Assumptions 1b, 2a, 2b-i and 2c, we have a lower bound for V_{SAS},

$$\begin{aligned}V_{\mathsf{SAS}}\ge&\mathbf{u}_0^{\intercal}\mathbb{E}[\mathbf{X}_i\mathbf{X}_i^{\intercal}\{(1-\rho)\cdot g(\boldsymbol{\gamma}_0^{\intercal}\mathbf{W}_i)+\rho\cdot g(\boldsymbol{\beta}_0^{\intercal}\mathbf{X}_i)-Y_i\}^2]\mathbf{u}_0\\ \ge&\|\mathbf{u}_0\|_2^2\sigma_{\mathsf{min}}^4\\ \ge&M^{-2}\sigma_{\mathsf{max}}^{-4}\sigma_{\mathsf{min}}^{4}\nu_3\|\mathbf{x}_{\mathrm{std}}\|_2^2,\end{aligned}$$

which is bounded away from zero.

We analyze the estimation error of variance $\widehat{V}_{\mathsf{SAS}}-V_{\mathsf{SAS}}$ through the decomposition,

$$\begin{aligned}&\widehat{V}_{\mathsf{SAS}}-V_{\mathsf{SAS}}\\ =&\left.\begin{aligned}&\sum_{k=1}^{K}\frac{n_k}{n}\Bigg(\frac{1}{n_k}\sum_{i\in\mathcal{I}_k}(\widehat{\mathbf{u}}^{(k)\intercal}\mathbf{X}_i)^2\{(1-\rho)\cdot g(\widehat{\boldsymbol{\gamma}}^{(k)\intercal}\mathbf{W}_i)+\rho\cdot g(\widehat{\boldsymbol{\beta}}^{(k)\intercal}\mathbf{X}_i)-Y_i\}^2\\ &\quad-\mathbb{E}_{i\in\mathcal{I}_k}\left[(\widehat{\mathbf{u}}^{(k)\intercal}\mathbf{X}_i)^2\{(1-\rho)\cdot g(\widehat{\boldsymbol{\gamma}}^{(k)\intercal}\mathbf{W}_i)+\rho\cdot g(\widehat{\boldsymbol{\beta}}^{(k)\intercal}\mathbf{X}_i)-Y_i\}^2\mid\mathscr{D}_k^c\right]\Bigg)\end{aligned}\right\}T_1'\\ &\left.\begin{aligned}+&\sum_{k=1}^{K}\frac{n_k}{n}\Bigg(\mathbb{E}_{i\in\mathcal{I}_k}\left[(\widehat{\mathbf{u}}^{(k)\intercal}\mathbf{X}_i)^2\{(1-\rho)\cdot g(\widehat{\boldsymbol{\gamma}}^{(k)\intercal}\mathbf{W}_i)+\rho\cdot g(\widehat{\boldsymbol{\beta}}^{(k)\intercal}\mathbf{X}_i)-Y_i\}^2\mid\mathscr{D}_k^c\right]\\ &\quad-\mathbb{E}\left[(\mathbf{u}_0^{\intercal}\mathbf{X}_i)^2\{(1-\rho)\cdot g(\boldsymbol{\gamma}_0^{\intercal}\mathbf{W}_i)+\rho\cdot g(\boldsymbol{\beta}_0^{\intercal}\mathbf{X}_i)-Y_i\}^2\right]\Bigg)\end{aligned}\right\}T_2'\\ &\left.\begin{aligned}+&\rho(1-\rho)\sum_{k=1}^{K}\frac{N_k-n_k}{N-n}\Bigg(\frac{K}{N_k-n_k}\sum_{i\in\mathcal{J}_k}(\widehat{\mathbf{u}}^{(k)\intercal}\mathbf{X}_i)^2\{g(\widehat{\boldsymbol{\beta}}^{(k)\intercal}\mathbf{X}_i)-g(\widehat{\boldsymbol{\gamma}}^{(k)\intercal}\mathbf{W}_i)\}^2\\ &\quad-\mathbb{E}_{i\in\mathcal{I}_k}\left[(\widehat{\mathbf{u}}^{(k)\intercal}\mathbf{X}_i)^2\{g(\widehat{\boldsymbol{\beta}}^{(k)\intercal}\mathbf{X}_i)-g(\widehat{\boldsymbol{\gamma}}^{(k)\intercal}\mathbf{W}_i)\}^2\mid\mathscr{D}_k^c\right]\Bigg)\end{aligned}\right\}T_3'\\ &\left.\begin{aligned}+&\rho(1-\rho)\sum_{k=1}^{K}\frac{N_k-n_k}{N-n}\Bigg(\mathbb{E}_{i\in\mathcal{I}_k}\left[(\widehat{\mathbf{u}}^{(k)\intercal}\mathbf{X}_i)^2\{g(\widehat{\boldsymbol{\beta}}^{(k)\intercal}\mathbf{X}_i)-g(\widehat{\boldsymbol{\gamma}}^{(k)\intercal}\mathbf{W}_i)\}^2\mid\mathscr{D}_k^c\right]\\ &\quad-\mathbb{E}\left[(\mathbf{u}_0^{\intercal}\mathbf{X}_i)^2\{g(\boldsymbol{\beta}_0^{\intercal}\mathbf{X}_i)-g(\boldsymbol{\gamma}_0^{\intercal}\mathbf{W}_i)\}^2\right]\Bigg)\end{aligned}\right\}T_4'\end{aligned}$$

Here we state the rates for T_1'-T_4',

$$T_1'=O_p\left(n^{-1/2}\right),\ T_2'=O_p\left(\|\widehat{\mathbf{u}}-\mathbf{u}_0\|_2+(1-\rho)\|\widehat{\boldsymbol{\gamma}}-\boldsymbol{\gamma}_0\|_2+\rho\|\widehat{\boldsymbol{\beta}}-\boldsymbol{\beta}_0\|_2\right),$$
$$T_3'=O_p\left(\rho(1-\rho)N^{-1/2}\right),\ T_4'=O_p\left(\rho(1-\rho)\left\{\|\widehat{\mathbf{u}}-\mathbf{u}_0\|_2+\|\widehat{\boldsymbol{\gamma}}-\boldsymbol{\gamma}_0\|_2+\|\widehat{\boldsymbol{\beta}}-\boldsymbol{\beta}_0\|_2\right\}\right).$$

With the assumed estimation rate in (A.22), we have

$$T_1' + T_2' + T_3' + T_4' = o_p(1).$$

We provide the details of T_1'-T_4' in Section C2.

Part 4: Conclusion with estimation rates

From the approximation in Part 2 and the boundedness and non-degeneracy of V_{SAS} in Part 3, we have shown the asymptotic normality of the cross-fitted debiased estimator

$$\sqrt{n} V_{\mathsf{SAS}}^{-1/2} \left(\widehat{\mathbf{x}_{\text{std}}^{\mathsf{T}} \boldsymbol{\beta}} - \mathbf{x}_{\text{std}}^{\mathsf{T}} \boldsymbol{\beta}_0 \right) \rightsquigarrow N(0,1).$$

Together with the consistency of $\widehat{V}_{\mathsf{SAS}}$ in Part 3, we have

$$\sqrt{n} \widehat{V}_{\mathsf{SAS}}^{-1/2} \left(\widehat{\mathbf{x}_{\text{std}}^{\mathsf{T}} \boldsymbol{\beta}} - \mathbf{x}_{\text{std}}^{\mathsf{T}} \boldsymbol{\beta}_0 \right) \rightsquigarrow N(0,1).$$

Part 5: Sufficient dimension condition

We have established the rate of estimation for $\widehat{\boldsymbol{\gamma}}$, $\widehat{\boldsymbol{\beta}}$ and $\widehat{\mathbf{u}}$ from Lemma 21, Theorem 1 and Part 4 of this proof above. Since we only keep one fold of the data away for the cross-fitted estimators, they follow the same rates of estimation,

$$\begin{aligned}
\|\widehat{\boldsymbol{\gamma}}^{(k)} - \boldsymbol{\gamma}_0\|_2 &= O_p\left(\sqrt{s_{\gamma} \log(p+q)/n}\right), \\
\|\widehat{\boldsymbol{\beta}}^{(k)} - \boldsymbol{\beta}_0\|_2 &= O_p\left(\sqrt{s_{\boldsymbol{\beta}} \log(p)/N} + (1-\rho)\sqrt{s_{\gamma} \log(p+q)/n}\right), \\
\|\widehat{\mathbf{u}}^{(k)} - \mathbf{u}_0\|_2 &= O_p\left(\sqrt{(s_{\boldsymbol{\beta}} + s_{\mathsf{u}}) \log(p)/N} + (1-\rho)\sqrt{s_{\gamma} \log(p+q)/n}\right).
\end{aligned}$$

Applying the rates of estimation, we show dimension assumption (28) is sufficient for (A.22).

B4 Efficiency of SAS Inference

Relative Efficiency to Supervised Learning

Proof [Proof of Proposition 9] We prove the Proposition by direct calculation

$$\begin{aligned}
& V_{\mathsf{SL}} - V_{\mathsf{SAS}} \\
=& \mathbb{E}[(\mathbf{u}_0^{\mathsf{T}} \mathbf{X}_i)^2 \{Y - g(\boldsymbol{\beta}_0^{\mathsf{T}} \mathbf{X}_i)\}^2] - \mathbb{E}[(\mathbf{u}_0^{\mathsf{T}} \mathbf{X}_i)^2 \{Y - (1-\rho) \cdot \mathbb{E}(Y|\mathbf{S}_i, \mathbf{X}_i) \rho \cdot g(\boldsymbol{\beta}_0^{\mathsf{T}} \mathbf{X}_i)\}^2] \\
=& \mathbb{E}[(\mathbf{u}_0^{\mathsf{T}} \mathbf{X}_i)^2 \{(1-\rho^2) g(\boldsymbol{\beta}_0^{\mathsf{T}} \mathbf{X}_i)^2 - 2(1-\rho) g(\boldsymbol{\beta}_0^{\mathsf{T}} \mathbf{X}_i) \mathbb{E}(Y|\mathbf{S}_i, \mathbf{X}_i) + (1-\rho^2) \mathbb{E}(Y|\mathbf{S}_i, \mathbf{X}_i)^2\}] \\
=& (1-\rho)^2 \mathbb{E}[(\mathbf{u}_0^{\mathsf{T}} \mathbf{X}_i)^2 \{\mathbb{E}(Y|\mathbf{S}_i, \mathbf{X}_i) - g(\boldsymbol{\beta}_0^{\mathsf{T}} \mathbf{X}_i)\}^2] \\
& + 2\rho(1-\rho) \mathbb{E}[(\mathbf{u}_0^{\mathsf{T}} \mathbf{X}_i)^2 \{\mathbb{E}(Y|\mathbf{S}_i, \mathbf{X}_i)^2 + g(\boldsymbol{\beta}_0^{\mathsf{T}} \mathbf{X}_i)^2\}].
\end{aligned}$$

The last expression is the sum of expectations of complete squares, so it must be non-negative. Thus, we have shown that the SAS asymptotic variance is no greater than the supervised learning variance. The equality holds only if 1) $\rho = 1$ all samples are labelled; 2) or $\rho = 0$ and $\mathbf{u}_0^{\mathsf{T}} \mathbf{X}_i \{\mathbb{E}(Y|\mathbf{S}_i, \mathbf{X}_i) - g(\boldsymbol{\beta}_0^{\mathsf{T}} \mathbf{X}_i)\} = 0$ almost surely. ■

Efficiency Bound among Semi-parametric RAL Estimators

Proof [Proof of Proposition 10] The proof follows the flow of Section D.2 in Kallus and Mao (2020). The semi-parametric model for the observed data is

$$\mathcal{M}_{\mathsf{obs}} = \Big\{ f_{\mathbf{X},Y,\mathbf{S},R}(\mathbf{x},y,\mathbf{s},r) = f_{\mathbf{X}}(\mathbf{x}) f_{\mathbf{S}|\mathbf{X}}(\mathbf{s}|\mathbf{x}) \left\{ \rho f_{Y|\mathbf{S},\mathbf{X}}(y|\mathbf{s},\mathbf{x}) \right\}^r (1-\rho)^{1-r} : \\ f_{\mathbf{X}}, f_{\mathbf{S}|\mathbf{X}}, f_{Y|\mathbf{S},\mathbf{X}} \text{ are arbitrary pdf/pmf}, \Big\}. \tag{A.32}$$

We consider the parametric sub-model

$$\mathcal{M}_{\mathsf{par}} = \Big\{ f_{\mathbf{X},Y,\mathbf{S},R}(\mathbf{x},y,\mathbf{s},r;\boldsymbol{\zeta}) = f_{\mathbf{X}}(\mathbf{x};\boldsymbol{\zeta}) f_{\mathbf{S}|\mathbf{X}}(\mathbf{s}|\mathbf{x};\boldsymbol{\zeta}) \left\{ \rho f_{Y|\mathbf{S},\mathbf{X}}(y|\mathbf{s},\mathbf{x};\boldsymbol{\zeta}) \right\}^r \\ \times (1-\rho)^{1-r} : \ \boldsymbol{\zeta} \in \mathbb{R}^d \Big\}. \tag{A.33}$$

The score vector of the parametric sub-model is

$$\begin{aligned} &\boldsymbol{\Psi}(\mathbf{X},Y,\mathbf{S},R) \\ =& \left.\frac{\partial \log\{f_{\mathbf{X},Y,\mathbf{S},R}(\mathbf{X},Y,\mathbf{S},R;\boldsymbol{\zeta})\}}{\partial \boldsymbol{\zeta}}\right|_{\boldsymbol{\zeta}=\boldsymbol{\zeta}_0} \\ =& \left.\frac{\partial \log\{f_{\mathbf{X}}(\mathbf{X};\boldsymbol{\zeta})\}}{\partial \boldsymbol{\zeta}}\right|_{\boldsymbol{\zeta}=\boldsymbol{\zeta}_0} + \left.\frac{\partial \log\{f_{\mathbf{S}|\mathbf{X}}(\mathbf{S}|\mathbf{X};\boldsymbol{\zeta})\}}{\partial \boldsymbol{\zeta}}\right|_{\boldsymbol{\zeta}=\boldsymbol{\zeta}_0} + R\left.\frac{\partial \log\{f_{Y|\mathbf{S},\mathbf{X}}(Y \mid \mathbf{S},\mathbf{X};\boldsymbol{\zeta})\}}{\partial \boldsymbol{\zeta}}\right|_{\boldsymbol{\zeta}=\boldsymbol{\zeta}_0} \\ =& \boldsymbol{\Psi}_{\mathbf{X}}(\mathbf{X}) + \boldsymbol{\Psi}_{\mathbf{S}}(\mathbf{S},\mathbf{X}) + R\boldsymbol{\Psi}_Y(Y,\mathbf{S},\mathbf{X}). \end{aligned} \tag{A.34}$$

Next, we decompose the the Hilbert space of mean zero finite variance random variables measurable to $\sigma\{\mathbf{X},\mathbf{S},R,YR\}$, denoted as $\mathcal{H}$. The model tangent space spanned by the score (A.34) is a linear sub-space of $\mathcal{H}$,

$$\begin{aligned} \Lambda &= \Lambda_{\mathbf{X}} \oplus \Lambda_{\mathbf{S}} \oplus \Lambda_Y, \\ \Lambda_{\mathbf{X}} &= \bigcup_{\mathcal{M}_{\mathsf{par}}} \operatorname{span}\{\boldsymbol{\Psi}_{\mathbf{X}}(\mathbf{X})\} = \{h(\mathbf{X}) \in \mathcal{H} : \mathbb{E}[h(\mathbf{X})] = 0\}, \\ \Lambda_{\mathbf{S}} &= \bigcup_{\mathcal{M}_{\mathsf{par}}} \operatorname{span}\{\boldsymbol{\Psi}_{\mathbf{S}}(\mathbf{S},\mathbf{X})\} = \{h(\mathbf{S},\mathbf{X}) \in \mathcal{H} : \mathbb{E}[h(\mathbf{S},\mathbf{X}) \mid \mathbf{X}] = 0\}, \\ \Lambda_{\mathbf{Y}} &= \bigcup_{\mathcal{M}_{\mathsf{par}}} \operatorname{span}\{R\boldsymbol{\Psi}_Y(Y,\mathbf{S},\mathbf{X})\} = \{Rh(Y,\mathbf{S},\mathbf{X}) \in \mathcal{H} : \mathbb{E}[h(Y,\mathbf{S},\mathbf{X}) \mid \mathbf{S},\mathbf{X}] = 0\}. \end{aligned} \tag{A.35}$$

The orthogonal space of model tangent space Λ is

$$\Lambda^{\perp} = \{h(R,\mathbf{S},\mathbf{X}) \in \mathcal{H} : \mathbb{E}[h(R,\mathbf{S},\mathbf{X}) \mid \mathbf{S},\mathbf{X}] = 0\}, \ \mathcal{H} = \Lambda \oplus \Lambda^{\perp}. \tag{A.36}$$

Now, we verify that the supervised learning influence function

$$\phi_{\mathsf{SL}}(\theta;\boldsymbol{\beta}) = \frac{R}{\rho}\mathbf{u}_0^{\mathsf{T}}\mathbf{X}\{Y - g(\boldsymbol{\beta}^{\mathsf{T}}\mathbf{X})\}$$

is indeed an influence function for $\mathbf{x}_{\rm std}^{\mathsf{T}}\boldsymbol{\beta}$ by showing

$$\mathbb{E}\{\phi_{\mathsf{SL}}(\theta_0;\boldsymbol{\beta}_0)\boldsymbol{\Psi}(\mathbf{X},Y,\mathbf{S},R)\} = \mathbf{x}_{\rm std}\frac{d}{d\boldsymbol{\zeta}}\boldsymbol{\beta}(\boldsymbol{\zeta})\bigg|_{\boldsymbol{\zeta}=\boldsymbol{\zeta}_0}.$$

Since $\boldsymbol{\beta}(\boldsymbol{\zeta})$ is an implicit function of $\boldsymbol{\zeta}$ through the moment condition

$$\mathbb{E}_{\boldsymbol{\zeta}}[\mathbf{X}\{g(\boldsymbol{\beta}(\boldsymbol{\zeta})^{\mathsf{T}}\mathbf{X}) - Y\} = 0,$$

we solve for its derivative by differentiating the moment condition

$$\begin{aligned}
\frac{d}{d\boldsymbol{\zeta}}\mathbb{E}_{\boldsymbol{\zeta}}[\mathbf{X}\{g(\boldsymbol{\beta}(\boldsymbol{\zeta})^{\mathsf{T}}\mathbf{X}) - Y\}]\bigg|_{\boldsymbol{\zeta}=\boldsymbol{\zeta}_0} &=0\\
\frac{d}{d\boldsymbol{\zeta}}\mathbb{E}_{\boldsymbol{\zeta}}[\mathbf{X}\{g(\boldsymbol{\beta}_0^{\mathsf{T}}\mathbf{X}) - Y\}]\bigg|_{\boldsymbol{\zeta}=\boldsymbol{\zeta}_0} + \mathbb{E}_{\boldsymbol{\zeta}_0}\{\mathbf{X}\mathbf{X}^{\mathsf{T}}g'(\boldsymbol{\beta}_0^{\mathsf{T}}\mathbf{X})\}\frac{d}{d\boldsymbol{\zeta}}\boldsymbol{\beta}(\boldsymbol{\zeta})\bigg|_{\boldsymbol{\zeta}=\boldsymbol{\zeta}_0} &=0\\
-\boldsymbol{\Theta}_0\mathbb{E}_{\boldsymbol{\zeta}_0}[\mathbf{X}\{g(\boldsymbol{\beta}_0^{\mathsf{T}}\mathbf{X}) - Y\}\{\boldsymbol{\Psi}_{\mathbf{X}}(\mathbf{X}) + \boldsymbol{\Psi}_{\mathbf{S}}(\mathbf{S},\mathbf{X}) + \boldsymbol{\Psi}_Y(Y,\mathbf{S},\mathbf{X})\}] &= \frac{d}{d\boldsymbol{\zeta}}\boldsymbol{\beta}(\boldsymbol{\zeta})\bigg|_{\boldsymbol{\zeta}=\boldsymbol{\zeta}_0}.
\end{aligned}$$

Then, we verify that the supervised learning influence function is valid

$$\begin{aligned}
\frac{d}{d\boldsymbol{\zeta}}\mathbf{x}_{\rm std}^{\mathsf{T}}\boldsymbol{\beta}(\boldsymbol{\zeta})\bigg|_{\boldsymbol{\zeta}=\boldsymbol{\zeta}_0} &= -\mathbb{E}_{\boldsymbol{\zeta}_0}\left[\frac{R}{\rho}\mathbf{u}_0^{\mathsf{T}}\mathbf{X}\{g(\boldsymbol{\beta}_0^{\mathsf{T}}\mathbf{X}) - Y\}\boldsymbol{\Psi}(\mathbf{X},Y,\mathbf{S},R)\right]\\
&=\mathbb{E}\{\phi_{\mathsf{SL}}(\theta_0;\boldsymbol{\beta}_0)\boldsymbol{\Psi}(\mathbf{X},Y,\mathbf{S},R)\}.
\end{aligned}$$

Finally, we derive the efficient influence function by subtract from ϕ_{SL} its projection onto $\Lambda^{\perp} = \Lambda_R$. Let $\Pi[h(\mathbf{D}) \mid \Lambda]$ be the projection of $h(\mathbf{D}) \in \mathcal{H}$ to the space Λ. We can easily calculate the projection of ϕ_{SL} onto Λ_R,

$$\begin{aligned}
\Pi[\phi_{\mathsf{SL}}(\theta_0;\boldsymbol{\beta}_0) \mid \Lambda_R] &=\mathbb{E}\{\phi_{\mathsf{SL}}(\theta_0;\boldsymbol{\beta}_0) \mid R,\mathbf{S},\mathbf{X}\} - \mathbb{E}\{\phi_{\mathsf{SL}}(\theta_0;\boldsymbol{\beta}_0) \mid \mathbf{S},\mathbf{X}\}\\
&=\frac{R}{\rho}\mathbf{u}_0^{\mathsf{T}}\mathbf{X}\{\mathbb{E}(Y \mid \mathbf{S},\mathbf{X}) - g(\boldsymbol{\beta}^{\mathsf{T}}\mathbf{X})\} - \mathbf{u}_0^{\mathsf{T}}\mathbf{X}\{\mathbb{E}(Y \mid \mathbf{S},\mathbf{X}) - g(\boldsymbol{\beta}^{\mathsf{T}}\mathbf{X})\}.
\end{aligned}$$

The efficient influence function is thus obtained

$$\begin{aligned}
\phi_{\mathsf{eff}}(\theta_0;\boldsymbol{\beta}_0) &=\phi_{\mathsf{SL}}(\theta_0;\boldsymbol{\beta}_0) - \Pi[\phi_{\mathsf{SL}}(\theta_0;\boldsymbol{\beta}_0) \mid \Lambda_R]\\
&=\frac{R}{\rho}\mathbf{u}_0^{\mathsf{T}}\mathbf{X}\{Y - g(\boldsymbol{\beta}^{\mathsf{T}}\mathbf{X})\} - \frac{R}{\rho}\mathbf{u}_0^{\mathsf{T}}\mathbf{X}\{\mathbb{E}(Y \mid \mathbf{S},\mathbf{X}) - g(\boldsymbol{\beta}^{\mathsf{T}}\mathbf{X})\}\\
&\quad + \mathbf{u}_0^{\mathsf{T}}\mathbf{X}\{\mathbb{E}(Y \mid \mathbf{S},\mathbf{X}) - g(\boldsymbol{\beta}^{\mathsf{T}}\mathbf{X})\}\\
&=\frac{R}{\rho}\mathbf{u}_0^{\mathsf{T}}\mathbf{X}\{Y - \mathbb{E}(Y \mid \mathbf{S},\mathbf{X})\} + \mathbf{u}_0^{\mathsf{T}}\mathbf{X}\{\mathbb{E}(Y \mid \mathbf{S},\mathbf{X}) - g(\boldsymbol{\beta}^{\mathsf{T}}\mathbf{X})\}.
\end{aligned}$$

■

Appendix C. Auxiliary Results

C1 General

Lemma 14 *Under Assumptions 1a, 1b, 2a, the residuals of the imputed loss are sub-Gaussian random variables ,*

$$\|g(\boldsymbol{\beta}^\intercal \mathbf{X}_i) - g(\boldsymbol{\gamma}^\intercal \mathbf{W}_i)\|_{\psi_2} \le 4\max\{\nu_1, \nu_2, M\|\boldsymbol{\beta}-\boldsymbol{\beta}_0\|_2\sigma_{\max}/\sqrt{2}, M\|\boldsymbol{\gamma}-\boldsymbol{\gamma}_0\|_2\sigma_{\max}/\sqrt{2}\}$$

Similarly,

$$\begin{aligned}
\|Y_i - g(\boldsymbol{\gamma}^\intercal \mathbf{W}_i)\|_{\psi_2} &\le 2\max\{\nu_1, M\|\boldsymbol{\gamma}-\boldsymbol{\gamma}_0\|_2\sigma_{\max}/\sqrt{2}\},\\
\|g(\boldsymbol{\gamma}^\intercal \mathbf{W}_i) - g(\boldsymbol{\gamma}_0^\intercal \mathbf{W}_i)\|_{\psi_2} &\le M\|\boldsymbol{\gamma}-\boldsymbol{\gamma}_0\|_2\sigma_{\max}/\sqrt{2},\\
\|g(\boldsymbol{\beta}^\intercal \mathbf{X}_i) - g(\boldsymbol{\beta}_0^\intercal \mathbf{X}_i)\|_{\psi_2} &\le M\|\boldsymbol{\beta}-\boldsymbol{\beta}_0\|_2\sigma_{\max}/\sqrt{2},\\
\|\rho\cdot g(\boldsymbol{\beta}^\intercal \mathbf{X}_i) + (1-\rho)\cdot g(\boldsymbol{\gamma}^\intercal \mathbf{W}_i) - Y\|_{\psi_2} &\le 4\max\{(1-\rho)\nu_1, \rho\nu_2,\\
&\qquad \rho M\|\boldsymbol{\beta}-\boldsymbol{\beta}_0\|_2\sigma_{\max}/\sqrt{2}, (1-\rho)M\|\boldsymbol{\gamma}-\boldsymbol{\gamma}_0\|_2\sigma_{\max}/\sqrt{2}\},\\
\|g(\boldsymbol{\beta}^\intercal \mathbf{X}_i) - g(\boldsymbol{\beta}_0^\intercal \mathbf{X}_i) - g(\boldsymbol{\gamma}^\intercal \mathbf{W}_i) + g(\boldsymbol{\gamma}_0^\intercal \mathbf{W}_i)\|_{\psi_2} &\le \sqrt{2}M\sigma_{\max}\max\{\|\boldsymbol{\beta}-\boldsymbol{\beta}_0\|_2, \|\boldsymbol{\gamma}-\boldsymbol{\gamma}_0\|_2\}.
\end{aligned}$$

Proof [Proof of Lemma 14] To establish the sub-exponential tail, we consider the following decomposition

$$\begin{aligned}
g(\boldsymbol{\beta}^\intercal \mathbf{X}_i) - g(\boldsymbol{\gamma}^\intercal \mathbf{W}_i) =& \{g(\boldsymbol{\beta}_0^\intercal \mathbf{X}_i) - Y_i\} - \{g(\boldsymbol{\gamma}_0^\intercal \mathbf{W}_i) - Y_i\}\\
&+ \{g(\boldsymbol{\beta}^\intercal \mathbf{X}_i) - g(\boldsymbol{\beta}_0^\intercal \mathbf{X}_i)\} - \{g(\boldsymbol{\gamma}^\intercal \mathbf{W}_i) - g(\boldsymbol{\gamma}_0^\intercal \mathbf{W}_i)\}.
\end{aligned} \tag{A.37}$$

According to Assumption 1a, the first two terms on the right-hand side of (A.37) are sub-Gaussian,

$$\|g(\boldsymbol{\beta}_0^\intercal \mathbf{X}_i) - Y_i\|_{\psi_2} \le \nu_1,\ \|g(\boldsymbol{\gamma}_0^\intercal \mathbf{W}_i) - Y_i\|_{\psi_2} \le \nu_2.$$

According to Assumption 1b, the latter two terms on the right-hand side of (A.37) are bounded by

$$|g(\boldsymbol{\beta}^\intercal \mathbf{X}_i) - g(\boldsymbol{\beta}_0^\intercal \mathbf{X}_i)| \le M|(\boldsymbol{\beta}-\boldsymbol{\beta}_0)^\intercal \mathbf{X}_i|,\ |g(\boldsymbol{\gamma}^\intercal \mathbf{W}_i) - g(\boldsymbol{\gamma}_0^\intercal \mathbf{W}_i)| \le M|(\boldsymbol{\gamma}-\boldsymbol{\gamma}_0)^\intercal \mathbf{W}_i|.$$

Under Assumption 2a, $(\boldsymbol{\beta}-\boldsymbol{\beta}_0)^\intercal \mathbf{X}_i$ and $(\boldsymbol{\gamma}-\boldsymbol{\gamma}_0)^\intercal \mathbf{W}_i$ are sub-Gaussian random variables,

$$\begin{aligned}
\|(\boldsymbol{\beta}-\boldsymbol{\beta}_0)^\intercal \mathbf{X}_i\|_{\psi_2} &\le \|\boldsymbol{\beta}-\boldsymbol{\beta}_0\|_2\sigma_{\max}/\sqrt{2}\\
\|(\boldsymbol{\gamma}-\boldsymbol{\gamma}_0)^\intercal \mathbf{W}_i\|_{\psi_2} &\le \|\boldsymbol{\gamma}-\boldsymbol{\gamma}_0\|_2\sigma_{\max}/\sqrt{2}.
\end{aligned}$$

By Lemma 17-e,

$$\begin{aligned}
\|g(\boldsymbol{\beta}^\intercal \mathbf{X}_i) - g(\boldsymbol{\beta}_0^\intercal \mathbf{X}_i)\|_{\psi_2} &\le M\|(\boldsymbol{\beta}-\boldsymbol{\beta}_0)^\intercal \mathbf{X}_i\|_{\psi_2} \le M\|\boldsymbol{\beta}-\boldsymbol{\beta}_0\|_2\sigma_{\max}/\sqrt{2}\\
\|g(\boldsymbol{\gamma}^\intercal \mathbf{W}_i) - g(\boldsymbol{\gamma}_0^\intercal \mathbf{W}_i)\|_{\psi_2} &\le \|(\boldsymbol{\gamma}-\boldsymbol{\gamma}_0)^\intercal \mathbf{W}_i\|_{\psi_2} \le M\|\boldsymbol{\gamma}-\boldsymbol{\gamma}_0\|_2\sigma_{\max}/\sqrt{2}.
\end{aligned}$$

Finally, we apply Lemma 17-d

$$\begin{aligned}
\|g(\boldsymbol{\beta}^\intercal \mathbf{X}_i) - g(\boldsymbol{\gamma}^\intercal \mathbf{W}_i)\|_{\psi_2} \le& 4\max\big\{\|g(\boldsymbol{\beta}_0^\intercal \mathbf{X}_i) - Y_i\|_{\psi_2}, \|g(\boldsymbol{\gamma}_0^\intercal \mathbf{W}_i) - Y_i\|_{\psi_2},\\
&\|g(\boldsymbol{\beta}^\intercal \mathbf{X}_i) - g(\boldsymbol{\beta}_0^\intercal \mathbf{X}_i)\|_{\psi_2}, \|g(\boldsymbol{\gamma}^\intercal \mathbf{W}_i) - g(\boldsymbol{\gamma}_0^\intercal \mathbf{W}_i)\|_{\psi_2}\big\}\\
\le& 4\max\left\{\nu_1, \nu_2, M\|\boldsymbol{\beta}-\boldsymbol{\beta}_0\|_2\sigma_{\max}/\sqrt{2}, M\|\boldsymbol{\gamma}-\boldsymbol{\gamma}_0\|_2\sigma_{\max}/\sqrt{2}\right\}.
\end{aligned}$$

Therefore, we have reached the conclusion.

We may obtain the rest of bounds following the same derivation. ■

C2 Inference

Analysis of Estimated Precision Matrix

Proof [Proof of Lemma 13]

The definition of the cross-fitted loss functions $m^{(k,k')}$ and their derivatives can be found at (A.23). By the definition of $\widehat{\mathbf{u}}^{(k)}$, we have

$$\sum_{k'\neq k} \frac{N_{k'}}{N-N_k} m^{(k,k')}\left(\widehat{\mathbf{u}}^{(k)};\widehat{\boldsymbol{\beta}}^{(k,k')}\right) + \lambda_{\mathsf{u}}\|\widehat{\mathbf{u}}^{(k)}\|_1 \leq \sum_{k'\neq k} \frac{N_{k'}}{N-N_k} m^{(k,k')}\left(\mathbf{u}_0;\widehat{\boldsymbol{\beta}}^{(k,k')}\right) + \lambda_{\mathsf{u}}\|\mathbf{u}_0\|_1.$$

Denote the standardized estimation error as $\boldsymbol{\delta} = (\widehat{\mathbf{u}}^{(k)} - \mathbf{u}_0)/\|\widehat{\mathbf{u}}^{(k)} - \mathbf{u}_0\|_2$. Due to convexity of the loss function, we have for $t = \|\widehat{\mathbf{u}}^{(k)} - \mathbf{u}_0\|_2 \wedge 1$

$$\sum_{k'\neq k} \frac{N_{k'}}{N-N_k} m^{(k,k')}\left(\mathbf{u}_0 + t\boldsymbol{\delta};\widehat{\boldsymbol{\beta}}^{(k,k')}\right) + \lambda_{\mathsf{u}}\|\mathbf{u}_0 + t\boldsymbol{\delta}\|_1 \leq \sum_{k'\neq k} \frac{N_{k'}}{N-N_k} m^{(k,k')}\left(\mathbf{u}_0;\widehat{\boldsymbol{\beta}}^{(k,k')}\right) + \lambda_{\mathsf{u}}\|\mathbf{u}_0\|_1. \tag{A.38}$$

By the triangle inequality $\|\mathbf{u}_0\|_1 - \|\mathbf{u}_0 + t\boldsymbol{\delta}\|_1 \leq t\|\boldsymbol{\delta}\|_1$, we have from (A.38)

$$\sum_{k'\neq k} \frac{N_{k'}}{N-N_k}\left\{ m^{(k,k')}\left(\mathbf{u}_0 + t\boldsymbol{\delta};\widehat{\boldsymbol{\beta}}^{(k,k')}\right) - m^{(k,k')}\left(\mathbf{u}_0;\widehat{\boldsymbol{\beta}}^{(k,k')}\right)\right\} \leq t\lambda_{\mathsf{u}}\|\boldsymbol{\delta}\|_1 \tag{A.39}$$

Because the loss functions $m^{(k)}$ are quadratic functions of $\mathbf{u}$, we can apply the restricted strong convexity event $\Omega^{(k)}$ to obtain

$$\begin{aligned}
& m^{(k,k')}\left(\mathbf{u}_0 + t\boldsymbol{\delta};\widehat{\boldsymbol{\beta}}^{(k,k')}\right) - m^{(k,k')}\left(\mathbf{u}_0;\widehat{\boldsymbol{\beta}}^{(k,k')}\right) - t\boldsymbol{\delta}^{\mathsf{T}}\dot{\mathbf{m}}^{(k,k')}\left(\mathbf{u}_0;\widehat{\boldsymbol{\beta}}^{(k,k')}\right) \\
=& t^2\boldsymbol{\delta}^{\mathsf{T}}\ddot{\mathbb{m}}^{(k,k')}\left(\widehat{\boldsymbol{\beta}}^{(k,k')}\right)\boldsymbol{\delta} \\
\geq& t^2\kappa^*_{\mathsf{rsc},1} - t\kappa^*_{\mathsf{rsc},1}\kappa^*_{\mathsf{rsc},2}\sqrt{\log(p)/N_{k'}}\|\boldsymbol{\delta}\|_1.
\end{aligned} \tag{A.40}$$

Applying (A.40) to (A.39), we have with large probability

$$\sum_{k'\neq k} \frac{N_{k'}}{N-N_k}\left\{ t\boldsymbol{\delta}^{\mathsf{T}}\dot{\mathbf{m}}^{(k,k')}\left(\mathbf{u}_0;\widehat{\boldsymbol{\beta}}^{(k,k')}\right) + t^2\kappa^*_{\mathsf{rsc},1} - t\kappa^*_{\mathsf{rsc},1}\kappa^*_{\mathsf{rsc},2}\sqrt{\log(p)/N_{k'}}\|\boldsymbol{\delta}\|_1\right\} \leq t\lambda_{\mathsf{u}}\|\boldsymbol{\delta}\|_1$$

where $\|\boldsymbol{\delta}\|_2 = 1$ from definition. Thus, we have reach

$$t\kappa^*_{\mathsf{rsc},1} \leq \lambda_{\mathsf{u}}\|\boldsymbol{\delta}\|_1 - \sum_{k'\neq k} \frac{N_{k'}}{N-N_k}\left\{ \boldsymbol{\delta}^{\mathsf{T}}\dot{\mathbf{m}}^{(k,k')}\left(\mathbf{u}_0;\widehat{\boldsymbol{\beta}}^{(k,k')}\right) - \kappa^*_{\mathsf{rsc},1}\kappa^*_{\mathsf{rsc},2}\sqrt{\log(p)/N_{k'}}\|\boldsymbol{\delta}\|_1\right\}. \tag{A.41}$$

The target parameter $\mathbf{u}_0$ can be identify by $\mathbb{E}\left\{\dot{\mathbf{m}}^{(k,k')}\left(\mathbf{u}_0;\widehat{\boldsymbol{\beta}}^{(k,k')}\right) \mid \mathscr{D}^c_{k'}\right\} = 0$. We use the fact to do a careful analysis of $\boldsymbol{\delta}^{\mathsf{T}}\dot{\mathbf{m}}^{(k,k')}\left(\mathbf{u}_0;\widehat{\boldsymbol{\beta}}^{(k,k')}\right)$ by the decomposition

$$\begin{aligned}
\left|\boldsymbol{\delta}^{\mathsf{T}}\dot{\mathbf{m}}^{(k,k')}\left(\mathbf{u}_0;\widehat{\boldsymbol{\beta}}^{(k,k')}\right)\right| =& \boldsymbol{\delta}^{\mathsf{T}}\left[\dot{\mathbf{m}}^{(k,k')}\left(\mathbf{u}_0;\widehat{\boldsymbol{\beta}}^{(k,k')}\right) - \mathbb{E}\left\{\dot{\mathbf{m}}^{(k,k')}\left(\mathbf{u}_0;\widehat{\boldsymbol{\beta}}^{(k,k')}\right) \mid \mathscr{D}^c_{k'}\right\}\right] \\
&+ \boldsymbol{\delta}^{\mathsf{T}}\left[\mathbb{E}\left\{\dot{\mathbf{m}}^{(k,k')}\left(\mathbf{u}_0;\widehat{\boldsymbol{\beta}}^{(k,k')}\right) \mid \mathscr{D}^c_{k'}\right\} - \mathbb{E}\left\{\dot{\mathbf{m}}^{(k,k')}\left(\mathbf{u}_0;\boldsymbol{\beta}_0\right) \mid \mathscr{D}^c_{k'}\right\}\right]
\end{aligned}$$

$$
\begin{aligned}
\leq & \|\boldsymbol{\delta}\|_1 \left\| \dot{\mathbf{m}}^{(k,k')}\left(\mathbf{u}_0; \widehat{\boldsymbol{\beta}}^{(k,k')}\right) - \mathbb{E}\left\{ \dot{\mathbf{m}}^{(k,k')}\left(\mathbf{u}_0; \widehat{\boldsymbol{\beta}}^{(k,k')}\right) \mid \mathscr{D}_{k'}^c \right\} \right\|_\infty \\
& + \left\| \mathbb{E}_{i\in\mathcal{I}_{k'}\cup\mathcal{J}_{k'}} [\mathbf{X}_i \mathbf{X}_i^\intercal \mathbf{u}_0 \{ g(\boldsymbol{\beta}_0^\intercal \mathbf{X}_i) - g(\widehat{\boldsymbol{\beta}}^{(k,k')\intercal} \mathbf{X}_i)\} \mid \mathscr{D}_{k'}^c] \right\|_2 . \quad \text{(A.42)}
\end{aligned}
$$

We establish the rate for L_2-norm of the population score at $\mathbf{u}_0$ through analyzing

$$
\sup_{\|\mathbf{v}\|_2=1} \mathbb{E}_{i\in\mathcal{I}_{k'}\cup\mathcal{J}_{k'}} [\mathbf{v}^\intercal \mathbf{X}_i \mathbf{X}_i^\intercal \mathbf{u}_0 \{ g(\boldsymbol{\beta}_0^\intercal \mathbf{X}_i) - g(\widehat{\boldsymbol{\beta}}^{(k,k')\intercal} \mathbf{X}_i)\} \mid \mathscr{D}_{k'}^c],
$$

whose bound can be derived from Assumptions 1b, 2a, 2a, the Cauchy-Schwartz inequality and Lemma 17-b,

$$
\begin{aligned}
& \mathbb{E}_{i\in\mathcal{I}_{k'}\cup\mathcal{J}_{k'}} [\mathbf{v}^\intercal \mathbf{X}_i \mathbf{X}_i^\intercal \mathbf{u}_0 \{ g(\boldsymbol{\beta}_0^\intercal \mathbf{X}_i) - g(\widehat{\boldsymbol{\beta}}^{(k,k')\intercal} \mathbf{X}_i)\} \mid \mathscr{D}_{k'}^c] \\
\leq & M \mathbb{E}_{i\in\mathcal{I}_{k'}\cup\mathcal{J}_{k'}} [|\mathbf{v}^\intercal \mathbf{X}_i \mathbf{X}_i^\intercal \mathbf{u}_0 \{ \left(\boldsymbol{\beta}_0 - \widehat{\boldsymbol{\beta}}^{(k,k')}\right)^\intercal \mathbf{X}_i \}| \mid \mathscr{D}_{k'}^c] \\
\leq & M \left[\mathbb{E}\{(\mathbf{v}^\intercal \mathbf{X}_i)^4\} \mathbb{E}\{(\mathbf{v}^\intercal \mathbf{X}_i)^4\} \right]^{1/4} \sqrt{ \mathbb{E}_{i\in\mathcal{I}_{k'}\cup\mathcal{J}_{k'}} \left[\left\{ \left(\boldsymbol{\beta}_0 - \widehat{\boldsymbol{\beta}}^{(k,k')}\right)^\intercal \mathbf{X}_i \right\}^2 \mid \mathscr{D}_{k'}^c \right] } \\
\leq & M \sigma_{\mathsf{max}}^3 \|\mathbf{v}\|_2 \|\mathbf{u}_0\|_2 \left\| \boldsymbol{\beta}_0 - \widehat{\boldsymbol{\beta}}^{(k,k')} \right\|_2 .
\end{aligned}
$$

Hence, we have shown

$$
\left\| \mathbb{E}_{i\in\mathcal{I}_{k'}\cup\mathcal{J}_{k'}} \left[\mathbf{X}_i \mathbf{X}_i^\intercal \mathbf{u}_0 \left\{ g(\boldsymbol{\beta}_0^\intercal \mathbf{X}_i) - g\left(\widehat{\boldsymbol{\beta}}^{(k,k')\intercal} \mathbf{X}_i\right) \right\} \mid \mathscr{D}_{k'}^c \right] \right\|_2 \leq M \sigma_{\mathsf{max}}^3 \|\mathbf{u}_0\|_2 \left\| \boldsymbol{\beta}_0 - \widehat{\boldsymbol{\beta}}^{(k,k')} \right\|_2 . \quad \text{(A.43)}
$$

By the bound for (A.42) through (A.43) and the definition of $\lambda_{\boldsymbol{\beta}}$, we have the bound from (A.41)

$$
t\kappa_{\mathsf{rsc},1}^* \leq 2\lambda_{\mathsf{u}} \|\boldsymbol{\delta}\|_1 + M\sigma_{\mathsf{max}}^3 \|\mathbf{u}_0\|_2 \sup_{k'\neq k} \left\| \boldsymbol{\beta}_0 - \widehat{\boldsymbol{\beta}}^{(k,k')} \right\|_2 . \quad \text{(A.44)}
$$

Hence, we can reach an immediate bound for estimation error from (A.44) without considering the sparsity of $\mathbf{u}_0$. We shall proceed to derive a sharper bound that involves the sparsity of $\mathbf{u}_0$. We separately analyze two cases.

Case 1:

$$
M\sigma_{\mathsf{max}}^3 \|\mathbf{u}_0\|_2 \sup_{k=1,\ldots,K} \left\| \boldsymbol{\beta}_0 - \widehat{\boldsymbol{\beta}}^{(k,k')} \right\|_2 \geq \|\boldsymbol{\delta}\|_1 \lambda_{\mathsf{u}}/3
$$

In this case, the estimation error is dominated by $\boldsymbol{\beta}_0 - \widehat{\boldsymbol{\beta}}^{(k,k')}$. We simply have from (A.44)

$$
\begin{aligned}
t\kappa_{\mathsf{rsc},1}^* &\leq 7M\sigma_{\mathsf{max}}^3 \|\mathbf{u}_0\|_2 \sup_{k=1,\ldots,K} \left\| \boldsymbol{\beta}_0 - \widehat{\boldsymbol{\beta}}^{(k,k')} \right\|_2 , \\
t\kappa_{\mathsf{rsc},1}^* \|\boldsymbol{\delta}\|_1 \lambda_{\mathsf{u}}/3 &\leq 7M\sigma_{\mathsf{max}}^6 \|\mathbf{u}_0\|_2^2 \sup_{k=1,\ldots,K} \left\| \boldsymbol{\beta}_0 - \widehat{\boldsymbol{\beta}}^{(k,k')} \right\|_2^2 .
\end{aligned}
$$

Thus, we have

$$
\|\widehat{\mathbf{u}}^{(k)} - \mathbf{u}_0\|_2 \leq 7M\sigma_{\mathsf{max}}^3 \|\mathbf{u}_0\|_2 \sup_{k'\neq k} \left\| \boldsymbol{\beta}_0 - \widehat{\boldsymbol{\beta}}^{(k,k')} \right\|_2 / \kappa_{\mathsf{rsc},1}^* ,
$$

$$\|\widehat{\mathbf{u}}^{(k)} - \mathbf{u}_0\|_1 \leq 21M\sigma_{\max}^6\|\mathbf{u}_0\|_2^2 \sup_{k'\neq k}\left\|\boldsymbol{\beta}_0 - \widehat{\boldsymbol{\beta}}^{(k,k')}\right\|_2^2/(\kappa_{\mathrm{rsc},1}^*\lambda_{\mathsf{u}}). \tag{A.45}$$

Case 2:

$$M\sigma_{\max}^3\|\mathbf{u}_0\|_2 \sup_{k'\neq k}\left\|\boldsymbol{\beta}_0 - \widehat{\boldsymbol{\beta}}^{(k,k')}\right\|_2 \leq \|\boldsymbol{\delta}\|_1\lambda_{\mathsf{u}}/3 \tag{A.46}$$

In this case, the estimation error is comparable to the situation that we have the true $\boldsymbol{\beta}_0$ for the Hessian. Thus, the sparsity of $\mathbf{u}_0$ may affect the estimation error.

Following the typical approach to establish the cone condition for $\boldsymbol{\delta}$, we analyze the symmetrized Bregman's divergence,

$$\begin{aligned}&(\widehat{\mathbf{u}}^{(k)} - \mathbf{u}_0)^\intercal \sum_{k'\neq k}\frac{N_{k'}}{N-N_k}\left\{\dot{\mathbf{m}}^{(k,k')}\left(\widehat{\mathbf{u}}^{(k)};\widehat{\boldsymbol{\beta}}^{(k,k')}\right) - \dot{\mathbf{m}}^{(k,k')}\left(\mathbf{u}_0;\widehat{\boldsymbol{\beta}}^{(k,k')}\right)\right\}\\ =&\|\widehat{\mathbf{u}}^{(k)} - \mathbf{u}_0\|_2\sum_{k'\neq k}\frac{N_{k'}}{N-N_k}\boldsymbol{\delta}^\intercal\left\{\dot{\mathbf{m}}^{(k,k')}\left(\widehat{\mathbf{u}}^{(k)};\widehat{\boldsymbol{\beta}}^{(k,k')}\right) - \dot{\mathbf{m}}^{(k,k')}\left(\mathbf{u}_0;\widehat{\boldsymbol{\beta}}^{(k,k')}\right)\right\}.\end{aligned} \tag{A.47}$$

Due to the convexity of the quadratic loss $m^{(k,k')}\left(\cdot;\widehat{\boldsymbol{\beta}}^{(k,k')}\right)$, the symmetrized Bregman's divergence (A.47) is nonnegative through a mean-value theorem,

$$\begin{aligned}&(\widehat{\mathbf{u}}^{(k)} - \mathbf{u}_0)^\intercal \sum_{k'\neq k}\frac{N_{k'}}{N-N_k}\left\{\dot{\mathbf{m}}^{(k,k')}\left(\widehat{\mathbf{u}}^{(k)};\widehat{\boldsymbol{\beta}}^{(k,k')}\right) - \dot{\mathbf{m}}^{(k,k')}\left(\mathbf{u}_0;\widehat{\boldsymbol{\beta}}^{(k,k')}\right)\right\}\\ =&\sum_{k'\neq k}\frac{N_{k'}}{N-N_k}\sum_{i\in\mathcal{I}_{k'}\cup\mathcal{J}_{k'}} g'(\widehat{\boldsymbol{\beta}}^{(k,k')\intercal}\mathbf{X}_i)\{(\widehat{\mathbf{u}}^{(k)} - \mathbf{u}_0)^\intercal\mathbf{X}_i\}^2\\ \geq&0.\end{aligned}$$

Denote the indices set of nonzero coefficient in $\mathbf{u}_0$ as $\mathcal{O}_{\mathsf{u}} = \{j : \mathbf{u}_{0,j} \neq 0\}$. We denote the $\boldsymbol{\delta}_{\mathcal{O}_{\mathsf{u}}}$ and $\boldsymbol{\delta}_{\mathcal{O}_{\mathsf{u}}^c}$ as the sub-vectors for $\boldsymbol{\delta}$ at positions in $\mathcal{O}_{\mathsf{u}}$ and at positions not in $\mathcal{O}_{\mathsf{u}}$, respectively. The solution $\widehat{\mathbf{u}}^{(k)}$ satisfies the KKT condition

$$\left\|\sum_{k'\neq k}\frac{N_{k'}}{N-N_k}\dot{\mathbf{m}}^{(k,k')}\left(\widehat{\mathbf{u}}^{(k)};\widehat{\boldsymbol{\beta}}^{(k,k')}\right)\right\|_\infty \leq \lambda_{\mathsf{u}},$$

$$\sum_{k'\neq k}\frac{N_{k'}}{N-N_k}\dot{m}^{(k,k')}\left(\widehat{\mathbf{u}}^{(k)};\widehat{\boldsymbol{\beta}}^{(k,k')}\right)_j = -\lambda_{\mathsf{u}}\,\mathrm{sign}(\widehat{u}_j^{(k)}),\ j:\widehat{u}_j^{(k)} \neq 0.$$

From the KKT condition and the definitions of $\boldsymbol{\delta}$ and $\mathcal{O}_{\mathsf{u}}$, we have

$$\delta_j\sum_{k'\neq k}\frac{N_{k'}}{N-N_k}\dot{m}^{(k,k')}\left(\widehat{\mathbf{u}}^{(k)};\widehat{\boldsymbol{\beta}}^{(k,k')}\right)_j \leq |\delta_j|\lambda_{\mathsf{u}},\ j\in\mathcal{O}_{\mathsf{u}};$$

$$\delta_j\sum_{k'\neq k}\frac{N_{k'}}{N-N_k}\dot{m}^{(k,k')}\left(\widehat{\mathbf{u}}^{(k)};\widehat{\boldsymbol{\beta}}^{(k,k')}\right)_j = \frac{-\widehat{u}_j^{(k)}\lambda_{\mathsf{u}}\,\mathrm{sign}(\widehat{u}_j^{(k)})}{\|\widehat{\mathbf{u}}^{(k)} - \mathbf{u}_0\|_2} = -\lambda_{\mathsf{u}}|\delta_j|,\ j\in\mathcal{O}_{\mathsf{u}}^c. \tag{A.48}$$

Applying the (A.48) to (A.47), we have the upper bound,

$$\boldsymbol{\delta}^\intercal\sum_{k'\neq k}\frac{N_{k'}}{N-N_k}\left\{\dot{\mathbf{m}}^{(k,k')}\left(\widehat{\mathbf{u}}^{(k)};\widehat{\boldsymbol{\beta}}^{(k,k')}\right) - \dot{\mathbf{m}}^{(k,k')}\left(\mathbf{u}_0;\widehat{\boldsymbol{\beta}}^{(k,k')}\right)\right\}$$

$$
\begin{aligned}
&= \sum_{j \in \mathcal{O}_{\mathsf{u}}} \delta_j \sum_{k' \neq k} \frac{N_{k'}}{N - N_k} \dot{m}^{(k,k')} \left(\widehat{\mathbf{u}}^{(k)}; \widehat{\boldsymbol{\beta}}^{(k,k')} \right)_j + \sum_{j \in \mathcal{O}_{\mathsf{u}}^{\mathsf{c}}} \delta_j \sum_{k' \neq k} \frac{N_{k'}}{N - N_k} \dot{m}^{(k,k')} \left(\widehat{\mathbf{u}}^{(k)}; \widehat{\boldsymbol{\beta}}^{(k,k')} \right)_j \\
&\quad + \sum_{k' \neq k} \frac{N_{k'}}{N - N_k} \boldsymbol{\delta}^{\mathsf{T}} \dot{\mathbf{m}}^{(k,k')} \left(\mathbf{u}_0; \widehat{\boldsymbol{\beta}}^{(k,k')} \right) \\
&\le \lambda_{\mathsf{u}} \sum_{j \in \mathcal{O}_{\mathsf{u}}} |\delta_j| - \lambda_{\mathsf{u}} \sum_{j \in \mathcal{O}_{\mathsf{u}}^{\mathsf{c}}} |\delta_j| + \boldsymbol{\delta}^{\mathsf{T}} \dot{\boldsymbol{\ell}}^{\dagger}(\boldsymbol{\beta}_0; \widehat{\gamma}) \\
&\le \lambda_{\mathsf{u}} \|\boldsymbol{\delta}_{\mathcal{O}_{\mathsf{u}}}\|_1 - \lambda_{\mathsf{u}} \|\boldsymbol{\delta}_{\mathcal{O}_{\mathsf{u}}^{\mathsf{c}}}\|_1 + \left| \sum_{k' \neq k} \frac{N_{k'}}{N - N_k} \boldsymbol{\delta}^{\mathsf{T}} \dot{\mathbf{m}}^{(k,k')} \left(\mathbf{u}_0; \widehat{\boldsymbol{\beta}}^{(k,k')} \right) \right|.
\end{aligned}
$$

Then, we apply (A.42), the definition of λ_{u} and (A.46),

$$
0 \le \lambda_{\mathsf{u}} \|\boldsymbol{\delta}_{\mathcal{O}_{\mathsf{u}}}\|_1 - \lambda_{\mathsf{u}} \|\boldsymbol{\delta}_{\mathcal{O}_{\mathsf{u}}^{\mathsf{c}}}\|_1 + \frac{2}{3} \lambda_{\mathsf{u}} \|\boldsymbol{\delta}\|_1, \quad \text{and} \quad \lambda_{\mathsf{u}} \|\boldsymbol{\delta}_{\mathcal{O}_{\mathsf{u}}^{\mathsf{c}}}\|_1 \le 5 \lambda_{\mathsf{u}} \|\boldsymbol{\delta}_{\mathcal{O}_{\mathsf{u}}}\|_1.
$$

Therefore, we can bound the L_1 norm of $\boldsymbol{\delta}$ by the cone property,

$$
\|\boldsymbol{\delta}\|_1 \le 6 \lambda_{\mathsf{u}} \|\boldsymbol{\delta}_{\mathcal{O}_{\mathsf{u}}}\|_1 \le 6 \sqrt{s_{\mathsf{u}}} \|\boldsymbol{\delta}\|_2 = 6 \sqrt{s_{\mathsf{u}}}. \tag{A.49}
$$

Now, we apply the cone condition (A.49) and the case condition (A.46) to the bound (A.44),

$$
t \kappa^*_{\mathsf{rsc},1} \le 14 \sqrt{s_{\mathsf{u}}} \lambda_{\mathsf{u}}, \; t \kappa^*_{\mathsf{rsc},1} \|\boldsymbol{\delta}\|_1 \le 84 s_{\mathsf{u}} \lambda_{\mathsf{u}}
$$

Thus, we obtain the rate for estimation error

$$
\|\widehat{\mathbf{u}}^{(k)} - \mathbf{u}_0\|_2 \le 14 \sqrt{s_{\mathsf{u}}} \lambda_{\mathsf{u}} / \kappa^*_{\mathsf{rsc},1}, \; \|\widehat{\mathbf{u}}^{(k)} - \mathbf{u}_0\|_1 \le 84 s_{\mathsf{u}} \lambda_{\mathsf{u}} / \kappa^*_{\mathsf{rsc},1}. \tag{A.50}
$$

Conclusion:

Since Case 1 and Case 2 are the complement of each other, one of them must occur. Thus, the bound of estimation error is controlled by the larger bound in the two cases,

$$
\|\widehat{\mathbf{u}}^{(k)} - \mathbf{u}_0\|_2 \le \max \left\{ 14 \sqrt{s_{\mathsf{u}}} \lambda_{\mathsf{u}} / \kappa^*_{\mathsf{rsc},1}, 7 M \sigma^3_{\mathsf{max}} \|\mathbf{u}_0\|_2 \sup_{k' \neq k} \left\| \boldsymbol{\beta}_0 - \widehat{\boldsymbol{\beta}}^{(k,k')} \right\|_2 / \kappa^*_{\mathsf{rsc},1} \right\},
$$

$$
\|\widehat{\mathbf{u}}^{(k)} - \mathbf{u}_0\|_1 \le \max \left\{ 84 s_{\mathsf{u}} \lambda_{\mathsf{u}} / \kappa^*_{\mathsf{rsc},1}, 21 M \sigma^6_{\mathsf{max}} \|\mathbf{u}_0\|_2^2 \sup_{k' \neq k} \left\| \boldsymbol{\beta}_0 - \widehat{\boldsymbol{\beta}}^{(k,k')} \right\|_2^2 / (\kappa^*_{\mathsf{rsc},1} \lambda_{\mathsf{u}}) \right\},
$$

which is our oracle inequality. ■

Analysis for Terms T_1-T_5 in Part 1

To show

$$
T_1 = \sqrt{n} \left\{ \mathbf{x}_{\mathrm{std}} - \mathbb{H}(\widetilde{\boldsymbol{\beta}}) \mathbf{u}_0 \right\} (\widehat{\boldsymbol{\beta}}^{(k)} - \boldsymbol{\beta}_0) = O_p \left(\sqrt{n} \|\widehat{\boldsymbol{\beta}}^{(k)} - \boldsymbol{\beta}_0\|_2^2 \right),
$$

we rewrite the term as a conditional expectation

$$
T_1 = \sqrt{n} \mathbf{u}_0^{\mathsf{T}} \left\{ \boldsymbol{\Sigma}_0 - \mathbb{H}(\widetilde{\boldsymbol{\beta}}) \right\} (\widehat{\boldsymbol{\beta}}^{(k)} - \boldsymbol{\beta}_0)
$$

$$=\sqrt{n}\mathbb{E}_{i\in\mathcal{J}_k}\left[\mathbf{u}_0^\intercal\mathbf{X}_i(\widehat{\boldsymbol{\beta}}^{(k)}-\boldsymbol{\beta}_0)^\intercal\mathbf{X}_i\{g'(\widetilde{\boldsymbol{\beta}}^\intercal\mathbf{X}_i)-g'(\widehat{\boldsymbol{\beta}}^{(k)\intercal}\mathbf{X}_i)\}\mid\mathscr{D}_k^c\right].$$

Under Assumptions 1b, 2a, we derive the bound for the expectation using the Cauchy-Schwartz inequality and Lemma 17-b,

$$\begin{aligned}|T_1|\leq&M\sqrt{n}\mathbb{E}_{i\in\mathcal{J}_k}\left[\mathbf{u}_0^\intercal\mathbf{X}_i\{(\widehat{\boldsymbol{\beta}}^{(k)}-\boldsymbol{\beta}_0)^\intercal\mathbf{X}_i\}^2\mid\mathscr{D}_k^c\right]\\ \leq&M\sqrt{n\mathbb{E}_{i\in\mathcal{J}_k}\left\{(\mathbf{u}_0^\intercal\mathbf{X}_i)^2\mid\mathscr{D}_k^c\right\}\mathbb{E}_{i\in\mathcal{J}_k}\left[\{(\widehat{\boldsymbol{\beta}}^{(k)}-\boldsymbol{\beta}_0)^\intercal\mathbf{X}_i\}^4\mid\mathscr{D}_k^c\right]}\\ \leq&M\sqrt{n8\|\mathbf{u}_0^\intercal\mathbf{X}_i\|_{\psi_2}^2\|(\widehat{\boldsymbol{\beta}}^{(k)}-\boldsymbol{\beta}_0)^\intercal\mathbf{X}_i\|_{\psi_2}^4}\\ \leq&\sqrt{n}M\|\mathbf{u}_0\|_2\left\|\widehat{\boldsymbol{\beta}}^{(k)}-\boldsymbol{\beta}_0\right\|_2^2\sigma_{\mathsf{max}}^3.\end{aligned}\tag{A.51}$$

Since $\|\mathbf{u}_0\|_2$ is bounded according to (A.25), we have established in

$$|T_1|=O_p\left(\sqrt{n}\|\widehat{\boldsymbol{\beta}}^{(k)}-\boldsymbol{\beta}_0\|_2^2\right)$$

as declared.

To show

$$T_2=\sqrt{n}\left(\mathbf{u}_0-\widehat{\mathbf{u}}^{(k)}\right)^\intercal\mathbb{H}(\widetilde{\boldsymbol{\beta}})(\widehat{\boldsymbol{\beta}}^{(k)}-\boldsymbol{\beta}_0)=O_p\left(\sqrt{n}\|\widehat{\boldsymbol{\beta}}^{(k)}-\boldsymbol{\beta}_0\|_2\|\widehat{\mathbf{u}}^{(k)}-\mathbf{u}_0\|_2\right),$$

we rewrite the term as a conditional expectation

$$T_2=\sqrt{n}\mathbb{E}_{i\in\mathcal{J}_k}\left[\left(\mathbf{u}_0-\widehat{\mathbf{u}}^{(k)}\right)^\intercal\mathbf{X}_i(\widehat{\boldsymbol{\beta}}^{(k)}-\boldsymbol{\beta}_0)^\intercal\mathbf{X}_ig'(\widetilde{\boldsymbol{\beta}}^\intercal\mathbf{X}_i)\mid\mathscr{D}_k^c\right].$$

Similar to (A.51), we derive the bound for the expectation under Assumptions 1b, 2a through the Cauchy-Schwartz inequality and Lemma 17-b, 17-f,

$$\begin{aligned}|T_2|\leq&M\sqrt{n}\mathbb{E}_{i\in\mathcal{J}_k}\left[|\left(\mathbf{u}_0-\widehat{\mathbf{u}}^{(k)}\right)^\intercal\mathbf{X}_i(\widehat{\boldsymbol{\beta}}^{(k)}-\boldsymbol{\beta}_0)^\intercal\mathbf{X}_i|\mid\mathscr{D}_k^c\right]\\ \leq&2M\sqrt{n}\|\left(\mathbf{u}_0-\widehat{\mathbf{u}}^{(k)}\right)^\intercal\mathbf{X}_i(\widehat{\boldsymbol{\beta}}^{(k)}-\boldsymbol{\beta}_0)^\intercal\mathbf{X}_i\|_{\psi_1}\\ \leq&2M\sqrt{n}\|\left(\mathbf{u}_0-\widehat{\mathbf{u}}^{(k)}\right)^\intercal\mathbf{X}_i\|_{\psi_2}\|(\widehat{\boldsymbol{\beta}}^{(k)}-\boldsymbol{\beta}_0)^\intercal\mathbf{X}_i\|_{\psi_2}\\ \leq&M\sqrt{n}\|\widehat{\mathbf{u}}^{(k)}-\mathbf{u}_0\|_2\|\widehat{\boldsymbol{\beta}}^{(k)}-\boldsymbol{\beta}_0\|_2\sigma_{\mathsf{max}}^2.\end{aligned}$$

This bound immediately implies

$$T_2=O_p\left(\sqrt{n}\|\widehat{\boldsymbol{\beta}}-\boldsymbol{\beta}_0\|_2\|\widehat{\mathbf{u}}^{(k)}-\mathbf{u}_0\|_2\right).$$

To show

$$\begin{aligned}T_3=&\sqrt{n}\widehat{\mathbf{u}}^{(k)\intercal}\left[\mathbb{E}\{\dot{\boldsymbol{\ell}}^{\dagger(k)}(\widehat{\boldsymbol{\beta}}^{(k)};\widehat{\boldsymbol{\gamma}}^{(k)})\mid\mathscr{D}_k^c\}-\left\{\dot{\boldsymbol{\ell}}^{\dagger(k)}(\widehat{\boldsymbol{\beta}}^{(k)};\widehat{\boldsymbol{\gamma}}^{(k)})-\dot{\boldsymbol{\ell}}^{\dagger(k)}(\boldsymbol{\beta}_0;\boldsymbol{\gamma}_0)\right\}\right]\\ =&O_p\left(\rho\|\widehat{\boldsymbol{\beta}}^{(k)}-\boldsymbol{\beta}_0\|_2+\sqrt{\rho(1-\rho)}\|\widehat{\boldsymbol{\gamma}}^{(k)}-\boldsymbol{\gamma}_0\|_2\right),\end{aligned}$$

we rewrite the term as two empirical processes with diminishing summands

$$
\begin{aligned}
T_3 = & -\sqrt{n}\frac{1}{N_k}\sum_{i\in\mathcal{J}_k}\Bigg(\widehat{\mathbf{u}}^{(k)\intercal}\mathbf{X}_i\{g(\widehat{\boldsymbol{\beta}}^{(k)\intercal}\mathbf{X}_i) - g(\boldsymbol{\beta}_0^\intercal\mathbf{X}_i) - g(\widehat{\boldsymbol{\gamma}}^{(k)\intercal}\mathbf{W}_i) + g(\boldsymbol{\gamma}_0^\intercal\mathbf{W}_i)\} \\
& - \mathbb{E}_{i\in\mathcal{J}_k}\left[\widehat{\mathbf{u}}^{(k)\intercal}\mathbf{X}_i\{g(\widehat{\boldsymbol{\beta}}^{(k)\intercal}\mathbf{X}_i) - g(\boldsymbol{\beta}_0^\intercal\mathbf{X}_i) - g(\widehat{\boldsymbol{\gamma}}^{(k)\intercal}\mathbf{W}_i) + g(\boldsymbol{\gamma}_0^\intercal\mathbf{W}_i)\} \mid \mathscr{D}_k^c\right]\Bigg) \\
& - \sqrt{n}\frac{1}{N_k}\sum_{i\in\mathcal{I}_k}\Bigg(\widehat{\mathbf{u}}^{(k)\intercal}\mathbf{X}_i\{g(\widehat{\boldsymbol{\beta}}^{(k)\intercal}\mathbf{X}_i) - g(\boldsymbol{\beta}_0^\intercal\mathbf{X}_i)\} \\
& - \mathbb{E}_{i\in\mathcal{J}_k}\left[\widehat{\mathbf{u}}^{(k)\intercal}\mathbf{X}_i\{g(\widehat{\boldsymbol{\beta}}^{(k)\intercal}\mathbf{X}_i) - g(\boldsymbol{\beta}_0^\intercal\mathbf{X}_i)\} \mid \mathscr{D}_k^c\right]\Bigg).
\end{aligned}
$$

We have used the identity $\mathbb{E}\{\dot{\boldsymbol{\ell}}^{\dagger(k)}(\boldsymbol{\beta}_0;\boldsymbol{\gamma}_0) \mid \mathscr{D}_k^c\} = 0$ above. Using Lemmas 14, 17-h and Assumptions (2a) and (2a), we show that each summand is sub-exponential

$$
\begin{aligned}
& \|\widehat{\mathbf{u}}^{(k)\intercal}\mathbf{X}_i\{g(\widehat{\boldsymbol{\beta}}^{(k)\intercal}\mathbf{X}_i) - g(\boldsymbol{\beta}_0^\intercal\mathbf{X}_i) - g(\widehat{\boldsymbol{\gamma}}^{(k)\intercal}\mathbf{W}_i) + g(\boldsymbol{\gamma}_0^\intercal\mathbf{W}_i)\}\|_{\psi_1} \\
\le & \|\widehat{\mathbf{u}}^{(k)\intercal}\mathbf{X}_i\|_{\psi_2}\|g(\widehat{\boldsymbol{\beta}}^{(k)\intercal}\mathbf{X}_i) - g(\boldsymbol{\beta}_0^\intercal\mathbf{X}_i) - g(\widehat{\boldsymbol{\gamma}}^{(k)\intercal}\mathbf{W}_i) + g(\boldsymbol{\gamma}_0^\intercal\mathbf{W}_i)\|_{\psi_2} \\
\le & M\sigma_{\mathsf{max}}^2\|\widehat{\mathbf{u}}^{(k)}\|_2\left(\|\widehat{\boldsymbol{\beta}}^{(k)} - \boldsymbol{\beta}_0\|_2 + \|\widehat{\boldsymbol{\gamma}}^{(k)} - \boldsymbol{\gamma}_0\|_2\right), \\
& \|\widehat{\mathbf{u}}^{(k)\intercal}\mathbf{X}_i\{g(\widehat{\boldsymbol{\beta}}^{(k)\intercal}\mathbf{X}_i) - g(\boldsymbol{\beta}_0^\intercal\mathbf{X}_i)\}\|_{\psi_1} \le M\sigma_{\mathsf{max}}^2\|\widehat{\mathbf{u}}^{(k)}\|_2\|\widehat{\boldsymbol{\beta}}^{(k)} - \boldsymbol{\beta}_0\|_2/2.
\end{aligned}
$$

Applying the Bernstein's inequality, we obtain

$$
T_3 = O_p\left(\|\widehat{\mathbf{u}}^{(k)}\|_2\left\{\sqrt{\rho}\|\widehat{\boldsymbol{\beta}}^{(k)} - \boldsymbol{\beta}_0\|_2 + \sqrt{\rho(1-\rho)}\|\widehat{\boldsymbol{\gamma}}^{(k)} - \boldsymbol{\gamma}_0\|_2\right\}\right).
$$

We achieve the stated rate with the tightness of $\|\widehat{\mathbf{u}}^{(k)}\|_2$ from (A.25).

To show

$$
\begin{aligned}
T_4 = & \sqrt{n}(1-\rho)\widehat{\mathbf{u}}^{(k)\intercal}\left[\mathbb{E}\{\dot{\boldsymbol{\ell}}_{\mathsf{imp}}^{(k)}(\widehat{\boldsymbol{\gamma}}^{(k)}) \mid \mathscr{D}_k^c\} - \left\{\dot{\boldsymbol{\ell}}_{\mathsf{imp}}^{(k)}(\widehat{\boldsymbol{\gamma}}^{(k)}) - \dot{\boldsymbol{\ell}}_{\mathsf{imp}}^{(k)}(\boldsymbol{\gamma}_0)\right\}\right] \\
= & O_p\left((1-\rho)\|\widehat{\boldsymbol{\gamma}}^{(k)} - \boldsymbol{\gamma}_0\|_2\right),
\end{aligned}
$$

we rewrite the term as the empirical process with diminishing summands

$$
\begin{aligned}
T_4 = & -\sqrt{n}(1-\rho)\frac{1}{n_k}\sum_{i\in\mathcal{I}_k}\Bigg(\widehat{\mathbf{u}}^{(k)\intercal}\mathbf{X}_i\{g(\widehat{\boldsymbol{\gamma}}^{(k)\intercal}\mathbf{W}_i) - g(\boldsymbol{\gamma}_0^\intercal\mathbf{W}_i)\} \\
& - \mathbb{E}_{i\in\mathcal{J}_k}\left[\widehat{\mathbf{u}}^{(k)\intercal}\mathbf{X}_i\{g(\widehat{\boldsymbol{\gamma}}^{(k)\intercal}\mathbf{W}_i) - g(\boldsymbol{\gamma}_0^\intercal\mathbf{W}_i)\} \mid \mathscr{D}_k^c\right]\Bigg).
\end{aligned}
$$

We have used the identity $\mathbb{E}\{\dot{\boldsymbol{\ell}}_{\mathsf{imp}}^{(k)}(\boldsymbol{\gamma}_0) \mid \mathscr{D}_k^c\} = 0$ above. Similar to the analysis of T_3, we show that each summand is sub-exponential

$$
\|\widehat{\mathbf{u}}^{(k)\intercal}\mathbf{X}_i\{g(\widehat{\boldsymbol{\gamma}}^{(k)\intercal}\mathbf{W}_i) - g(\boldsymbol{\gamma}_0^\intercal\mathbf{W}_i)\}\|_{\psi_1} \le M\sigma_{\mathsf{max}}^2\|\widehat{\mathbf{u}}^{(k)}\|_2\|\widehat{\boldsymbol{\gamma}}^{(k)} - \boldsymbol{\gamma}_0\|_2/2.
$$

Applying the Bernstein's inequality, we obtain

$$
T_4 = O_p\left((1-\rho)\|\widehat{\mathbf{u}}^{(k)}\|_2\|\widehat{\boldsymbol{\gamma}}^{(k)} - \boldsymbol{\gamma}_0\|_2\right).
$$

We achieve the stated rate with the tightness of $\|\widehat{\mathbf{u}}^{(k)}\|_2$ from (A.25).

To show

$$T_5 = \sqrt{n}\left(\mathbf{u}_0 - \widehat{\mathbf{u}}^{(k)}\right)^\intercal \left\{\dot{\boldsymbol{\ell}}^{\dagger(k)}(\boldsymbol{\beta}_0;\boldsymbol{\gamma}_0) + (1-\rho)\dot{\boldsymbol{\ell}}^{(k)}_{\mathsf{imp}}(\boldsymbol{\gamma}_0)\right\} = O_p\left(\|\widehat{\mathbf{u}}^{(k)} - \mathbf{u}_0\|_2\right),$$

we rewrite the term as the empirical process with diminishing summands

$$\begin{aligned}T_5 = &- \sqrt{n}\frac{1}{N_k}\sum_{i\in\mathcal{J}_k}(\widehat{\mathbf{u}}^{(k)} - \mathbf{u}_0)^\intercal \mathbf{X}_i\{g(\boldsymbol{\beta}_0^\intercal \mathbf{X}_i) - g(\boldsymbol{\gamma}_0^\intercal \mathbf{W}_i)\}\\ &- \sqrt{n}\frac{1}{n_k}\sum_{i\in\mathcal{I}_k}(\widehat{\mathbf{u}}^{(k)} - \mathbf{u}_0)^\intercal \mathbf{X}_i\{\rho\cdot g(\boldsymbol{\beta}_0^\intercal \mathbf{X}_i) + (1-\rho)\cdot g(\boldsymbol{\gamma}_0^\intercal \mathbf{W}_i) - Y_i\}.\end{aligned}$$

The summands have zero mean because

$$\begin{aligned}&\mathbb{E}_{i\in\mathcal{J}_k}\left[(\widehat{\mathbf{u}}^{(k)} - \mathbf{u}_0)^\intercal \mathbf{X}_i\{g(\boldsymbol{\beta}_0^\intercal \mathbf{X}_i) - g(\boldsymbol{\gamma}_0^\intercal \mathbf{W}_i)\} \mid \mathscr{D}_k^c\right]\\ =&(\widehat{\mathbf{u}}^{(k)} - \mathbf{u}_0)^\intercal \mathbb{E}\left[\mathbf{X}_i\{g(\boldsymbol{\beta}_0^\intercal \mathbf{X}_i) - g(\boldsymbol{\gamma}_0^\intercal \mathbf{W}_i)\}\right]\\ =&0,\\ &\mathbb{E}_{i\in\mathcal{I}_k}\left[(\widehat{\mathbf{u}}^{(k)} - \mathbf{u}_0)^\intercal \mathbf{X}_i\{\rho\cdot g(\boldsymbol{\beta}_0^\intercal \mathbf{X}_i) + (1-\rho)\cdot g(\boldsymbol{\gamma}_0^\intercal \mathbf{W}_i) - Y_i\} \mid \mathscr{D}_k^c\right]\\ =&(\widehat{\mathbf{u}}^{(k)} - \mathbf{u}_0)^\intercal \left(\rho\mathbb{E}\left[\mathbf{X}_i\{g(\boldsymbol{\beta}_0^\intercal \mathbf{X}_i) - Y_i\}\right] + (1-\rho)\mathbb{E}\left[\mathbf{X}_i\{g(\boldsymbol{\gamma}_0^\intercal \mathbf{W}_i) - Y_i\}\right]\right)\\ =&0.\end{aligned}$$

Similar to the analysis of T_3, we show that each summand is sub-exponential

$$\left\|(\widehat{\mathbf{u}}^{(k)} - \mathbf{u}_0)^\intercal \mathbf{X}_i\{g(\boldsymbol{\beta}_0^\intercal \mathbf{X}_i) - g(\boldsymbol{\gamma}_0^\intercal \mathbf{W}_i)\}\right\|_{\psi_1} \le \sqrt{2}\sigma_{\mathsf{max}}(\nu_1\vee\nu_2)\|\widehat{\mathbf{u}}^{(k)} - \mathbf{u}_0\|_2$$
$$\left\|(\widehat{\mathbf{u}}^{(k)} - \mathbf{u}_0)^\intercal \mathbf{X}_i\{\rho\cdot g(\boldsymbol{\beta}_0^\intercal \mathbf{X}_i) + (1-\rho)\cdot g(\boldsymbol{\gamma}_0^\intercal \mathbf{W}_i) - Y_i\}\right\|_{\psi_1} \le \sqrt{2}\sigma_{\mathsf{max}}(\nu_1\vee\nu_2)\|\widehat{\mathbf{u}}^{(k)} - \mathbf{u}_0\|_2$$

Applying the Bernstein's inequality, we obtain

$$T_5 = O_p\left(\|\widehat{\mathbf{u}}^{(k)} - \mathbf{u}_0\|_2\left\{\sqrt{\rho(1-\rho)}+1\right\}\right) = O_p\left(\|\widehat{\mathbf{u}}^{(k)} - \mathbf{u}_0\|_2\right).$$

ANALYSIS FOR TERMS T_1'-T_4' IN PART 2

Conditionally on the out-of-fold data, the term T_1' is the empirical average of i.i.d. mean zero random variables,

$$\begin{aligned}T_1 =&\sum_{k=1}^K\frac{n_k}{n}\left(\frac{1}{n_k}\sum_{i\in\mathcal{I}_k}(\widehat{\mathbf{u}}^{(k)\intercal}\mathbf{X}_i)^2\{(1-\rho)\cdot g(\widehat{\boldsymbol{\gamma}}^{(k)\intercal}\mathbf{W}_i) + \rho\cdot g(\widehat{\boldsymbol{\beta}}^{(k)\intercal}\mathbf{X}_i) - Y_i\}^2\right.\\ &\left.- \mathbb{E}_{i\in\mathcal{I}_k}\left[(\widehat{\mathbf{u}}^{(k)\intercal}\mathbf{X}_i)^2\{(1-\rho)\cdot g(\widehat{\boldsymbol{\gamma}}^{(k)\intercal}\mathbf{W}_i) + \rho\cdot g(\widehat{\boldsymbol{\beta}}^{(k)\intercal}\mathbf{X}_i) - Y_i\}^2 \mid \mathscr{D}_k^c\right]\right).\end{aligned}$$

We bound the variance of each summand by the Cauchy-Schwartz inequality and Lemmas 17-b, 17-d, 17-e,

$$\underset{i\in\mathcal{I}_k}{\mathrm{Var}}\left[(\widehat{\mathbf{u}}^{(k)\intercal}\mathbf{X}_i)^2\{(1-\rho)\cdot g(\widehat{\boldsymbol{\gamma}}^{(k)\intercal}\mathbf{W}_i) + \rho\cdot g(\widehat{\boldsymbol{\beta}}^{(k)\intercal}\mathbf{X}_i) - Y_i\}^2 \mid \mathscr{D}_k^c\right]$$

$$
\begin{aligned}
&\leq \mathbb{E}_{i\in\mathcal{I}_k}\left[(\widehat{\mathbf{u}}^{(k)\intercal}\mathbf{X}_i)^4\{(1-\rho)\cdot g(\widehat{\boldsymbol{\gamma}}^{(k)\intercal}\mathbf{W}_i)+\rho\cdot g(\widehat{\boldsymbol{\beta}}^{(k)\intercal}\mathbf{X}_i)-Y_i\}^4 \mid \mathscr{D}_k^c\right] \\
&\leq \sqrt{\mathbb{E}_{i\in\mathcal{I}_k}\left\{(\widehat{\mathbf{u}}^{(k)\intercal}\mathbf{X}_i)^8 \mid \mathscr{L}_k^c\right\}\mathbb{E}_{i\in\mathcal{I}_k}\left[\{(1-\rho)\cdot g(\widehat{\boldsymbol{\gamma}}^{(k)\intercal}\mathbf{W}_i)+\rho\cdot g(\widehat{\boldsymbol{\beta}}^{(k)\intercal}\mathbf{X}_i)-Y_i\}^8 \mid \mathscr{D}_k^c\right]} \\
&\leq \sqrt{48\|\widehat{\mathbf{u}}^{(k)\intercal}\mathbf{X}_i\|_{\psi_2}^8 48\left(\rho\|g(\widehat{\boldsymbol{\beta}}^{(k)\intercal}\mathbf{X}_i)-Y_i\|_{\psi_2}\vee(1-\rho)\|g(\widehat{\boldsymbol{\gamma}}^{(k)\intercal}\mathbf{X}_i)-Y_i\|_{\psi_2}\right)^8}
\end{aligned}
\tag{A.52}
$$

Under Assumption 1a, 1b, 2a, we have

$$
\|\widehat{\mathbf{u}}^{(k)\intercal}\mathbf{X}_i\|_{\psi_2} \leq (\|\mathbf{u}_0\|_2+\|\widehat{\mathbf{u}}^{(k)}-\mathbf{u}_0\|_2)\sigma_{\mathsf{max}}/\sqrt{2} = O_p\left(1+\|\widehat{\mathbf{u}}^{(k)}-\mathbf{u}_0\|_2\right).
$$

We apply Lemma 14 to obtain

$$
\begin{aligned}
\|g(\widehat{\boldsymbol{\beta}}^{(k)\intercal}\mathbf{X}_i)-Y_i\|_{\psi_2} &= O_p\left(1+\|\widehat{\boldsymbol{\beta}}^{(k)}-\boldsymbol{\beta}_0\|_2\right), \\
\|g(\widehat{\boldsymbol{\gamma}}^{(k)\intercal}\mathbf{W}_i)-Y_i\|_{\psi_2} &= O_p\left(1+\|\widehat{\boldsymbol{\gamma}}^{(k)}-\boldsymbol{\gamma}_0\|_2\right).
\end{aligned}
$$

We have shown that the variance in (A.52) is of order

$$
O_p\left(1+\|\widehat{\mathbf{u}}^{(k)}-\mathbf{u}_0\|_2^4+\rho^4\|\widehat{\boldsymbol{\beta}}^{(k)}-\boldsymbol{\beta}_0\|_2^4+(1-\rho)^4\|\widehat{\boldsymbol{\gamma}}^{(k)}-\boldsymbol{\gamma}_0\|_2^4\right).
$$

Thus by the Tchebychev's inequality, we obtain

$$
T_1' = O_p\left(\left\{1+\|\widehat{\mathbf{u}}^{(k)}-\mathbf{u}_0\|_2+\rho\|\widehat{\boldsymbol{\beta}}^{(k)}-\boldsymbol{\beta}_0\|_2+(1-\rho)\|\widehat{\boldsymbol{\gamma}}^{(k)}-\boldsymbol{\gamma}_0\|_2\right\}/\sqrt{n}\right)
$$

Applying the consistency of $\widehat{\boldsymbol{\gamma}}^{(k)}$, $\widehat{\boldsymbol{\beta}}^{(k)}$ and $\widehat{\mathbf{u}}^{(k)}$ from (A.22)

$$
T_1' = O_p\left(n^{-1/2}\right) = o_p(1).
$$

To analyze T_2', we consider the decomposition in which the estimators are replaced by the estimands one by one,

$$
\begin{aligned}
&T_2' \\
=&\sum_{k=1}^K \frac{n_k}{n}\Bigg(\mathbb{E}_{i\in\mathcal{I}_k}\left[(\widehat{\mathbf{u}}^{(k)\intercal}\mathbf{X}_i)^2\{(1-\rho)\cdot g(\widehat{\boldsymbol{\gamma}}^{(k)\intercal}\mathbf{W}_i)+\rho\cdot g(\widehat{\boldsymbol{\beta}}^{(k)\intercal}\mathbf{X}_i)-Y_i\}^2 \mid \mathscr{D}_k^c\right] \\
&\quad -\mathbb{E}\left[(\mathbf{u}_0^\intercal\mathbf{X}_i)^2\{(1-\rho)\cdot g(\boldsymbol{\gamma}_0^\intercal\mathbf{W}_i)+\rho\cdot g(\boldsymbol{\beta}_0^\intercal\mathbf{X}_i)-Y_i\}^2\right]\Bigg) \\
=&\sum_{k=1}^K \frac{n_k}{n}\mathbb{E}_{i\in\mathcal{I}_k}\left[\{(\widehat{\mathbf{u}}^{(k)}-\mathbf{u}_0)^\intercal\mathbf{X}_i\}\widehat{\mathbf{u}}^{(k)\intercal}\mathbf{X}_i\{(1-\rho)\cdot g(\widehat{\boldsymbol{\gamma}}^{(k)\intercal}\mathbf{W}_i)+\rho\cdot g(\widehat{\boldsymbol{\beta}}^{(k)\intercal}\mathbf{X}_i)-Y_i\}^2 \mid \mathscr{D}_k^c\right] \\
&+\sum_{k=1}^K \frac{n_k}{n}\mathbb{E}_{i\in\mathcal{I}_k}\left[\{(\widehat{\mathbf{u}}^{(k)}-\mathbf{u}_0)^\intercal\mathbf{X}_i\}\mathbf{u}_0^\intercal\mathbf{X}_i\{(1-\rho)\cdot g(\widehat{\boldsymbol{\gamma}}^{(k)\intercal}\mathbf{W}_i)+\rho\cdot g(\widehat{\boldsymbol{\beta}}^{(k)\intercal}\mathbf{X}_i)-Y_i\}^2 \mid \mathscr{D}_k^c\right] \\
&+\sum_{k=1}^K \frac{n_k}{n}\mathbb{E}_{i\in\mathcal{I}_k}\Big[(\mathbf{u}_0^\intercal\mathbf{X}_i)^2\{(1-\rho)\cdot g(\widehat{\boldsymbol{\gamma}}^{(k)\intercal}\mathbf{W}_i)+\rho\cdot g(\widehat{\boldsymbol{\beta}}^{(k)\intercal}\mathbf{X}_i)-Y_i\}
\end{aligned}
$$

$$\times \left[(1-\rho)\{g(\widehat{\boldsymbol{\gamma}}^{(k)\intercal}\mathbf{W}_i) - g(\boldsymbol{\gamma}_0^\intercal\mathbf{W}_i)\} + \rho\{g(\widehat{\boldsymbol{\beta}}^{(k)\intercal}\mathbf{X}_i) - g(\boldsymbol{\beta}_0^\intercal\mathbf{X}_i)\}\right] \mid \mathscr{D}_k^c\Big]$$

$$+\sum_{k=1}^K \frac{n_k}{n}\mathbb{E}_{i\in\mathcal{I}_k}\Big[(\mathbf{u}_0^\intercal\mathbf{X}_i)^2\{(1-\rho)\cdot g(\boldsymbol{\gamma}_0^\intercal\mathbf{W}_i) + \rho\cdot g(\boldsymbol{\beta}_0^\intercal\mathbf{X}_i) - Y_i\}$$

$$\times \left[(1-\rho)\{g(\widehat{\boldsymbol{\gamma}}^{(k)\intercal}\mathbf{W}_i) - g(\boldsymbol{\gamma}_0^\intercal\mathbf{W}_i)\} + \rho\{g(\widehat{\boldsymbol{\beta}}^{(k)\intercal}\mathbf{X}_i) - g(\boldsymbol{\beta}_0^\intercal\mathbf{X}_i)\}\right] \mid \mathscr{D}_k^c\Big].$$

Following the same calculation as in (A.52), we can bound the expectations

$$T_2' = O_p\left(\|\widehat{\mathbf{u}}^{(k)} - \mathbf{u}_0\|_2 + \rho\|\widehat{\boldsymbol{\beta}}^{(k)} - \boldsymbol{\beta}_0\|_2 + (1-\rho)\|\widehat{\boldsymbol{\gamma}}^{(k)} - \boldsymbol{\gamma}_0\|_2\right)$$

Applying the consistency of $\widehat{\boldsymbol{\gamma}}$, $\widehat{\boldsymbol{\beta}}$ and $\widehat{\mathbf{u}}$ from Lemma 21, Theorem 1 and Part 1 in the proof of Theorem 7, we have established

$$T_2' = o_p(1).$$

Repeating the analyses for T_1' and T_2', we can show

$$T_3' = O_p\left(\rho\sqrt{(1-\rho)/N}\left\{1 + \|\widehat{\mathbf{u}}^{(k)} - \mathbf{u}_0\|_2 + \|\widehat{\boldsymbol{\beta}}^{(k)} - \boldsymbol{\beta}_0\|_2 + \|\widehat{\boldsymbol{\gamma}}^{(k)} - \boldsymbol{\gamma}_0\|_2\right\}\right) = o_p(1),$$

$$T_4' = O_p\left(\rho(1-\rho)\left\{\|\widehat{\mathbf{u}}^{(k)} - \mathbf{u}_0\|_2 + \|\widehat{\boldsymbol{\beta}}^{(k)} - \boldsymbol{\beta}_0\|_2 + \|\widehat{\boldsymbol{\gamma}}^{(k)} - \boldsymbol{\gamma}_0\|_2\right\}\right) = o_p(1)$$

Appendix D. Additional Technical Details

D1 Definitions

We adopt the following definition of sub-Gaussian and sub-exponential random variables.

Definition 15 (Sub-Gaussian and Sub-Exponential Random Variables) *The sub-Gaussian parameter for a random variable V is defined as*

$$\|V\|_{\psi_2} = \inf\left\{\sigma > 0 : \mathbb{E}(e^{V^2/\sigma^2}) \le 2\right\}.$$

The random variable V is sub-Gaussian if $\|V\|_{\psi_2}$ is finite. The sub-Gaussian parameter for a random vector $\mathbf{U}$ is defined as

$$\|\mathbf{U}\|_{\psi_2} = \sup_{\|\mathbf{v}\|_2=1} \|\mathbf{v}^\intercal\mathbf{U}\|_{\psi_2}.$$

The sub-Gaussian parameter for a random variable V is defined as

$$\|V\|_{\psi_1} = \inf\left\{\nu > 0 : \mathbb{E}(e^{|V|/\nu}) \le 2\right\}.$$

The random variable V is sub-exponential if $\|V\|_{\psi_1}$ is finite. The more general Orlicz norm for $\alpha \in (0,1)$ is defined as

$$\|V\|_{\psi_\alpha} = \inf\left\{\nu > 0 : \mathbb{E}\left[e^{(|V|/\nu)^\alpha}\right] \le 2\right\}.$$

Mimicking the (minimal) Restricted Eigenvalue condition on the minimal eigenvalue of matrix over a cone (Bickel et al., 2009), we define the maximal Restricted Eigenvalue in Definition 16.

Definition 16 (Maximal Restricted Eigenvalue) *For a cone-set of the indices set $\mathcal{O} \subset \{1, \dots, p\}$*

$$\mathcal{C}_\gamma(\xi, \mathcal{O}) := \left\{ v \in \mathbb{R}^{p+q+1} : \|v_{\mathcal{O}^c}\|_1 \le \xi \|v_{\mathcal{O}}\|_1 \right\}, \tag{A.53}$$

we define the maximal Restricted Eigenvalue of a matrix Σ as

$$\mathrm{RE}_{\max}(\xi, \mathcal{O}; \Sigma) = \sup_{v \in \mathcal{C}_\gamma(\xi,\mathcal{O}) \setminus \{0\}} \frac{\sqrt{v^\intercal \Sigma v}}{\|v\|_2}. \tag{A.54}$$

D2 Statements of Existing Results

The properties in Lemmas 17 and 18 are covered in Vershynin (2018) Chapter 2 and 4.

Lemma 17 (Properties of sub-Gaussian and sub-exponential random variables)

a) Tail-probability:

$$\mathbb{P}(|V| \ge x) \le 2e^{-x/\|V\|_{\psi_1}},$$
$$\mathbb{P}(|V| \ge x) \le 2e^{-x^2/\|V\|_{\psi_2}^2};$$

b) Moments: $\mathbb{E}(|V|^r) \le \min\{\kappa_{\psi,1}\|V\|_{\psi_1}^r, \kappa_{\psi,2}\|V\|_{\psi_2}^r\}$ with $\kappa_{\psi,1} = r!2$ and $\kappa_{\psi,2} = \Gamma(r/2)r$, and $\mathbb{E}(|V|) \le \sqrt{\pi}\|V\|_{\psi_2}$;

c) Hierarchy: $\|V\|_{\psi_1} \le \|V\|_{\psi_2}$;

d) Arbitrary addition: $\|\sum_{i=1}^m V_i\|_{\psi_2} \le m \max_{i=1,\dots,m} \|V_i\|_{\psi_2}$ and $\|\sum_{i=1}^m V_i\|_{\psi_1} \le m \max_{i=1,\dots,m} \|V_i\|_{\psi_1}$;

e) Multiplication with bounded random variable: $\|V_1V_2\|_{\psi_2} \le \|V_1\|_{\psi_2}K$, $\|V_1V_2\|_{\psi_1} \le \|V_1\|_{\psi_1}K$ for $|V_2| \le K$ almost surely;

f) Multiplication between sub-Gaussian random variables: $\|V_1V_2\|_{\psi_1} \le \|V_1\|_{\psi_2}\|V_2\|_{\psi_2}$, in particular, $\|V_1\|_{\psi_1} \le \|V_1\|_{\psi_2}/\sqrt{\log(2)}$;

g) Hoeffding's inequality: $V_1, \dots, V_m$ are independent mean zero sub-Gaussian random variables. For $t > 0$,

$$\mathbb{P}\left(\left| \sum_{i=1}^m V_i \right| \ge t \right) \le 4 \exp\left(-\frac{t^2}{\kappa_{\psi,3} \sum_{i=1}^m \|V_i\|_{\psi_2}^2} \right), \ \kappa_{\psi,3} = 8.$$

h) Bernstein's inequality: $V_1, \dots, V_m$ are independent mean zero sub-exponential random variables. For $t > 0$, $\kappa_{\psi,4} = 16$ and $\kappa_{\psi,5} = 4$

$$\mathbb{P}\left(\left| \sum_{i=1}^m V_i \right| \ge t \right) \le 2 \exp\left[-\min\left\{ t^2 \left(\kappa_{\psi,4} \sum_{i=1}^m \|V_i\|_{\psi_1}^2 \right)^{-1}, t \left(\kappa_{\psi,5} \max_{i=1,\dots,m} \|V_i\|_{\psi_1} \right)^{-1} \right\} \right].$$

Lemma 18 *Let $V_1, \dots, V_m$ be i.i.d sub-Gaussian vectors in $\mathbb{R}^p$ such that*

$$\|v^\intercal V\|_{\psi_2}^2 \le K^2 \mathbb{E}\{(v^\intercal V)^2\}$$

for some $1 \le K < \infty$. Then,

$$\left\| \frac{1}{m} \sum_{i=1}^m V_i V_i^\intercal - \mathbb{E}(VV^\intercal) \right\|_2 = O_p\left(p/m + \sqrt{p/m}\right).$$

From Negahban et al. (2010) and Huang and Zhang (2012) among other literatures, we have the following results concerning the LASSO under the generalized linear models.

Lemma 19 *Under Assumptions 1b, 2a and 2b,*

$$\mathbb{P}\Big(\ell_{\mathsf{imp}}(\boldsymbol{\gamma}_0 + \boldsymbol{\Delta}) - \ell_{\mathsf{imp}}(\boldsymbol{\gamma}_0) - \boldsymbol{\Delta}^\intercal \dot{\boldsymbol{\ell}}_{\mathsf{imp}}(\boldsymbol{\gamma}_0) \\ \ge \kappa_{\mathsf{rsc},1}\|\boldsymbol{\Delta}\|_2\{\|\boldsymbol{\Delta}\|_2 - \kappa_{\mathsf{rsc},2}\sqrt{\log(p+q)/n}\|\boldsymbol{\Delta}\|_1\}, \forall \|\boldsymbol{\Delta}\|_2 \le 1 \Big) \ge 1 - \kappa_{\mathsf{rsc},3} e^{-\kappa_{\mathsf{rsc},4} n};$$

$$\mathbb{P}\Big(\ell_{\mathsf{PL}}(\boldsymbol{\beta}_0 + \boldsymbol{\Delta}) - \ell_{\mathsf{PL}}(\boldsymbol{\beta}_0) - \boldsymbol{\Delta}^\intercal \dot{\boldsymbol{\ell}}_{\mathsf{PL}}(\boldsymbol{\beta}_0) \\ \ge \kappa_{\mathsf{rsc},1}\|\boldsymbol{\Delta}\|_2\{\|\boldsymbol{\Delta}\|_2 - \kappa_{\mathsf{rsc},2}\sqrt{\log(p)/N}\|\boldsymbol{\Delta}\|_1\}, \forall \|\boldsymbol{\Delta}\|_2 \le 1 \Big) \ge 1 - \kappa_{\mathsf{rsc},3} e^{-\kappa_{\mathsf{rsc},4} N}.$$

The negative log-likelihoods are defined in (8) *and* (10)*, and their gradients defined in* (12)*. See Definition 11 for the definition of conditional expectation notation. The constants are all absolute.*

The two inequalities in Lemma 19 are direct application of Negahban et al. (2010) Proposition 2 page 22. We can construct an auxiliary loss function to prove the following lemma.

Lemma 20 *Under Assumptions 1b, 2a and 2b,*

$$\mathbb{P}\Big(\frac{1}{N_{k'}} \sum_{i \in \mathcal{I}_{k'} \cup \mathcal{J}_{k'}} g'\left(\widehat{\boldsymbol{\beta}}^{(k,k')\intercal} \mathbf{X}_i\right) (\boldsymbol{\Delta}^\intercal \mathbf{X}_i)^2 \\ \ge 2\kappa^*_{\mathsf{rsc},1}\|\boldsymbol{\Delta}\|_2^2 - \kappa^*_{\mathsf{rsc},1}\kappa^*_{\mathsf{rsc},2}\sqrt{\log(p)/N}\|\boldsymbol{\Delta}\|_2\|\boldsymbol{\Delta}\|_1, \forall \|\boldsymbol{\Delta}\|_2 \le 1 \Big) \\ \ge \mathbb{P}\left(\left\|\widehat{\boldsymbol{\beta}}^{(k)} - \boldsymbol{\beta}_0\right\|_2 \le \frac{\sigma_{\mathsf{min}}^2}{2\sigma_{\mathsf{max}}^3} \right) - \kappa^*_{\mathsf{rsc},3} e^{-\kappa^*_{\mathsf{rsc},4} N}.$$

The constants are all absolute.

Proof [Proof of Lemma 20] First, we show $\sqrt{g'\left(\widehat{\boldsymbol{\beta}}^{(k,k')\intercal}\mathbf{X}_i\right)}\mathbf{X}_i$ is a sub-Gaussian random vector whose second moment has all eigenvalues bounded away from infinity and zero. Under Assumptions 1b and 2a, we may apply Lemma 17-e,

$$\left\| \mathbf{v}^\intercal \sqrt{g'\left(\widehat{\boldsymbol{\beta}}^{(k,k')\intercal}\mathbf{X}_i\right)}\mathbf{X}_i \right\|_{\psi_2} \le \sqrt{M}\|\mathbf{v}^\intercal \mathbf{X}_i\|_{\psi_2} \le \sqrt{M}\sigma_{\mathsf{max}}\|\mathbf{v}\|_2/\sqrt{2}.$$

Thus, $\sqrt{g'\left(\widehat{\boldsymbol{\beta}}^{(k,k')\intercal}\mathbf{X}_i\right)}\mathbf{X}_i$ is a sub-Gaussian random vector. Under Assumptions 1b and 2a, we can bound the maximal eigenvalue of its second moment,

$$\mathbf{v}^\intercal\mathbb{E}_{i\in\mathcal{I}_{k'}\cup\mathcal{J}_{k'}}\left\{g'\left(\widehat{\boldsymbol{\beta}}^{(k,k')\intercal}\mathbf{X}_i\right)\mathbf{X}_i\mathbf{X}_i^\intercal \mid \mathscr{D}_{k'}^c\right\}\mathbf{v} \le M\mathbb{E}\{(\mathbf{v}^\intercal\mathbf{X}_i)^2\} \le M\|\mathbf{v}\|_2^2\sigma_{\mathsf{max}}^2.$$

We derive the lower bound for the minimal eigenvalue of its second moment from Assumptions 1b, 2a, 2a, 2b-i, the Cauchy-Schwartz inequality and Lemma 17-b,

$$\begin{aligned}
&\mathbf{v}^\intercal\mathbb{E}_{i\in\mathcal{I}_{k'}\cup\mathcal{J}_{k'}}\left\{g'\left(\widehat{\boldsymbol{\beta}}^{(k,k')\intercal}\mathbf{X}_i\right)\mathbf{X}_i\mathbf{X}_i^\intercal \mid \mathscr{D}_{k'}^c\right\}\mathbf{v}\\
\ge&\mathbf{v}^\intercal\mathbb{E}_{i\in\mathcal{I}_{k'}\cup\mathcal{J}_{k'}}\{g'(\boldsymbol{\beta}_0^\intercal\mathbf{X}_i)\mathbf{X}_i\mathbf{X}_i^\intercal \mid \mathscr{D}_{k'}^c\}\mathbf{v}\\
&-\mathbb{E}_{i\in\mathcal{I}_{k'}\cup\mathcal{J}_{k'}}\left[(\mathbf{v}^\intercal\mathbf{X}_i)^2\left\{g(\boldsymbol{\beta}_0^\intercal\mathbf{X}_i)-g\left(\widehat{\boldsymbol{\beta}}^{(k,k')\intercal}\mathbf{X}_i\right)\right\} \mid \mathscr{D}_{k'}^c\right]\\
\ge&\|\mathbf{v}\|_2^2\sigma_{\mathsf{min}}^2 - M\mathbb{E}_{i\in\mathcal{I}_{k'}\cup\mathcal{J}_{k'}}\left[\left|(\mathbf{v}^\intercal\mathbf{X}_i)^2\left\{\left(\boldsymbol{\beta}_0-\widehat{\boldsymbol{\beta}}^{(k,k')}\right)^\intercal\mathbf{X}_i\right\}\right| \mid \mathscr{D}_{k'}^c\right]\\
\ge&\|\mathbf{v}\|_2^2\sigma_{\mathsf{min}}^2 - M\sqrt{\mathbb{E}\{(\mathbf{v}^\intercal\mathbf{X}_i)^4\}\mathbb{E}_{i\in\mathcal{I}_{k'}\cup\mathcal{J}_{k'}}\left[\left\{\left(\boldsymbol{\beta}_0-\widehat{\boldsymbol{\beta}}^{(k,k')}\right)^\intercal\mathbf{X}_i\right\}^2 \mid \mathscr{D}_{k'}^c\right]}\\
\ge&\|\mathbf{v}\|_2^2\left(\sigma_{\mathsf{min}}^2 - M\sigma_{\mathsf{max}}^3\left\|\widehat{\boldsymbol{\beta}}^{(k,k')}-\boldsymbol{\beta}_0\right\|_2\right).
\end{aligned}$$

Whenever $\left\|\widehat{\boldsymbol{\beta}}^{(k)}-\boldsymbol{\beta}_0\right\|_2 \le \frac{\sigma_{\mathsf{min}}^2}{2\sigma_{\mathsf{max}}^3}$, we have

$$\mathbf{v}^\intercal\mathbb{E}_{i\in\mathcal{I}_{k'}\cup\mathcal{J}_{k'}}\left\{g'\left(\widehat{\boldsymbol{\beta}}^{(k,k')\intercal}\mathbf{X}_i\right)\mathbf{X}_i\mathbf{X}_i^\intercal \mid \mathscr{D}_{k'}^c\right\}\mathbf{v} \ge \|\mathbf{v}\|_2^2\sigma_{\mathsf{min}}^2/2.$$

Second, we construct an auxiliary least square loss to apply Negahban et al. (2010). Let ε_i be independent standard normal random variables. Construct the loss function

$$\mathcal{L}^{(k,k')}(\mathbf{v}) = \frac{1}{N_{k'}}\sum_{i\in\mathcal{I}_{k'}\cup\mathcal{J}_{k'}}\left\{\varepsilon_i + (\mathbf{v}_0-\mathbf{v})^\intercal\sqrt{g'\left(\widehat{\boldsymbol{\beta}}^{(k,k')\intercal}\mathbf{X}_i\right)}\mathbf{X}_i\right\}^2.$$

By the design, we have

$$\mathcal{L}^{(k,k')}(\mathbf{v}_0+\boldsymbol{\Delta}) - \mathcal{L}^{(k,k')}(\mathbf{v}_0) - \boldsymbol{\Delta}^\intercal\frac{\partial}{\partial\mathbf{v}}\mathcal{L}^{(k,k')}(\mathbf{v}_0+\boldsymbol{\Delta}) = \frac{1}{N_{k'}}\sum_{i\in\mathcal{I}_{k'}\cup\mathcal{J}_{k'}} g'\left(\widehat{\boldsymbol{\beta}}^{(k,k')\intercal}\mathbf{X}_i\right)(\boldsymbol{\Delta}^\intercal\mathbf{X}_i)^2.$$

We apply Proposition 2 in Negahban et al. (2010) for $\mathcal{L}^{(k,k')}(\mathbf{v})$ conditionally on out-of-fold data $\mathscr{D}_{k'}^c$ and the event $\left\{\left\|\widehat{\boldsymbol{\beta}}^{(k,k')}-\boldsymbol{\beta}_0\right\|_2 \le \frac{\sigma_{\mathsf{min}}^2}{2\sigma_{\mathsf{max}}^3}\right\}$ to finish the proof.

■

Lemma 21 *For a constant $\kappa_{\mathsf{cone}}(n,p,q,\varepsilon_r) \asymp \sqrt{s_\gamma\log(p+q)/n}$, the event*

$$\Omega_{\mathsf{cone}} = \left\{\|\dot{\boldsymbol{\ell}}_{\mathsf{imp}}(\boldsymbol{\gamma}_0)\|_\infty = \left\|\frac{1}{n}\sum_{i=1}^n\mathbf{W}_i\{g(\boldsymbol{\gamma}_0^\intercal\mathbf{W}_i)-Y_i\}\right\|_\infty \le \kappa_{\mathsf{cone}}(n,p,q,\varepsilon_r)\right\}$$

occur with probability greater than $1-\varepsilon_r$ *under Assumptions 1a and 2a. Setting* $\lambda_\gamma = 2$*, we have on event* Ω_{cone} *that*

$$\widehat{\gamma} - \boldsymbol{\gamma}_0 \in \mathcal{C}_\gamma(3, \mathrm{supp}(\boldsymbol{\gamma}_0)) = \left\{ v \in \mathbb{R}^{p+q+1} : \|v_{\mathcal{O}_\gamma^c}\|_1 \leq 3\|v_{\mathcal{O}_\gamma}\|_1 \right\},$$

where $\mathcal{O}_\gamma = \{j : \gamma_j \neq 0\}$ *is the indices set for nonzero coefficient in* $\boldsymbol{\gamma}_0$*. Moreover, we have*

$$\|\widehat{\gamma} - \boldsymbol{\gamma}_0\|_2 = O_p\left(\sqrt{s_\gamma \log(p+q)/n}\right).$$

The concentration on the event Ω_{cone} is established by the union bound of element wise concentration, which is in turn obtained by the Bernstein inequality for sub-exponential random variables (Lemma 17-h). The rest of Lemma 21 follows Huang and Zhang (2012) Lemma 1 page 5 (page 1843 of the issue) and Negahban et al. (2010) Corollary 5 page 23.
